# *trans*-Hydrogenation: Application to a Concise and Scalable Synthesis of Brefeldin A**

**DOI:** 10.1002/anie.201411618

**Published:** 2015-02-04

**Authors:** Michael Fuchs, Alois Fürstner

**Affiliations:** Max-Planck-Institut für Kohlenforschung45470 Mülheim/Ruhr (Germany)

**Keywords:** alkyne metathesis, hydrogenation, natural products, ruthenium, total synthesis

## Abstract

The important biochemical probe molecule brefeldin A (**1**) has served as an inspirational target in the past, but none of the many routes has actually delivered more than just a few milligrams of product, where documented. The approach described herein is clearly more efficient; it hinges upon the first implementation of ruthenium-catalyzed trans-hydrogenation in natural products total synthesis. Because this unorthodox reaction is selective for the triple bond and does not touch the transannular alkene or the lactone site of the cycloalkyne, it outperforms the classical Birch-type reduction that could not be applied at such a late stage. Other key steps en route to **1** comprise an iron-catalyzed reductive formation of a non-terminal alkyne, an asymmetric propiolate carbonyl addition mediated by a bulky amino alcohol, and a macrocyclization by ring-closing alkyne metathesis catalyzed by a molybdenum alkylidyne.

Few natural products have as illustrious a pedigree as brefeldin A (**1**), which had originally been isolated from *Penicillium decumbens* but was later also found in other fungal strains.[1] This macrolide is endowed with antifungal, antiviral, nematocidal, and antimitotic activity and had been selected by the National Cancer Institute for detailed preclinical survey for its ability to drive various human cancer cell lines into apoptosis.[2] Although this profile did not translate into a clinical success, **1** gained prominence in chemical biology and biomedical research for its stunning effects on intracellular protein trafficking. Upon incubation with **1**, eukaryotic cells rapidly disassemble the Golgi apparatus and redistribute its constituents into the endoplasmatic reticulum. This massive but reversible morphological change is caused by binding of **1** to a protein complex consisting of a catalytic guanine exchange factor (GEF) and the small G protein adenosine ribosylation factor 1 (ARF1), which exerts key regulatory functions for vesicle budding and transport.[3], [4] Two independent crystal structures showed that **1** inserts in a wedge-like manner at the interface of these proteins and thereby brings the GDP/GTP exchange critical for the proper functioning of the ARF1 GTPase to a halt.[5]

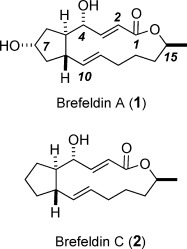


Equally rich is the synthetic record of brefeldin. More than 40 different strategies in pursuit of **1** or its less potent sibling **2** have been described over the past four decades.[6]–[12] Although many original solutions were found, several recurring themes can also be noticed in this impressive body of work. The most obvious one is the enduring dominance of macrolactonization for the formation of the 13-membered ring. Only a few macrocyclizations through C—C bond formation have been pursued with varying success,[8] with ring-closing olefin metathesis (RCM)[13] at the Δ^10, 11^ bond being the only catalytic method applied to date.[9] Since the current state of the art does not allow *E*-selectivity to be imposed on RCM by catalyst control,[14] the observed isomer ratios were case dependent and typically unfavorable.

Other groups chose to set the embedded *E*-olefins more concisely, for instance through the *trans*-reduction of an appropriate alkyne precursor. With one exception, where a two-step protocol of *trans*-hydrosilylation/proto-desilylation was pursued to form the enoate motif of **1**,[10] they all resorted to the use of alkali metals in liquid ammonia.[11] Because of the harsh conditions, this methodology necessitates considerable oxidation state and protecting group management en route to the final product and therefore needs to be carefully timed. We felt that the procedure for direct alkyne *trans*-hydrogenation recently disclosed by our group provides a larger window of opportunity and should qualify for applications to polyfunctional compounds where Birch reduction has no bearing.[15], [16] Since this emerging methodology is as yet hardly understood and has never been applied to natural product chemistry, a late-stage implementation into a route to **1** might help in scouting the strategic assets of this method, as well as any possible pitfalls.

Finally, a literature survey showed that the amounts of brefeldin A (**1**) formed de novo in the numerous campaigns of the last four decades were minute and mostly in the single-digit milligram range, where documented.[6], [17], [18] Although **1** is accessible by fermentation,[19] this status quo is deemed inadequate by today’s standards in the field of target-oriented synthesis.[20] Therefore we felt encouraged to pursue this prominent target once again, hoping that a new route based on alkyne *trans*-hydrogenation would lead to a more satisfactory solution.

The *meso*-diester **3** served as a convenient point of departure and was desymmetrized on a large scale through a semi-hydrolysis catalyzed by pig liver esterase (Scheme 1).[21], [22] The resulting mono-acid **4**, which is also commercially available, was converted into lactone **5** (*ee*=96 %) prior to oxidative cleavage of the double bond.[22] An intramolecular Claisen condensation/decarboxylation sequence transformed the tricarbonyl compound **6** into product **7** in one operation.[11c] Although the yield was somewhat scale-dependent, multigram amounts of ketone **7** were procured upon slight modification of the literature procedure. Its annulated bicyclic skeleton renders the catalytic hydrogenation of the carbonyl group over platinum on charcoal rigorously stereoselective;[23] this step was basically quantitative provided that the medium was supplemented with NaOAc to avoid elimination of the nascent hydroxyl group. After TBS protection, lactone **9** was converted into the methyl-capped alkyne **11** by an iron-catalyzed reductive alkylation recently developed in our laboratory.[24] To this end, **9** was reacted with PPh_3_/CCl_4_ and the resulting dichloroolefin **10** treated with MeLi in the presence of catalytic amounts of [Fe(acac)_3_] and *ortho*-phenylenediamine to furnish the desired product **11** in 55 % yield on a 3 gram scale (single largest batch). Not unexpectedly, intermediate **10** is sensitive and should be used without delay.[24] Moreover, inadvertent cleavage of the TBS-ether led to spontaneous addition of the alcohol across the activated dichloroalkene bond with formation of the stable tricyclic cage structure **12** (Figure 1).[23] Upon consideration of these peculiarities, however, the iron-catalyzed alkyne formation nicely secured a substantial material supply.

**Figure 1:**
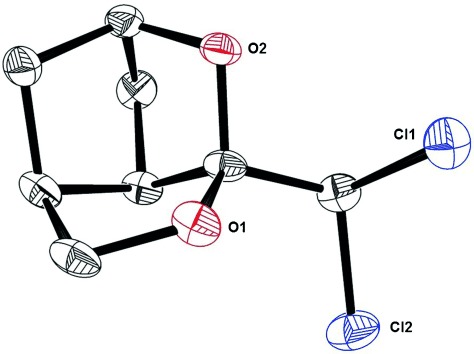
Structure of adduct 12 in the solid state.

**Scheme 1:**
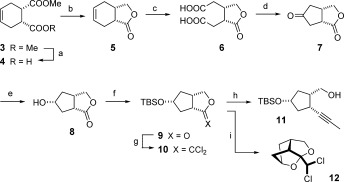
Reagents and conditions (the scales refer to the single largest batches; where a second entry is given, it refers to the batch with the highest yield): a) pig liver esterase, aq. phosphate buffer, pH 7.1, 94 % (75 g scale); b) LiBHEt_3_, THF, 0 °C→RT, then HCl, 98 % (*ee*=96 %, 28 g scale); c) KMnO_4_, aq. acetone, 0 °C→RT, 71 % (27 g scale) *or* 97 % (280 mg scale); d) i) Ac_2_O, 130 °C; ii) K_2_CO_3_, THF, 60 °C, 73 % (1 g scale) *or* 56 % (10 g scale); e) H_2_ (1 atm), Pt/C (1.4 mol % Pt), EtOAc, NaOAc, 99 % (4 g scale); f) TBSOTf, 2,6-lutidine, CH_2_Cl_2_, 0 °C, 98 % (7 g scale); g) CCl_4_, PPh_3_, THF, reflux; h) MeLi, Fe(acac)_3_ (10 mol %), 1,2-phenylenediamine (25 mol %), Et_2_O, 0 °C, 55 % (over both steps, 3 g scale); i) ultrasonication of crude 10, CH_2_Cl_2_, 64 % (4.6 g scale); acac=acetylacetonate; TBS=*tert*-butyldimethylsilyl; Tf=trifluoromethanesulfonyl.

Next, compound **11** was oxidized and the resulting aldehyde isomerized to the thermodynamically more stable *trans*-configured product **13** on exposure to K_2_CO_3_ in MeOH (Scheme 2). The following addition of the readily prepared propiolate **16** required careful optimization. Of the various procedures investigated, the method developed by Kojima and co-workers was the most practical in that only 1.5 equivalents of **16** were needed to reach full conversion of **13**.[25] For high diastereoselectivity however, the steering aminoalcohol **24** described in the literature had to be supplanted by the more bulky analogue **25**, which furnished the desired adduct **17** with a d.r. of >95:5. Subsequent reduction with Red-Al at low temperature[26] followed by TBS protection of the resulting allylic alcohol gave diyne **18** in readiness for macrocyclization.

**Scheme 2:**
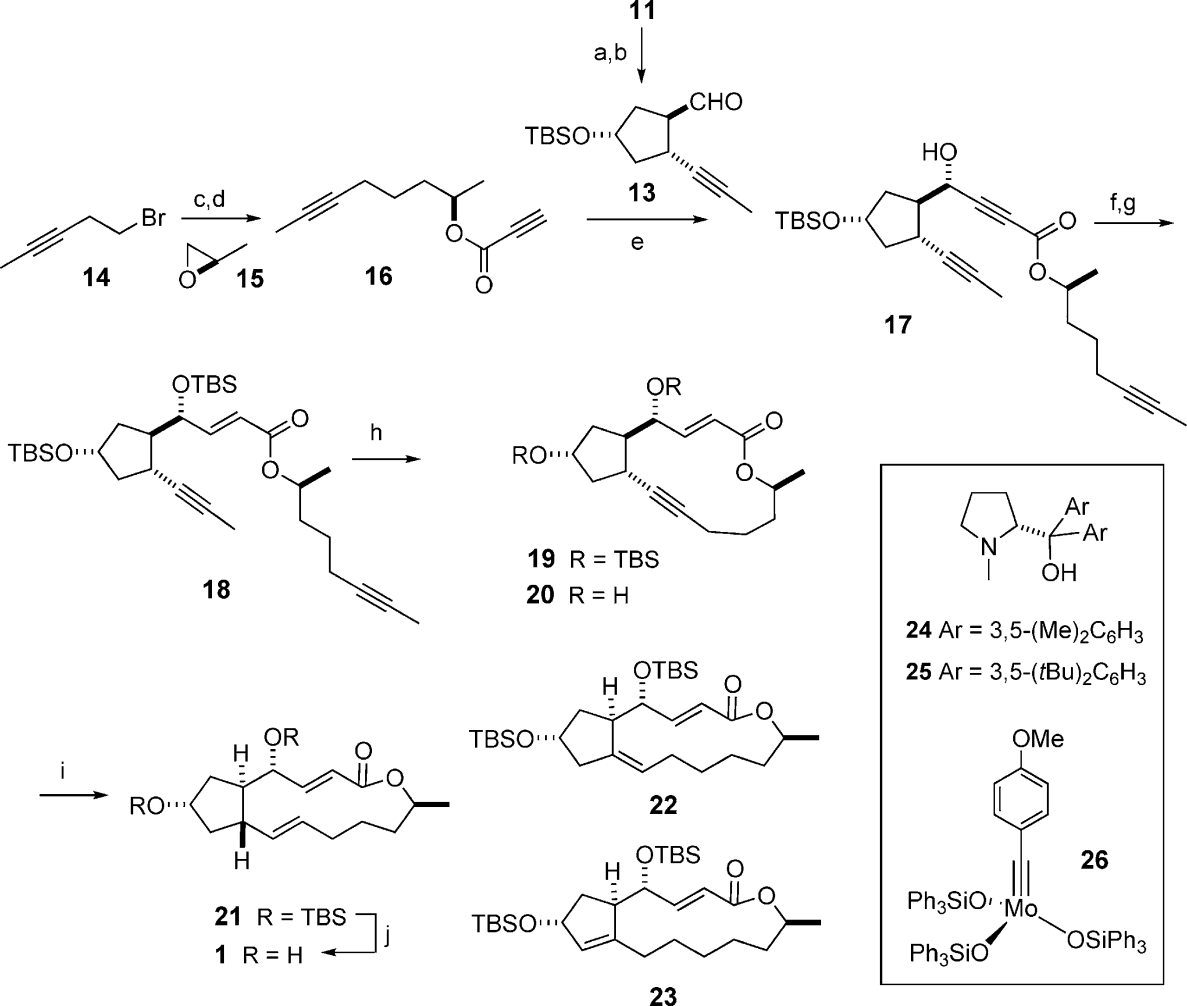
Reagents and conditions (the scales refer to the single largest batches): a) Dess–Martin periodinane, pyridine, CH_2_Cl_2_, 89 % (2 g scale); b) K_2_CO_3_, MeOH, 88 % (2 g scale); c) i) activated Mg, THF, 0 °C; ii) CuCN (10 mol %), 15, −78 °C, 88 % (3.6 g scale); d) propiolic acid, DIAD, PPh_3_, THF, 0 °C, 66 % (1.2 g scale); e) Me_2_Zn, 25 (27 mol %), toluene, 66 % (2 g scale, d.r.>95:5); f) Red-Al, THF, −78 °C, 93 % (2 g scale); g) TBSOTf, pyridine, CH_2_Cl_2_, 0 °C, 93 % (1.3 g scale); h) 26 (5 mol %), toluene, MS 5 Å, 80 °C, 67 % (1.25 g scale); i) H_2_ (30 atm), [Cp*Ru(MeCN)_3_]PF_6_ (5 mol %), CH_2_Cl_2_, 21 (56 %, 1.15 g scale)+22 23 (ca. 20 %); j) aq. HCl, THF, 94 %; Cp*=pentamethylcyclopentadienyl; DIAD=di-isopropyl azodicarboxylate; MS=molecular sieves; Red-Al=sodium bis(2-methoxyethoxy)-aluminum hydride.

This transformation was accomplished with the aid of the molybdenum alkylidyne complex **26**,[27] which is arguably the most active and selective catalyst for alkyne metathesis known to date.[28] Although this catalyst is, a priori, operative at ambient temperature, the formation of **19** required gentle heating, which is thought to reflect the strained nature of the incipient cycloalkyne.[29], [30] With this proviso, the macrocyclization proceeded well on a 1.25 g scale (single largest batch).

The ^1^H NMR spectrum of **19** (CDCl_3_) is distinguished by a remarkable downfield shift of the enoate proton H3 (*δ*=7.28 ppm), which is attributed to deshielding by the anisotropy cone of the acetylene unit. If this is the case, the compound must adopt a rigid conformation that holds the two π-systems in close transannular proximity. The structure of the derived diol **20** in the solid state confirmed this interpretation (Figure 2):[23] it shows H3 to be inward oriented, directed towards the triple bond, and positioned slightly below the plane of the macrocyclic scaffold; tight contacts with C10/C11 indicate significant transannular strain.

**Figure 2:**
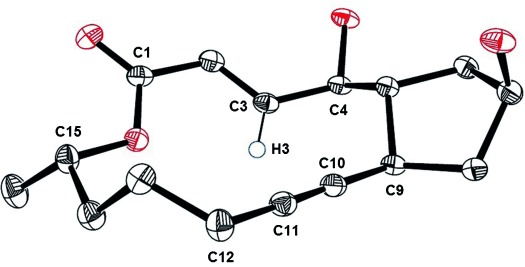
Structure of the cycloalkyne 20 in the solid state; the short distances H3⋅⋅⋅C10 (2.638 Å) and H3⋅⋅⋅C11 (3.106 Å) indicate substantial transannular contacts.

With an appreciable amount of cycloalkyne **19** in hand, the stage was set for the crucial *trans*-hydrogenation. Our model studies had identified [Cp*Ru(cod)Cl] as a good catalyst for this unorthodox transformation.[15] In fact, this complex furnished *E*-**21** with excellent selectivity (*E*:*Z*>95:5) but resulted in substantial overreduction (≤40 %). Although this outcome remains unexplained at this point, we have reason to believe that the strained nature of this particular substrate and the presence of a second coordination site for the active ruthenium center in close transannular proximity to the triple bond render the reduction of **19** particularly challenging.[31] Gratifyingly though, overreduction became a very minor issue (<5 %) when [Cp*Ru(MeCN)_3_]PF_6_ was used instead, which furnished *E*-**21** in stereochemically pure form (*E*:*Z*>99:1). The equally reducible enoate moiety was not touched to any noticeable extent nor was the lactone cleaved by the Lewis-acidic catalyst species generated in situ; neither functional group would subsist under Birch conditions.[32] However, some isomerization of the newly formed disubstituted double bond in **21** into a thermodynamically more favorable trisubstituted position at the ring junction (**22**) or within the five-membered ring (**23**) could not be suppressed. Although the presence of these isomers rendered product isolation more demanding, geometrically and positionally pure **21** was secured in appreciable 56 % yield when the reaction was performed on a >1 g scale. Standard deprotection then furnished brefeldin A (**1**) as a colorless crystalline material. Its integrity and identity were confirmed by spectroscopic means as well as X-ray diffraction.[23]

The new route to brefeldin A (**1**) outlined above is no more than par with the shortest previous syntheses of this target in terms of step count.[6] However, it is deemed competitive and arguably highly practical and therefore constitutes a potentially relevant entry to the debate about synthetic efficiency in general. Most notably, all critical steps are under rigorous catalyst control. Likewise, the great share of catalysis was instrumental for the ready adaptation to the (multi)gram scale; thus, substantially more material was prepared than in any of the numerous campaigns described in the literature (for which the throughput has been properly documented). To this end, it was essential that catalytic ring-closing alkyne metathesis once more proved itself a convincing alternative to the previously executed macrocyclization reactions, be they based on C—C bond formation or traditional lactonization. Finally, the first late-stage implementation of a direct alkyne *trans*-hydrogenation illustrates another recent advance in catalysis that allows chemoselectivity problems, for which the established stoichiometric repertoire has no adequate answer, to be solved. At the same time, however, the present case also shows that a better understanding of this still enigmatic process is necessary to avoid issues with possible alkene isomerization and overreduction. Work along these lines is ongoing in our laboratory.
