# Supplementary material for: trans-Hydrogenation: Application to a Concise and Scalable Synthesis of Brefeldin A
Source: Angew Chem Int Ed Engl. 2015 Feb 4;54(13):3978–82. doi: 10.1002/anie.201411618 (PMC4471581; doi:10.1002/anie.201411618)
Supplement: Supplementary file 1 [file anie0054-3978-sd1.pdf]

Angewandte Chemie
Eine Zeitschrift der Gesellschaft Deutscher Chemiker

Supporting Information

trans-Hydrogenation: Application to a Concise and Scalable Synthesis of Brefeldin A**

*Michael Fuchs and Alois Fürstner**

anie_201411618_sm_miscellaneous_information.pdf

TOTAL SYNTHESES OF BREFELDIN A

Autor (*)	Year[a]	Ref.	shown substrate[b]	"real" substrate[c]	macrocyclization		Steps (Σ)[d]	Amount[e]	Comments[f]
					method	yield (%)			
Corey	1976	1	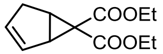	cyclopentadiene	lactonization	nr	≈ 22	nr	racemic
Bartlett	1978	2	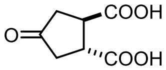	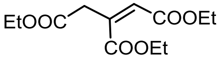	lactonization	37	≈ 17	1 mg	racemic<br>**Birch-reduction ($\Delta^{10,11}$)**
Kitahara, Mori	1979	3	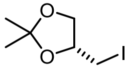	mannitol & glutamic acid	lactonization	42	≈ 31	2.9 mg	**Birch-reduction ($\Delta^{10,11}$)**
Greene	1980	4,5	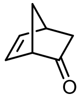	cyclopentadiene	lactonization	70[h]	≈18	16.1 mg	
Winterfeldt	1980	6	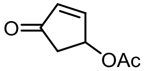	cyclopentadiene (?)	lactonization	nr	> 15	nr	racemic
Yamaguchi	1981	7	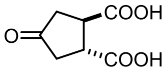	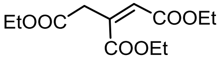	lactonization	94[h]	≈ 21	nr	racemic
Gais	1984	8	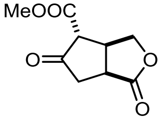	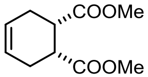	lactonization	74	≈ 24	nr	**Birch-reduction ($\Delta^{10,11}$)**
Corey	1990	9	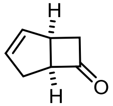	cyclopentadiene	lactonization [g]	nr	≈ 17	nr	
Takano	1990	10	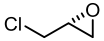		lactonization	83	≈ 25	nr	**Birch-reduction ($\Delta^{10,11}$)**
Taber	1991	11	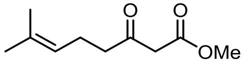		lactonization	21[i]	≈ 23	1.1 mg	
Nokami	1991	12	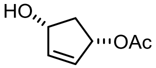	cyclopentadiene	lactonization	80	≈ 16	nr	
Solladié	1993	13	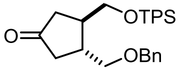	bis-(+)-menthyl fumarate	lactonization	50	≈ 38	4.2 mg	
Kajiwara	1994	14	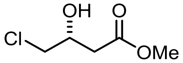		lactonization	24[i]	≈ 22	nr	**Birch-reduction ($\Delta^{10,11}$)**

Roberts	1994	15	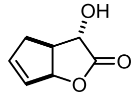	cyclopentadiene	lactonization	80	≈ 14	5.2 mg	
Haynes	1997	16	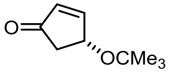	cyclopentadiene (?)	lactonization	78	> 16	5.9 mg	
Suh	1998	17	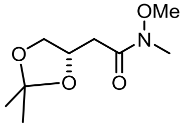	malic acid	lactonization	51	≈ 18	2.2 mg	
Romo	2002	18	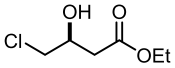		HWE @ $\Delta^{2,3}$	41	≈ 15	nr	
Kim	2002	19	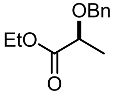	ethyl lactate	nitrile-oxide cycloadd.	78[h]	≈ 23	14.8 mg	
Trost	2002	20	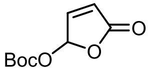	furan	lactonization	61	≈ 18	8.1 mg	***trans*-reduction by hydro-silylation/desilylation** ($\Delta^{2,3}$)
Wu	2004	21	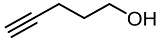		lactonization	81	≈ 16	14 mg	
Suh	2006	22	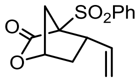	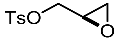	HWE @ $\Delta^{2,3}$	59	≈ 15	nr	
Tae	2009	23	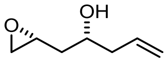	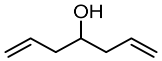	RCM @ $\Delta^{10,11}$	81	≈ 14	4.8 mg	

[a] first appearance; "variants" are considered under one entry if the overall strategy is unchanged
[b] substrate with which the sequence described in the cited reference starts
[c] compound from which the substrate shown in the publication has been made according to the cited literature; not in all cases this may be the actual point of departure; (?) indicates cases, where the real starting material is not clear (best guess by the present authors)
[d] the step count is not necessarily unambiguous
[e] amount of brefeldin A shown in the Experimental Part of the publication
[f] strategic elements related to the present synthesis are indicated in blue
[g] also reports an attempted but unsuccessful macrocyclization via intramolecular 1,4-addition
[h] yield of a mixture of diastereomers
[i] yield over more than one step; the yield of the macrocyclization itself is not specified

nr = not reported; HWE = Horner-Wadsworth-Emmons olefination; RCM = ring closing alkene metathesis

FORMAL TOTAL SYNTHESES OF BREFELDIN A

Autor (*)	Year[a]	Ref.	shown Substrate[b]	“real” substrate[c]	Steps (Σ) [d]	Comments
Ohrui	1980	24	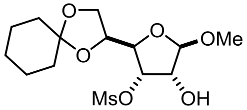	glucose	>> 20	intercepts ref. 7
Winterfeldt	1981	25	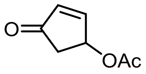	cyclopentadiene (?)		racemic, HWE @ $\Delta^{2,3}$ (yield not reported) intercepts ref. 6
Greene	1982	5	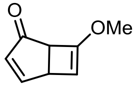	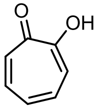	>> 13	racemic; intercepts ref. 1
Isoe	1985	26	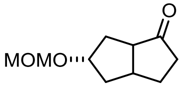	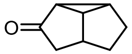	≈ 18	intercepts ref. 2
Sakai	1985	27	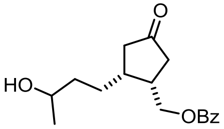	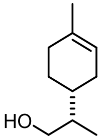	>> 20	intercepts ref. 3
Trost	1986	28	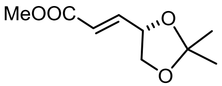	mannitol	≈ 20	intercepts ref. 1
Nakai	1995	29	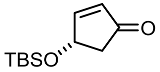		≈ 20	no route established at the time is intercepted
Greene	1995	30	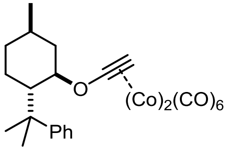	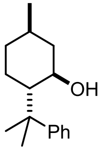	≈ 18	intercepts ref. 5
Kobayashi	1996	31	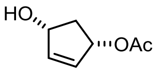	cyclopentadiene (?)	≈ 15	intercepts ref. 2

Mioskowski	1999	32	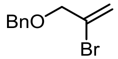	propargyl alcohol (?)	> 20	intercepts ref. 8a
Kim	2002	33	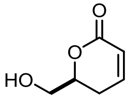	tri-O-acetyl-D-glucal	≈ 27	intramol. nitrile-oxide cycloadd. (84%)<br>**Birch-reduction ($\Delta^{10,11}$)**<br>intercepts ref. 19
Kim	2002	33	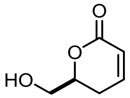	tri-O-acetyl-D-glucal	≈ 22	RCM @ $\Delta^{10,11}$ (42%, E:Z = 2.2:1); intercepts ref. 19
Helmchen	2006	34	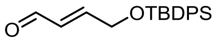	2-buten-1,4-diol	≈ 19	intercepts ref. 11
Zercher	2007	35	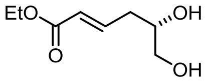	malic acid	≈ 17	RCM @ $\Delta^{10,11}$ (64%, E:Z = 3.5:1) & ring expansion<br>intercepts ref. 19
other		36				

[a] first appearance; “variants” are considered under one entry if the overall strategy is unchanged

[b] substrate with which the sequence described in the cited reference starts

[c] compound from which the substrate shown in the publication has been made according to the cited literature; not in all cases this may be the actual point of departure; (?) indicates cases, where the real starting material is not clear (best guess by the present authors)

[d] projected number of steps towards the final product if the synthesis were completed according to the intercepted route; the step count is not necessarily unambiguous

TOTAL AND FORMAL SYNTHESES OF BREFELDIN C

Autor (*)	Year[a]	Ref.	shown Substrate[b]	“real” substrate[c]	Macrocyclization (yield %)	Steps (Σ) [d]	Amount[e]	Comments[f]
Schreiber	1988	37	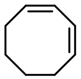		NHK (60%)	≈ 16	≈ 5 mg	
Takano	1989	38	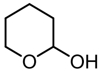		lactonization (85%)	≈ 22	nr	Birch-reduction ($\Delta^{10,11}$)
Guingant	2005	39	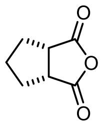	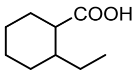	lactonization (79%)	≈ 18	50 mg	
Tsunoda	2011	40	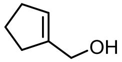	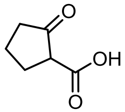	lactonization (89%)	≈ 23	nr	

[a] first appearance; “variants” are considered under one entry if the overall strategy is unchanged
[b] substrate with which the sequence described in the cited reference starts
[c] compound from which the substrate shown in the publication has been made according to the cited literature; not in all cases this may be the actual point of departure; (?) indicates cases, where the real starting material is not clear (best guess by the present authors)
[d] the step count is not necessarily unambiguous
[e] amount of brefeldin A shown in the Experimental Part
[f] strategic elements related to the present synthesis are indicated in blue

nr = not reported; NHK = Nozaki-Hiyama-Kishi reaction

A SELECTION OF SIGNIFICANT ANALOGUES

Autor (*)	Year	Ref.	Structure[a]	Amount
Hori	1997/2000	41	various derivatives	prepared by derivatization of BFA
Cushman	1998	42	various prodrugs	prepared by derivatization of BFA
Helmchen	2006	34	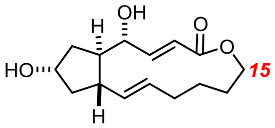	nr
Helmchen	2006	34	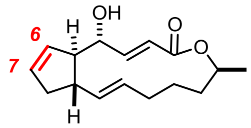	nr
Wu	2008	43	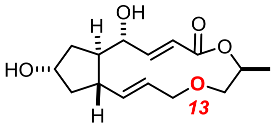	14 mg
Helmchen	2008/2011	44,45	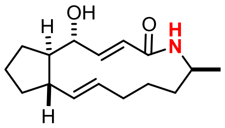	14.3 mg (for total synthesis)<br>125 mg (**via partial synthesis**)
Guingant	2010	39a	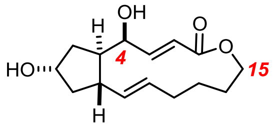	58 mg
Helmchen	2011	45	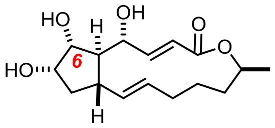	10.8 mg

Helmchen	2011	45	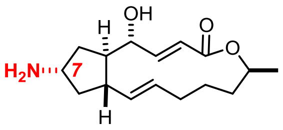	23 mg (**via partial synthesis**)
Helmchen	2013	46	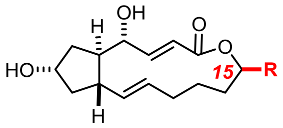<br>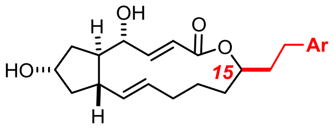	R = Et, 43 mg<br>R = CF_3, 18.8 mg<br>R = vinyl, 78.6 mg<br>Ar = Ph, 42 mg<br>Ar =1-naphthyl, 25.8 mg<br>Ar = 6-quinolinyl, 21.4 mg<br>Ar = 4-dimethylaminophenyl, 18.7 mg<br>Ar = 4-trifluoromethylphenyl, 37.4 mg,<br>Ar = 4-fluoromethyl, 33.4 mg<br>(**all via partial synthesis**)

[a] modified site and/or modification relative to the natural product shown in red

[1] a) E. J. Corey, R. H. Wollenberg, *Tetrahedron Lett*. **1976**, 4705-4708; b) for a modification, see: E. J. Corey, R. H. Wollenberg, D. R. Williams, *Tetrahedron Lett*. **1977**, 2243-2246.

[2] P. A. Bartlett, F. R. Green, *J. Am. Chem. Soc*. **1978**, *100*, 4858-4865.

[3] a) T. Kitahara, K. Mori, M. Matsui, *Tetrahedron Lett*. **1979**, *20*, 3021-3024; b) T. Kitahara, K. Mori, *Tetrahedron* **1984**, *40*, 2935-2944.

[4] A. E. Greene, C. Le Drian, P. Crabbé, *J. Am. Chem. Soc*. **1980**, *102*, 7583-7584.

[5] C. Le Drian, A. E. Greene, *J. Am. Chem. Soc*. **1982**, *104*, 5473-5483.

[6] a) Y. Köksal, P. Raddatz, E. Winterfeldt, *Angew. Chem.* **1980**, *92*, 486-487; *Angew. Chem. Int. Ed. Engl*. **1980**, *19*, 472-473; b) Y. Köksal, P. Raddatz, E. Winterfeldt, *Liebigs Ann. Chem*. **1984**, 450-461.

[7] M. Honda, K. Hirata, H. Sueoka, T. Katsuki, M. Yamaguchi, *Tetrahedron Lett*. **1981**, 22, 2679-2682.

[8] a) H.-J. Gais, T. Lied, *Angew. Chem*. **1984**, *96*, 143-145; *Angew. Chem. Int. Ed. Engl.* **1984**, *23*, 145-146; b) H.-J. Gais, K. L. Lukas, *Angew. Chem*. **1984**, *96*, 140-141; *Angew. Chem. Int. Ed. Engl.* **1984**, *23*, 142-143.

[9] E. J. Corey, P. Carpino, *Tetrahedron Lett*. **1990**, *31*, 7555-7558.

[10] S. Hatakeyama, K. Sugawara, M. Kawamura, S. Takano, *Synlett* **1990**, 691-693.

[11] D. F. Taber, L. J. Silverberg, E. D. Robinson, *J. Am. Chem. Soc*. **1991**, *113*, 6639-6645.

[12] J. Nokami, M. Ohkura, Y. Dan-Oh, Y. Sakamoto, *Tetrahedron Lett*. **1991**, *32*, 2409-2412.

[13] G. Solladié, O. Lohse, *J. Org. Chem*. **1993**, *58*, 4555-4563.

[14] H. Miyaoka, M. Kajiwara, *J. Chem. Soc. Chem. Commun*. **1994**, 483-484.

[15] a) G. Casy, G. Gorins, R. McCague, H. F. Olivo, S. M. Roberts, *J. Chem. Soc. Chem. Commun*. **1994**, 1085-1086; b) A. J. Carnell, G. Casy, G. Gorins, A. Kompany-Saeid, R. McCague, H. F. Olivo, S. M. Roberts, A. J. Willetts, *J. Chem. Soc. Perkin Trans. I* **1994**, 3431-3439.

[16] R. K. Haynes, W. W.-L Lam, L.-L. Yeung, I. D. Williams, A. C. Ridley, S. M. Starling, S. C. Vonwiller, T. W. Hambley, P. Lelandais, *J. Org. Chem*. **1997**, *62*, 4552-4553.

[17] a) Y.-G. Suh, J.-K. Jung, B.-C. Suh, Y.-C. Lee, S.-A. Kim, *Tetrahedron Lett*. **1998**, *39*, 5377-5380; b) Y.-G. Suh, J.-K. Jung, S.-Y. Seo, K.-H. Min, D.-Y. Shin, Y.-S. Lee, S.-H. Kim, H.-J. Park, *J. Org. Chem*. **2002**, *67*, 4127-4137; c) Y.-G. Suh, S.-Y. Seo, J.-K. Jung, O.-H. Park, R.-O. Jeon, *Tetrahedron Lett*. **2001**, *42*, 1691-1694.

[18] Y. Wang, D. Romo, *Org. Lett*. **2002**, *4*, 3231-3234.

[19] D. Kim, J. Lee, P. J. Shim, J. I. Lim, H. Jo, S. Kim, *J. Org. Chem*. **2002**, *67*, 764-771.

[20] a) B. M. Trost, M. L. Crawley, *J. Am. Chem. Soc*. **2002**, *124*, 9328-9329; b) B. M. Trost, M. L. Crawley, *Chem. Eur. J*. **2004**, *10*, 2237-2252.

[21] a) Y. Wu, J. Gao, *Org. Lett*. **2008**, *10*, 1533-1536; for a variant, see: b) Y. Wu, X. Shen, Y.-Q. Yang, Q. Hu, J.-H. Huang, *J. Org. Chem*. **2004**, *69*, 3857-3865; c) Y. Wu, X. Shen, Y.-Q. Yang, Q. Hu, J.-H. Huang, *Tetrahedron Lett*. **2004**, *45*, 199-202.

[22] S.-Y. Seo, J.-K. Jung, S.-M. Paek, Y.-S. Lee, S.-H. Kim, Y.-G. Suh, *Tetrahedron Lett*. **2006**, *47*, 6527-6539.

[23] M.-Y. Kim, H. Kim, J. Tae, *Synlett* **2009**, 1303-1306.

[24] H. Ohrui, H. Kuzuhara, *Agric. Biol. Chem*. **1980**, *44*, 907-912.

[25] P. Raddatz, E. Winterfeldt, *Angew. Chem.* **1981**, *93*, 281-282; *Angew. Chem. Int. Ed. Engl*. **1981**, *20*, 286-287.

[26] K. Nakatani, S. Isoe, *Tetrahedron Lett*. **1985**, *26*, 2209-2212.

[27] K. Ueno, H. Suemune, S. Saeki, K. Sakai, *Chem. Pharm. Bull*. **1985**, *33*, 4021-4025.

[28] B. M. Trost, J. Lynch, P. Renaut, D. H. Steinman, *J. Am. Chem. Soc*. **1986**, *108*, 284-291.

[29] K. Tomooka, K. Ishikawa, T. Nakai, *Synlett* **1995**, 901-902.

[30] V. Bernardes, N. Kann, A. Riera, A. Moyano, M. A. Pericas, A. E. Greene, *J. Org. Chem*. **1995**, *60*, 6670-6671.

[31] Y. Kobayashi, K. Watatani, Y. Kikori, R. Mizojiri, *Tetrahedron Lett*. **1996**, *37*, 6125-6128.

[32] P. Ducray, B. Rousseau, C. Mioskowski, *J. Org. Chem*. **1999**, *64*, 3800-3801.

[33] D. Kim, J. Lee, P. J. Shim, J. I. Lim, T. Doi, S. Kim, *J. Org. Chem*. **2002**, *67*, 772-781.

[34] T. Hübscher, G. Helmchen, *Synlett* **2006**, 1323-1326.

[35] W. Lin, C. K. Zercher, *J. Org. Chem*. **2007**, *72*, 4390-4395.

[36] For other studies towards the brefeldins, see: a) D. B. Ramachary, Y. V. Reddy, *J. Org. Chem*. **2010**, 75, 74-85; b) D. P. Curran, D. Scholz, *Monatsh. Chem*. **1977**, *108*, 1401-1411; c) T. Livinghouse, R. V. Stevens, *J. Chem. Soc. Chem. Commun.* **1978**, 754-756; d) R. Baker, R. B. Keen, M. D. Morris, R. W. Turner, *J. Chem. Soc. Chem. Commun*. **1984**, 987-988; e) K. Nakamura, T. Kitayama, Y. Inoue, A. Ohno, *Bull. Chem. Soc. Jpn*. **1990**, *63*, 91-96.

[37] S. L. Schreiber, H. V. Meyers, *J. Am. Chem. Soc*. **1988**, *110*, 5198-5200.

[38] S. Hatakeyama, K. Osanai, H. Numata, S. Takano, *Tetrahedron Lett*. **1989**, *30*, 4845-4848.

[39] a) S. Archambaud, F. Legrand, K. Aphecetche-Julienne, S. Collet, A. Guingant, M. Evain, *Eur. J. Org. Chem*. **2010**, 1364-1380; b) S. Archambaud, K. Aphecetche-Julienne, A. Guingant, *Synlett* **2005**, 139-143; c) F. Legrand, S. Archambaud, S. Collet, K. Aphecetche-Julienne, A. Guingant, M. Evain, *Synlett* **2008**, 389-393.

[40] M. Inai, T. Nishii, S. Mukoujima, T. Esumi, H. Kaku, K. Tominaga, H. Abe, M. Horikawa, T. Tsunoda, *Synlett* **2011**, 1459-1461.

[41] a) J.-W. Zhu, H. Nagasawa, F. Nagura, S. B. Mohamad, Y. Uto, K. Ohkura, H. Hori, *Bioorg. Med. Chem*. **2000**, *8*, 455-463; b) J.-W. Zhu, H. Hori, H. Nojiri, T. Tsukuda, Z. Taira, *Bioorg. Med. Chem. Lett*. **1997**, *7*, 139-144.

[42] A. B. Argade, R. Devraj, J. A. Vroman, R. D. Haugwitz, M. Hollingshead, M. Cushman, *J. Med. Chem.* **1998**, *41*, 3337-3346.

[43] J. Gao, Y.-X. Huang, Y. Wu, *Tetrahedron* **2008**, *64*, 11105-11109.

[44] S. Förster, G. Helmchen, *Synlett* **2008**, 831-836.

[45] S. Förster, E. Persch, O. Tverskoy, F. Rominger, G. Helmchen, C. Klein, B. Gönen, B. Brügger, *Eur. J. Org. Chem*. **2011**, 878-891.

[46] K. Seehafer, F. Rominger, G. Helmchen, M. Langhans, D. G. Robinson, B. Özata, B. Brügger, J. R. P. M. Strating, F. J. M. van Kuppeveld, C. D. Klein, *J. Med. Chem*. **2013**, *56*, 5872-5884.

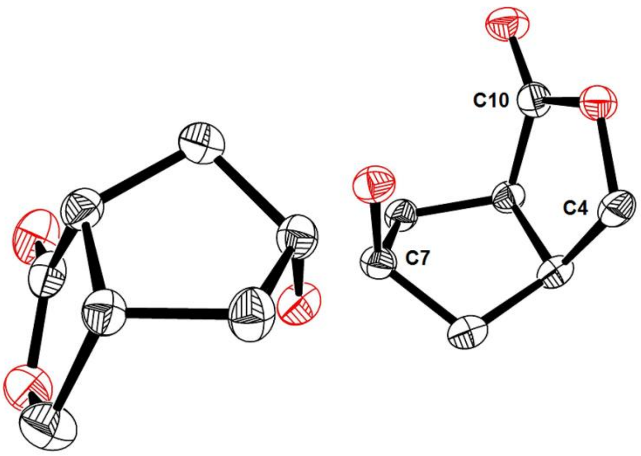

Figure S-1. Structure of compound **8** in the solid state; anisotropic displacement parameters are drawn at the 50% probability level, hydrogen atoms are omitted for clarity; brefeldin numbering scheme **(CCDC-1036054**)

X-ray Crystal Structure Analysis of Compound 8: $C_7\,H_{10}\,O_3$, M_r = 142.15 g · mol^{-1}, colorless plate, crystal size 0.32 x 0.19 x 0.12 mm, monoclinic, space group $P2_1$, a = 5.3098(4) Å, b = 10.2790(7) Å, c = 12.3060(9) Å, β = 97.493(4)°, V = 665.92(8) Å^3, T = 100 K, Z = 4, D_{calc} = 1.418 g · cm^3, λ = 1.54178 Å, $\mu(Cu\text{-}K_\alpha)$ = 0.930 mm^{-1}, Empirical absorption correction (T_{min} = 0.77, T_{max} = 0.90), Bruker AXS X8 Proteum diffractometer, 3.623 < θ < 66.290°, 14753 measured reflections, 2300 independent reflections, 2182 reflections with $I > 2\sigma(I)$, Structure solved by direct methods and refined by full-matrix least-squares against F^2 to R_1 = 0.030 [$I > 2\sigma(I)$], wR_2 = 0.078, 183 parameters, H atoms riding, absolute structure parameter = 0.0(2), S = 1.040, residual electron density 0.2 / -0.2 e Å^{-3}.

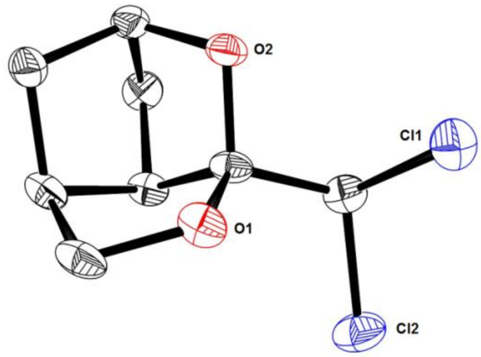

Figure S-2. Structure of adduct **12** in the solid state; anisotropic displacement parameters are drawn at the 50% probability level, hydrogen atoms are omitted for clarity (**CCDC-1036055**)

X-ray Crystal Structure Analysis of Compound 12: $C_8\,H_{10}\,Cl_2\,O_2$, M_r = 209.06 g · mol^{-1}, colorless plate, crystal size 0.21 x 0.20 x 0.17 mm, orthorhombic, space group $P2_12_12_1$, a = 8.0685(5) Å, b = 9.3727(5) Å, c = 11.7206(7) Å, V = 886.35(9) Å^3, T = 100 K, Z = 4, D_{calc} = 1.567 g · cm^3, λ = 1.54178 Å, $\mu(Cu\text{-}K_\alpha)$ = 6.234 mm^{-1}, Empirical absorption correction (T_{min} = 0.34, T_{max} = 0.49), Bruker AXS X8 Proteum

diffractometer, 6.045 < θ < 67.622°, 40841 measured reflections, 1594 independent reflections, 1587 reflections with $I > 2\sigma(I)$, Structure solved by direct methods and refined by full-matrix least-squares against F^2 to R_1 = 0.025 [$I > 2\sigma(I)$], wR_2 = 0.061, 109 parameters, absolute structure parameter = 0.012(5), H atoms riding, S = 1.098, residual electron density 0.1 / -0.3 e $Å^{-3}$.

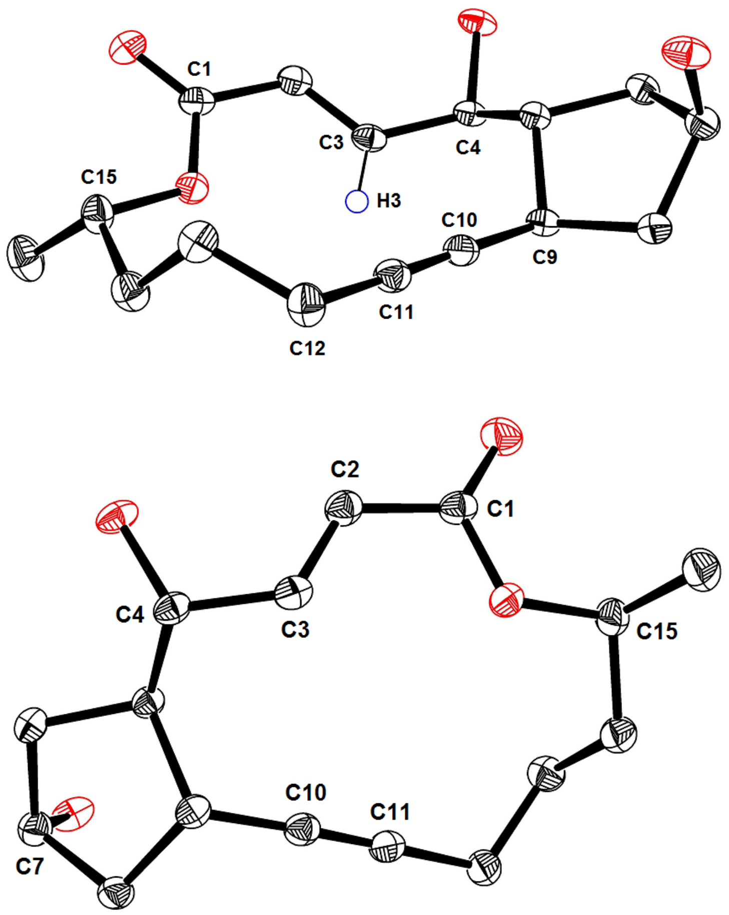

Figure S-3. Structure of cycloalkyne **20** in the solid state in two different orientations; anisotropic displacement parameters are drawn at the 50% probability level; except for H3 shown in the top projection, all hydrogen atoms are omitted for clarity (**CCDC-1036056**)

X-ray Crystal Structure Analysis of Compound 20: $C_{16}H_{22}O_4$, M_r = 278.33 g · mol^{-1}, colorless plate, crystal size 0.21 x 0.11 x 0.07 mm, orthorhombic, space group $P2_12_12_1$, a = 7.4004(2) Å, b = 10.6316(3) Å, c = 18.5415(5) Å, V = 1458.81(7) $Å^3$, T = 100 K, Z = 4, D_{calc} = 1.267 g·cm^3, λ = 1.54178 Å, $\mu(Cu\text{-}K_\alpha)$ = 0.732 mm^{-1}, Semi-empirical absorption correction (T_{min} = 0.87, T_{max} = 0.95), Bruker AXS X8 Proteum diffractometer, 4.770 < θ < 67.815°, 65420 measured reflections, 2618 independent reflections, 2541 reflections with $I > 2\sigma(I)$, Structure solved by direct methods and refined by full-matrix least-squares against F^2 to R_1 = 0.032 [$I > 2\sigma(I)$], wR_2 = 0.078, 190 parameters, absolute structure parameter = 0.07(6), H atoms riding, S = 1.108, residual electron density 0.1 / -0.2 e $Å^{-3}$.

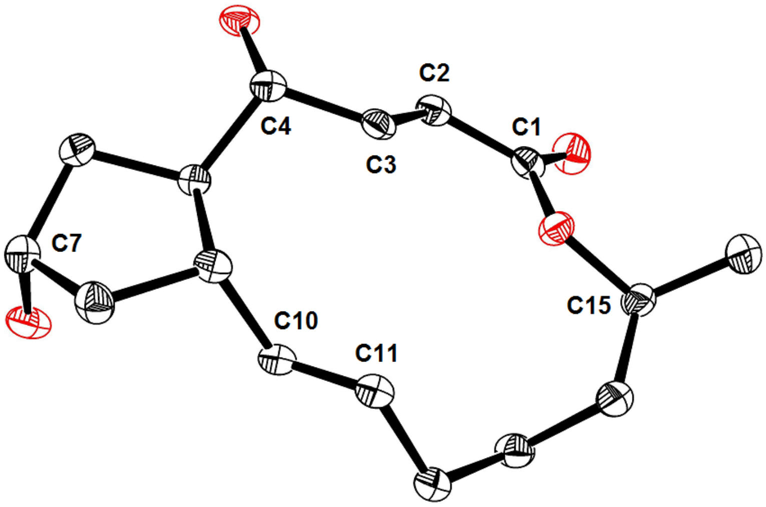

Figure S-4. Structure of brefeldin A (**1**) in the solid state; anisotropic displacement parameters are drawn at the 50% probability level, hydrogen atoms are omitted for clarity (**CCDC- 1036057**)[1]

X-ray Crystal Structure Analysis of 9022: $C_{16} H_{24} O_4$, M_r = 280.35 g · mol^{-1}, colorless plate, crystal size 0.187 x 0.172 x 0.040 mm, orthorhombic, space group $P2_12_12_1$, a = 7.3601(3) Å, b = 10.8657(5) Å, c = 18.7697(9) Å, V = 1501.06(12) Å^3, T = 100 K, Z = 4, D_{calc} = 1.241 g · cm^3, λ = 1.54178 Å, μ(Cu-K_α) = 0.712 mm^{-1}, Empirical absorption correction (T_{min} = 0.90, T_{max} = 0.97), Bruker AXS X8 Proteum diffractometer, 4.702 < θ < 67.536°, 62428 measured reflections, 2704 independent reflections, 2644 reflections with $I > 2\sigma(I)$, Structure solved by direct methods and refined by full-matrix least-squares against F^2 to R_1 = 0.030 [$I > 2\sigma(I)$], wR_2 = 0.072, 198 parameters, absolute structure parameter = 0.00(6), H atoms riding, S = 1.096, residual electron density 0.1 / -0.2 e Å^{-3}.

CCDC-1036054 (**8**), CCDC-1036055 (**12**), CCDC-1036056 (**20**) and CCDC-1036057 (**1**) contain the supporting crystallographic data for this paper. These data can be obtained free of charge from the Cambridge Crystallographic Data Centre via www.ccdc.cam.ac.uk/data_request/cif.

1 For a previous report on the X-ray structure of brefeldin A, see: H. P. Weber, D. Hauser, H. P. Sigg, *Helv. Chim. Acta* **1971**, *54*, 2763-2766.

General. Unless stated otherwise, all reactions were carried out under Ar in flame-dried glassware. The solvents were purified by distillation over the indicated drying agents and were transferred under Ar: THF, Et_2O (Mg/anthracene), CH_2Cl_2, hexane, toluene (Na/K), dioxane, DMF, MeCN , NEt_3 and pyridine were dried by an adsorbtion solvent purification system based on molecular sieves. Thin layer chromatography (TLC): Macherey-Nagel precoated plates (POLYGRAM® SIL/UV254); Flash chromatography: Merck silica gel 60 (40–63 µm) with predistilled or HPLC grade solvents. NMR: Spectra were recorded on Bruker DPX 300, AV400, AV500 or AVIII 600 spectrometer in the solvents indicated; chemical shifts (δ) are given in ppm relative to TMS, coupling constants (*J*) in Hz. The solvent signals were used as references and the chemical shifts converted to the TMS scale ($CDCl_3$: δ_C ≡ 77.16 ppm; residual $CHCl_3$ in $CDCl_3$: δ_H ≡ 7.26 ppm; [D4]-MeOH: δ_C ≡ 49.00 ppm; residual CHD_2OD in [D4]-methanol: δ_H ≡ 3.31 ppm; [D6]-DMSO: δ_C ≡ 39.52 ppm; residual [D5]-DMSO: δ_H ≡ 2.50 ppm); IR: Spectrum One (Perkin-Elmer) spectrometer, wavenumbers (ῦ) in cm^{-1}; MS (EI): Finnigan MAT 8200 (70 eV), ESI-MS: ESQ 3000 (Bruker), accurate mass determinations: Bruker APEX III FT-MS (7 T magnet) or Mat 95 (Finnigan). Optical rotations ($[\alpha]_D^{20}$) were measured with a Perkin-Elmer Model 343 polarimeter. Unless stated otherwise, all commercially available compounds (Alfa Aesar, Aldrich, Fluka, Lancaster) were used as received. Complex **26**[1] and $[Cp^*Ru(MeCN)_3]PF_6$[2] were prepared according to literature procedures.

(1*R*,2*S*)-Diethyl cyclohex-4-ene-1,2-dicarboxylate (3).[3] H_2SO_4 (conc., 25 mL, 469 mmol) was added to a solution of *cis*-1,2,3,6-tetrahydrophthalic anhydride (60.0 g, 394 mmol) in MeOH (600 mL) and the resulting mixture was stirred overnight at reflux temperature. The mixture was then concentrated under reduced pressure and the remaining oil diluted with water (100 mL). Solid $NaHCO_3$ was carefully added until the pH was neutral. The aqueous phase was extracted with *tert*-butyl methyl ether (4 x 100 mL), and the combined organic phases were dried over Na_2SO_4, filtered and concentrated to give product **3** as a clear oil (75.0 g, 96%). ^{1}H NMR (400 MHz, $CDCl_3$): δ = 5.65 (s, 2H), 3.67 (s, 6H), 3.02 (t, *J* = 5.3, 2H), 2.53 (dd, *J* = 5.3, 16.2 Hz, 2H), 2.32 (dd, *J* = 5.3, 16.2 Hz, 2H); ^{13}C NMR (100 MHz, $CDCl_3$): δ = 173.8, 125.2, 51.9, 39.8, 25.8; IR (film) ῦ = 3029, 2952, 2848, 1729, 1435, 1200, 1163, 1025, 660; MS (EI): *m/z*: 198 (0.25), 167 (14), 138 (35), 107 (9), 91 (1), 79 (100), 59 (9); HRMS (ESI): *m/z*: calc. for $C_{10}H_{14}O_4Na$: 221.0784 $[M+Na]^+$, found: 221.0785.

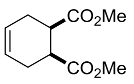

(1*R*,6*S*)-6-(Methoxycarbonyl)cyclohex-3-enecarboxylic acid (4).[4] Diester **3** (74.5 g, 376 mmol) was suspended in phosphate buffer (1340 mL, 100 mM, pH = 7.0). Pig liver esterase (10.9 kU, 728 mg lyophilized powder) and ammonium sulfate (3 M in water, 3.16 mL) were added and the pH was kept constant by addition of NaOH (1 M) *via* a pH-stat for 2 d. For work up, the pH was adjusted to ≈ 10 by the addition of NaOH (1 M) and the obtained slurry was extracted with *tert*-butyl methyl ether (2 x 500 mL). The aqueous phase was acidified with conc. HCl until a pH 1 was reached, which led to significant precipitation of the enzyme. To facilitate the extraction, *tert*-butyl methyl ether (500 mL) was added and the mixture was filtered through a pad of Celite, which was carefully washed with water (100 mL) and *tert*-butyl methyl ether (100 mL). The

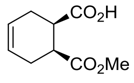

phases were separated and the aqueous phase was extracted with *tert*-butyl methyl ether (2 x 500 mL). The combined organic layers of the second extraction step (under acidic conditions) were dried over Na_2SO_4, filtered and concentrated to give compound **4** as a pale yellow oil (65.2 g, 94%). $[\alpha]_D^{20}$ = +17.7 [c = 1.0, EtOH, lit.:[5] +17.7 (c = 1.0, EtOH)]; ^{1}H NMR (400 MHz, $CDCl_3$): δ = 5.67 (s, 2H), 3.69 (s, 3H), 3.10-3.03 (m, 2H), 2.61-2.54 (m, 2H), 2.40-2.33 (m, 2H); ^{13}C NMR (100 MHz, $CDCl_3$): δ = 179.8, 173.8, 125.3, 125.2, 52.1, 39.7, 39.6, 25.9, 25.7; IR (film) ῡ = 3100br, 3031, 2952, 2851, 1731, 1704, 1655, 1436, 1297, 1264, 1203, 1163, 1033, 736, 663; MS (EI): *m/z*: 184 (1), 166 (8), 153 (11), 138 (24), 124 (27), 107 (7), 97 (4), 79 (100); HRMS (ESI): *m/z*: calc. for $C_9H_{11}O_4Na_2$: 229.0447 $[M+2Na]^+$, found: 229.0448.

(3a*S*,7a*R*)-3a,4,7,7a-Tetrahydroisobenzofuran-1(3*H*)-one (5).[6] A flame-dried round bottom flask

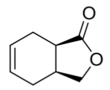

equipped with a droping funnel was charged with $LiEt_3BH$ (1 M in THF, 608 mL, 608 mmol) under argon. The solution was cooled to 0 °C before a solution of compound **4** (28.0 g, 152 mmol) in THF (20 mL) was added over a period of 30 min. Once the addition was complete, the mixture was stirred for 1 h at 0 °C and for 3 h at room temperature. For work up, the mixture was cooled to 0 °C and the reaction was carefully quenched by addition of aq. HCl (6 M, 500 mL). The resulting mixture was stirred overnight before it was extracted with *tert*-butyl methyl ether (4 x 200 mL). The combined organic layers were dried over Na_2SO_4, filtered and concentrated. The residue was purified by flash chromatography (SiO_2, pentane/*tert*-butyl methyl ether, 3/1) to give the title compound as colorless oil, which was dried under high vacuum for 1 h (20.5 g, 98%). $[\alpha]_D^{20}$ = +49.7 [c = 1.8, EtOAc, lit.:[6] +82.5 (c = 2.0, EtOAc)]; ^{1}H NMR (400 MHz, $CDCl_3$): δ = 5.73 (s, 2H), 4.30 (dd, *J* = 5.2, 8.8 Hz, 1H), 4.01 (dd, *J* = 1.9, 8.8 Hz, 1H), 2.83-2.69 (m, 1H), 2.66-2.57 (m, 1H), 2.52-2.48 (m, 1H), 2.44-2.32 (m, 1H), 2.29-2.23 (m, 1H), 1.93-1.87 (m, 1H); ^{13}C NMR (100 MHz, $CDCl_3$): δ = 179.2, 125.3, 124.9, 72.8, 37.4, 32.1, 24.8, 22.1; IR (film) ῡ = 3031, 2969, 2905, 2843, 1763, 1480, 1435, 1372, 1197, 1173, 1132, 1040, 944, 663; MS (EI): *m/z*: 138 (41), 123 (9), 110 (5), 93 (100), 79 (89); HRMS (ESI): *m/z*: calc. for $C_8H_{10}O_2$: 138.0681 $[M]^+$, found: 138.0681.

2,2'-[(3*R*,4*S*)-2-Oxotetrahydrofuran-3,4-diyl]diacetic acid (6).[6] A solution of lactone **5** (200 mg, 1.45

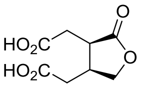

mmol) in acetone (1 mL) and added dropwise over a period of 1 h to a solution of $KMnO_4$ (699 mg, 4.42 mmol) in water (5 mL) at 0 °C. The brown slurry was stirred for 1 h at 0 °C, warmed to room temperature and stirred overnight. $NaHSO_3$ was added in order to destroy any remaining $KMnO_4$. The resulting slurry was filtered through a pad of Celite and the filter cake carefully rinsed with water/THF (1/1, 25 mL). The combined filtrate was acidified to pH 2, saturated with NaCl and extracted with *tert*-butyl methyl ether/THF (2/3, 6 x 40 mL). The combined organic layers were dried over Na_2SO_4, filtered and concentrated under reduced pressure (the bath temperature must not exceed 30 °C). The remaining while solid material was dried under high vacuum and found pure enough for immediate further use (283 mg, 97%).

The reaction was also performed on much larger scale, using $KMnO_4$ (119.4 g, 0.76 mol) in water (650 mL) and lactone **5** (26.1 g, 189 mmol) in acetone (139 mL) to give analytically pure **6** (27.3 g, 71%) which analyzed as follows: m.p. = 162-164 °C (EtOAc, lit.[6] 144-157 °C); $[\alpha]_D^{20}$ = −75.3 [c = 1.0,

MeOH, lit.:[6] –85.3 (c = 3.29, MeOH)]; ^{1}H NMR (400 MHz, [D_6]-DMSO): δ = 12.41 (bs, 2H), 4.38 (dd, *J* = 6.3, 8.9 Hz, 1H), 4.05 (dd, *J* = 2.2, 8.9 Hz, 1H), 3.20 (dd, *J* = 7.9, 14.8 Hz, 1H), 2.94 (bs, 1H), 2.48 (m, 2H), 2.35 (dd, *J* = 4.8, 16.6 Hz, 1H), 2.23 (dd, *J* = 10.1, 16.5 Hz, 1H); ^{13}C NMR (100 MHz, [D_6]-DMSO): δ = 177.5, 172.9, 172.5, 71.0, 38.7, 34.2, 32.5, 30.2; IR (film) ῦ = 2922 (br), 2578 (br), 1752, 1692, 1416, 1385, 1328, 1296, 1222, 1171, 986, 925; MS (EI): *m/z*: 203 (0.34), 184 (10), 166 (6), 156 (24), 143 (49), 125 (41), 112 (100), 97 (47), 85 (36), 70 (40), 67 (42), 55 (64); HRMS (ESI): *m/z*: calc. for $C_8H_{10}O_6Na$: 225.0370 [M+Na]$^+$, found: 225.0370.

(3a*S*,6a*R*)-Tetrahydro-1*H*-cyclopenta[c]furan-1,5(3*H*)-dione (7).[7] Diacid **6** (998 mg, 4.94 mmol) was suspended in Ac_2O (10 mL, 10.8 g, 106 mmol) and the mixture stirred at 130 °C (bath temperature) for 1 h. After cooling to room temperature, the mixture was diluted with THF (15 mL) before K_2CO_3 (667 mg, 4.83 mmol) was added. The resulting mixture was stirred at 60 °C (bath temperature) overnight. After cooling to 0 °C, the reaction was quenched with MeOH (10 mL) and the mixture stirred for 30 min at 0 °C. Sat. aq. NH_4Cl (20 mL) and CH_2Cl_2 (20 mL) were added and stirring continued for 20 min at 0 °C. Phase separation followed by extraction of the aqueous layer with CH_2Cl_2 (3 x 25 mL) gave a combined organic phase, which was dried over Na_2SO_4, filtered and concentrated. The crude product was purified by flash chromatography (SiO_2, hexane/EtOAc, 1/1) to give the title compound as a pale yellow solid (504 mg, 73%).

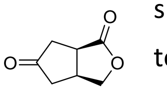

The reaction was also performed on larger scale, using Ac_2O (124 mL, 134 g, 1.31 mol), diacid **6** (10.0 g, 49.5 mmol), K_2CO_3 (6.84 g, 49.5 mmol) and THF (370 mL) to give analytically pure **7** (3.86 g, 56%) which analyzed as follows: m.p. = 81-82 °C (EtOAc, lit.:[4a] 84 °C for *ent*-**6**); $[\alpha]_D^{20}$ = +80.6 [c = 1.1, CH_2Cl_2, lit.:[4a] –67.8 (c = 2.59, CH_2Cl_2, for *ent*-**6**); ^{1}H NMR (400 MHz, $CDCl_3$): δ = 4.53 (dd, *J* = 5.9, 9.6 Hz, 1H), 4.25 (d, *J* = 11.5, 1H), 3.40-3.11 (m, 2H), 2.79-2.51 (m, 3H), 2.21 (dd, *J* = 8.3, 19.1 Hz, 1H); ^{13}C NMR (100 MHz, $CDCl_3$): δ = 214.0, 178.1, 71.8, 42.2, 40.8, 39.3, 36.3; IR (film) ῦ = 2975, 2920, 1765, 1732, 1403, 1371, 1308, 1185, 1173, 1097, 1033, 975; MS (EI): *m/z*: 140 (72), 122 (4), 112 (15), 99 (64), 95 (23), 81 (35), 67 (73), 54 (100); HRMS (ESI): *m/z*: calc. for $C_7H_8O_3$: 140.0473 [M]$^+$, found: 140.0474.

(3a*S*,5*R*,6a*R*)-5-Hydroxyhexahydro-1*H*-cyclopenta[c]furan-1-one (8).[4a] NaOAc (7.04 g, 85.8 mmol) and Pt/C (10 wt-%, 781 mg, 1.4 mol-%) were added to a solution of keto-lactone **7** (4.01 g, 28.6 mmol) in EtOAc (120 mL) and the mixture was stirred under an atmosphere of hydrogen (1 atm) for 1.5 d. The suspension was filtered through a pad of silica which was carefully rinsed with EtOAc (2 x 20 mL). The combined filtrates were evaporated under reduced pressure to give the title compound as pale yellow solid (4.04 g, 99%): m.p. = 64-65°C (EtOAc, lit.:[8] 69-70°C); $[\alpha]_D^{20}$ = –71.0 [c = 2.0, CH_2Cl_2, lit.:[8] –59.5 (c = 1.73, CH_2Cl_2)]; ^{1}H NMR (400 MHz, $CDCl_3$): δ = 4.53 (t, *J* = 8.7, 1H), 4.44 (t, *J* = 3.8, 1H), 4.21 (dd, *J* = 3.1, 9.2 Hz, 1H), 3.16-2.89 (m, 2H), 2.28-2.23 (m, 1H), 2.07-1.90 (m, 2H), 1.88-1.76 (m, 2H); ^{13}C NMR (100 MHz, $CDCl_3$): δ = 181.5, 74.7, 73.6, 43.3, 42.3, 40.4, 36.9; IR (film) ῦ = 3451br, 2939, 1744, 1383, 1265, 1192, 1140, 1105, 1008, 977, 734, 701; MS (EI): *m/z*: 142 (43), 124 (47), 113 (32), 96 (20), 83 (55), 69 (62), 55 (100); HRMS (ESI): *m/z*: calc. for

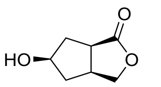

$C_7H_{10}O_3$: 142.0630 [M]$^+$, found: 142.0628. Single crystals suitable for X-ray diffraction were obtained from a solution in EtOAc upon slow evaporation of the solvent.

(3a*S*,5*R*,6a*R*)-5-[(*tert*-Butyldimethylsilyl)oxy]hexahydro-1H-cyclopenta[c]furan-1-one (9). TBSOTf

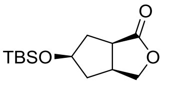

(9.78 mL, 11.3 g, 42.6 mmol) was added dropwise at 0 °C to a solution of 2,6-lutidine (6.6 mL, 6.09 g, 56.8 mmol) and hydroxylactone **6** (4.04 g, 28.4 mmol) in CH_2Cl_2 (38 mL). After stirring for 1 h at 0 °C, the reaction was quenched by the addition of sat. aq. $NaHCO_3$ (ca. 10 mL) and the aqueous layer was extracted with EtOAc (3 x 50 mL). The combined organic phases were washed with aq. $CuSO_4$ (1 M, 5 x 15 mL) and brine (10 mL), before they were dried over Na_2SO_4, filtered and concentrated. The residue was dried under high vacuum to give pure **9** as pale yellow solid (7.12 g, 98%). m.p. = 48-49 °C (EtOAc); $[\alpha]_D^{20} = -20.3$ (c = 1.6, CH_2Cl_2); ^{1}H NMR (400 MHz, $CDCl_3$): δ = 4.51 (t, *J* = 8.7, 1H), 4.34-4.32 (m, 1H), 4.17-4.14 (m, 1H), 3.04-2.99 (m, 2H), 2.20 (d, *J* = 13.5 Hz, 1H), 1.94-1.85 (m, 2H), 1.75 (d, *J* = 13.6 Hz, 1H), 0.85 (s, 9H), 0.04 (s, 6H); ^{13}C NMR (100 MHz, $CDCl_3$): δ = 181.1, 74.9, 74.2, 43.7, 43.5, 40.7, 37.2, 26.0, 18.2, –4.62, –4.65; IR (film) ῦ = 2958, 2928, 2856, 1751, 1377, 1255, 1195, 1045, 1023, 908, 833, 773; MS (EI): *m/z*: 241 (3), 199 (93), 169 (12), 141 (14), 125 (4), 105 (7), 89 (6), 75 (100), 59 (7); HRMS (ESI): *m/z*: calc. for $C_{13}H_{24}O_3SiNa$: 279.1387 [M+Na]$^+$, found: 279.1383.

{(1*S*,2*R*,4*S*)-4-[(*tert*-Butyldimethylsilyl)oxy]-2-(prop-1-yn-1-yl)cyclopentyl]methanol (11). A flame

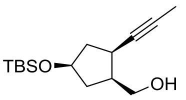

dried 2-neck round bottom flask, equipped with a reflux condenser and a dropping funnel, was charged with PPh_3 (43.4 g, 165 mmol). THF (450 mL) and the lactone **9** (5.31 g, 20.7 mmol) were added and the mixture was stirred at reflux temperature (oil-bath temperature ≈ 80 °C). A solution of CCl_4 (50 mL, 79.5 g, 516 mmol) in THF (50 mL) was added dropwise over a period of 3.5 h. Once the addition was complete, stirring was continued at this temperature for 3 h, before the mixture was cooled and the reaction quenched with water (10 mL). The mixture was extracted with *tert*-butyl methyl ether (3 x 200 mL), the combined organic phases were washed with sat. aq. $NaHCO_3$ (100 mL) and brine (50 mL), before they were dried over Na_2SO_4, filtered and concentrated under reduced pressure. The crude product was purified by flash chromatography (SiO_2, hexane/EtOAc, 50/1) to give the dichloro-olefin **10** (containing minor PPh_3 impurities) which was immediately used for the subsequent reaction. Characteristic data of **10**: ^{1}H NMR (400 MHz, $CDCl_3$): δ = 4.45 (t, *J* = 8.6, 1H), 4.32-4.26 (m, 2H), 3.39 (dt, *J* = 4.6, 9.3 Hz, 1H), 3.21 (s, 1H), 3.02-2.92 (m, 1H), 2.06-2.02 (m, 2H), 1.94-1.88 (m, 1H), 1.72-1.66 (m, 1H), 0.87 (s, 9H), 0.04 (s, 6H); ^{13}C NMR (100 MHz, $CDCl_3$): δ = 159.5, 79.5, 77.4, 74.5, 46.0, 41.4, 41.4, 40.6, 25.8, 18.0, –4.7, –4.9.

A flame-dried Schlenk tube was charged with $Fe(acac)_3$ (896 mg, 2.54 mmol), *ortho*-phenylene-diamine (548 mg, 5.07 mmol), Et_2O (120 mL) and the crude dichloro-olefin **10** from the previous reaction. The solution was cooled to 0 °C before MeLi (1.6 M in Et_2O, 39.6 mL, 63.4 mmol) was slowly added. The mixture was stirred for 10 min at 0 °C and for 2 h at room temperature. For work up, the mixture was cooled to 0 °C before the reaction was carefully quenched with water (20 mL). The aqueous phase was extracted with *tert*-butyl methyl ether (3 x 100 mL) and the combined organic

layers were dried over Na_2SO_4, filtered and concentrated under reduced pressure. The crude product was purified by flash chromatography (SiO_2, hexane/EtOAc, 15/1) to give alkyne **11** as an oil (3.02 g, 55%). $[\alpha]_D^{20} = -2.6$ (c = 2.0, CH_2Cl_2); ^1H NMR (400 MHz, $CDCl_3$): δ = 4.14 (quint, *J* = 6.5, 1H), 3.82 (dd, *J* = 7.6, 11.4 Hz, 1H), 3.69-3.64 (m, 1H), 2.80-2.73 (m, 1H), 2.62 (bs, 1H), 2.25-2.16 (m, 2H), 1.98-1.91 (m, 1H), 1.81 (d, *J* = 2.5 Hz, 3H), 1.73-1.66 (m, 1H), 1.42 (dt, *J* = 13.1, 6.5 Hz, 1H), 0.87 (s, 9H), 0.04 (s, 6H); ^{13}C NMR (100 MHz, $CDCl_3$): δ = 80.2, 78.5, 72.7, 65.1, 42.8, 42.7, 38.4, 30.2, 26.0, 18.2, 3.7, −4.6, −4.7; IR (film) ῡ = 3414br, 2954, 2929, 2885, 2857, 1472, 1463, 1361, 1256, 1099, 1034, 896, 835, 775, 738; MS (EI): *m/z*: 268 (0.2), 253 (1), 211 (50), 193 (6), 181 (14), 169 (17), 155 (10), 141 (18), 119 (37), 105 (8), 91 (28), 75 (100); HRMS (ESI): *m/z*: calc. for $C_{15}H_{28}O_2SiNa$: 291.1751 $[M+Na]^+$, found: 291.1749.

Compound 12. Lactone **9** (8.85 g, 20.7 mmol) was added to a solution of PPh_3 (71.5 g, 273 mmol) in

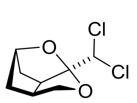

THF (800 mL) and the resulting mixture was stirred at reflux temperature (oil bath temperature ca. 80 °C) when a solution of CCl_4 (83 mL, 132 g, 860 mmol) in THF (50 mL) was added dropwise over a period of 3.5 h. Once the addition was complete, stirring was continued at this temperature for an additional 3 h, before the mixture was allowed to cool and the reaction was quenched with water (10 mL). The mixture was extracted with *tert*-butyl methyl ether (3 x 200 mL), the combined organic phases were washed with sat. aq. $NaHCO_3$ (100 mL) and brine (50 mL), before they were dried over Na_2SO_4, filtered and concentrated under reduced pressure (*Note*: GC-MS shows that the desired dichloro-olefin **10** was the major component at this point). The crude product was suspended in CH_2Cl_2 (ca. 40 mL) and the solution was ultrasonicated in a laboratory cleaning bath for 1 min. The obtained slurry was purified by flash chromatography (SiO_2, hexane/EtOAc, 20/1 to 4/1) to give product **12** as a white solid (4.59 g, 64%). m.p. = 51-54 °C (hexane/EtOAc 4/1); $[\alpha]_D^{20} = +38.2$ (c = 0.8, $CHCl_3$); ^1H NMR (400 MHz, $CDCl_3$): δ = 5.67 (s, 1H), 4.46 (d, *J* = 0.5, 1H), 4.01 (dd, *J* = 8.2, 3.7 Hz, 1H), 3.91 (d, *J* = 8.2, 1H), 2.97-2.96 (m, 1H), 2.70-2.65 (m, 1H), 2.09-2.00 (m, 2H), 1.72-1.68 (m, 2H); ^{13}C NMR (100 MHz, $CDCl_3$): δ = 112.5, 76.6, 74.0, 71.8, 48.5, 39.5, 38.4, 37.8; IR (film) ῡ = 2999, 2973, 2957, 2883, 1437, 1310, 1214, 1140, 1056, 998, 920, 760, 730; MS (EI): *m/z*: 208 (4), 172 (16), 164 (16), 137 (100), 125 (22), 107 (25), 97 (24), 80 (100), 67 (94); HRMS (ESI): *m/z*: calc. for $C_8H_{10}O_2Cl_2Na$: 230.9950 $[M+Na]^+$, found: 230.9951. Crystals suitable for X-ray diffraction were obtained by sublimation of a sample at 40°C in vacuum (10^{-3} mbar).

(1*S*,2*R*,4*S*)-4-[(*tert*-Butyldimethylsilyl)oxy]-2-(prop-1-yn-1-yl)cyclopentane-1-carbaldehyde (S1).

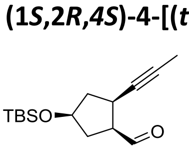

Pyridine (5.80 mL, 71.7 mmol) and Dess-Martin-periodinane (5.63 g, 13.3 mmol) were successively added to a solution of alcohol **11** (2.38 g, 8.8 mmol) in CH_2Cl_2 (46 mL). The mixture was stirred for 3 h at room temperature before the reaction was quenched with sat. aq. $NaHCO_3$ (ca. 10 mL). The mixture was extracted with *tert*-butyl methyl ether (3 x 30 mL), the combined organic layers were washed with aq. $CuSO_4$ (1 M, 4 x 10 mL) and brine (2 x 5 mL) before they were dried over Na_2SO_4. Evaporation of the solvent followed by purification of the crude product by flash chromatography (SiO_2, hexane/EtOAc, 40/1) yielded the title compound in the form of a colorless oil (2.09 g, 89%). $[\alpha]_D^{20} = +31.4$ (c = 1.0, CH_2Cl_2); ^1H NMR (400 MHz, $CDCl_3$): δ =

9.92 (d, *J* = 3.2 Hz, 1H), 4.24 (quint, *J* = 5.9 Hz, 1H), 3.00 (tdd, *J* = 11.0, 5.5, 2.5 Hz, 1H), 2.72-2.65 (m, 1H), 2.4 (ddd, *J* = 13.8, 8.3, 6.0 Hz, 1H), 1.99-1.96 (m, 2H), 1.79-1.72 (m, 1H), 1.78 (d, *J* = 2.5 Hz, 3H), 0.87 (s, 9H), 0.05 (s, 3H), 0.04 (s, 3H); ^{13}C NMR (100 MHz, $CDCl_3$): δ = 204.1, 79.9, 78.3, 72.9, 51.5, 43.2, 36.8, 29.5, 25.9, 18.1, 3.7, –4.65, –4.68; IR (film) ῦ = 2930, 2857,1723, 1472, 1361, 1255, 1115, 896, 775; MS (EI): *m/z*: 266 (0.12), 251 (0.89), 209 (72), 179 (3), 165 (4), 143 (100), 129 (7), 117 (4), 91 (4), 75 (77); HRMS (ESI): *m/z*: calc. for $C_{15}H_{26}O_2SiNa$: 289.1594 $[M+Na]^+$, found: 289.1596.

(1*R*,2*R*,4*S*)-4-[(*tert*-Butyldimethylsilyl)oxy]-2-(prop-1-yn-1-yl)cyclopentane-1-carbaldehyde (13).

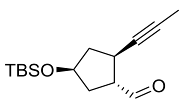

K_2CO_3 (2.17 g, 15.7 mmol) was added in one portion to a solution of aldehyde **S1** (2.09 g, 7.84 mmol) in MeOH (200 mL) and the resulting mixture was stirred for 3 h at room temperature. EtOAc (ca. 70 mL) was added, followed by aq. sat. NH_4Cl. The aqueous phase was extracted with EtOAc (2 x 70 mL) and the combined organic layers were washed with brine (5 mL), dried over Na_2SO_4, filtered and concentrated under reduced pressure. The remaining crude material was purified by flash chromatography (SiO_2, hexane/toluene, 2/1) to give the title compound as colorless oil (1.84 g, 88%). $[\alpha]_D^{20} = -22.4$ (c = 1.0, CH_2Cl_2); ^{1}H NMR (400 MHz, $CDCl_3$): δ = 9.73 (d, *J* = 1.8, 1H), 4.19 (quint., *J* = 5.7 Hz, 1H), 3.07 (qd, *J* = 9.0, 1.8 Hz, 1H), 2.79 (dddd, *J* = 11.3, 9.0, 6.7, 2.4 Hz, 1H), 2.28 (ddd, *J* = 13.6, 8.3, 5.8 Hz, 1H), 1.93 (ddd, *J* = 13.5, 8.6, 6.4 Hz, 1H), 1.83 (ddd, *J* = 13.3, 9.2, 4.2 Hz, 1H), 1.78 (d, *J* = 2.4 Hz, 3H), 1.76-1.69 (m, 1H), 0.88 (s, 9H), 0.04 (s, 3H), 0.04 (s, 3H); ^{13}C NMR (100 MHz, $CDCl_3$): δ = 202.3, 80.6, 77.4, 72.2, 57.1, 43.5, 35.9, 29.1, 25.9, 18.2, 3.7, –4.64, –4.66; IR (film) ῦ = 3097, 2929, 2857, 1725, 1331, 1302, 1253, 1112, 835, 773; MS (EI): *m/z*: 209 (100), 195 (3), 179 (3), 169 (8), 151 (3), 143 (14), 117 (10), 105 (20), 97 (8), 91 (4), 75 (77); HRMS (ESI): *m/z*: calc. for $C_{15}H_{26}O_2SiNa$: 289.1594 $[M+Na]^+$, found: 289.1594.

1-Bromo-3-pentyne (14).[13] Br_2 (6.65 g, 41.6 mmol) was added dropwise at 0 °C to a solution of PPh_3

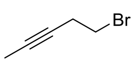

(11.7 g, 44.6 mmol) in MeCN (62 mL) and Et_2O (114 mL) and the resulting mixture was stirred for 20 min at this temperature. Imidazole (3.0 g, 44.66 mmol) was then added in portions before 3-pentyn-1-ol (2.5 g, 29.7 mmol) was slowly introduced. The slurry was stirred for 30 min at 0 °C and for 2 h at ambient temperature. The reaction was quenched with sat. aq. $NaHCO_3$ and the aqueous phase extracted with pentane (2 x 100 mL). The combined extracts were dried over Na_2SO_4, filtered and concentrated (450-350 mbar, 30 °C bath temperature). The obtained solution was filtered twice through a large pad of silica, eluting with pentane. Concentration of the pentane fractions (350 mbar, 30 °C bath temperature) gave the title compound as clear oil (4.35 g, quant.). ^{1}H NMR (400 MHz, $CDCl_3$): δ = 3.41 (t, *J* = 7.4, 2H), 2.69 (tq, *J* = 2.5, 7.3 Hz, 2H), 1.79 (t, *J* = 2.5 Hz, 3H); ^{13}C NMR (100 MHz, $CDCl_3$): δ = 78.1, 76.2, 30.5, 23.5, 3.6; IR (film) ῦ = 2969, 2919, 2855, 1436, 1336, 1271, 1212, 919, 871, 745, 698, 637, 566, 503; MS (EI): *m/z*: 148 (42), 146 (43), 93 (2), 67 (100), 53, (16), 41 (41); HRMS (EI): *m/z*: calc. for C_5H_7Br: 145.9732 [M], found: 145.9731.

(*R*)-6-Octyn-2-ol (S2).[14] Bromide **14** (3.59, 24.4 mmol) was added dropwise to a suspension of

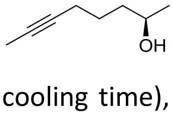

activated magnesium[15] (832 mg, 34.2 mmol) at 0 °C. The mixture was stirred for 30 min at 0 °C and for 40 min at room temperature. After cooling to –78 °C (15 min cooling time), CuCN (219 mg, 2.44 mmol) followed by (*R*)-propylene oxide (1.14 mL, 947 mg, 16.3

mmol) was introduced. The resulting mixture was stirred for 30 min at –78 °C before the cooling bath was removed and stirring was continued for 16 h. For work up, the reaction was quenched at 0°C by the careful addition of sat. aq. NH_4Cl (ca. 30 mL), the obtained slurry was filtered through a pad of Celite to remove the remaining magnesium powder and the filtrate was extracted with *tert*-butyl methyl ether (4 x 50 mL). The combined organic phases were dried over Na_2SO_4, filtered and concentrated, and the residue was subjected to flash chromatography (SiO_2, hexane/*tert*-butyl methyl ether, 4/1) to yield the title compound as a pale yellow oil (1.8 g, 88%). $[\alpha]_D^{20} = -10.6$ (c = 1.2, $CHCl_3$); ^{1}H NMR (400 MHz, $CDCl_3$): δ = 3.83-3.80 (m, 1H), 2.19-2.13 (m, 2H), 1.78 (t, *J* = 2.5 Hz, 3H), 1.63-1.48 (m, 4H), 1.36 [s(br), 1H], 1.20 (d, *J* = 6.2 Hz, 3H); ^{13}C NMR (100 MHz, $CDCl_3$): δ = 79.1, 76.0, 67.9, 38.6, 25.4, 23.7, 18.9, 3.6; IR (film) ῦ = 3351br, 2965, 2920, 2863, 1455, 1435, 1373, 1331, 1182, 1127, 1084, 1045, 1004, 990, 943, 862, 733; MS (EI): *m/z*: 111 (23), 93 (84), 91 (20), 84 (71), 79 (24), 77 (26), 71 (41), 66 (100), 54 (41), 45 (78); HRMS (EI): *m/z*: calc. for $C_8H_{14}O$: 126.1046 [M], found: 126.1045.

(*S*)-Oct-6-yn-2-yl propiolate (16). Propiolic acid (1.19 g, 16.6 mmol) was dissolved in THF (15 mL) and

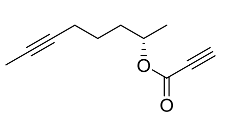

the solution cooled to 0°C before diisopropyl azodicarboxylate (2.42 mL, 2.46 g, 12.2 mmol) was added. Next, a solution of alcohol **S2** (1.40 g, 11.1 mmol) and PPh_3 (3.79 g, 14.4 mmol) in THF (15 mL) was introduced over the course of 1 h. The resulting mixture was allowed to warm to room temperature and stirring was continued for 16 h. *tert*-Butyl methyl ether (100 mL) was added and the obtained red solution was washed with H_2O_2 (10 wt-% in water, 3 x 30 mL) and sat. aq. $NaHCO_3$ (50 mL). The organic phase was dried over Na_2SO_4, filtered and concentrated to ca. 10 mL (250 mbar). This residue was subjected to flash chromatography (SiO_2, hexane/*tert*-butyl methyl ether, 30/1) to give the title compound as pale yellow oil (1.30 g, 66%). $[\alpha]_D^{20} = +28.8$ (c = 1.2, $CHCl_3$); ^{1}H NMR (400 MHz, $CDCl_3$): δ = 5.04 (dqd, *J* = 7.5, 6.2, 5.1 Hz, 1H), 2.85 (s, 1H), 2.15 (tq, J_1 = 7.0, 2.6 Hz, 2H), 1.77 (t, *J* = 2.6 Hz, 3H), 1.75-1.47 (m, 4H), 1.29 (d, *J* = 6.2 Hz, 3H); ^{13}C NMR (100 MHz, $CDCl_3$): δ = 152.5, 78.6, 76.3, 75.2, 74.3, 73.6, 35.0, 24.9, 19.9, 18.7, 3.6; IR (film) ῦ = 3264, 2981, 2939, 2865, 2115, 1453, 1381, 1231, 1130, 1080, 755; MS (EI): *m/z*: 163 (0.4), 135 (3), 121 (5), 108 (12), 93 (95), 79 (25), 66 (100), 53 (86); HRMS (ESI): *m/z*: calc. for $C_{11}H_{14}O_2Na$: 201.0887 $[M+Na]^+$, found: 201.0886.

Preparation of (*R*)-bis(3,5-di-*tert*-Butylphenyl)(1-methylpyrrolidin-2-yl)methanol (25). *This ligand*

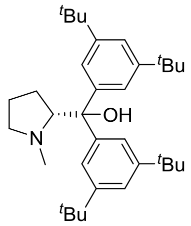

was prepared in analogy to a literature protocol.[9][16] Magnesium turnings (682 mg, 28.1 mmol) were combined with iodine (ca. 4 mg) in a 2-neck round bottom flask, equipped with a reflux condenser. The iodine was sublimed *via* a heatgun and THF (6 mL) was introduced after the iodine vapor had settled, followed by the dropwise addition of a solution of the 3,5-bis-*tert*-butylphenylbromide in THF (10 mL). The reaction was initiated *via* gentle heating after addition of ca. 1/3 of the bromide. Once the addition was complete, the mixture was stirred at 80 °C for 2 h. The resulting solution was slowly added to a solution of benzyl 2-methyl (*R*-pyrrolidine)-1,2-dicarboxylate (N-Cbz D-proline methyl ester, 1.79 g, 6.80 mmol) in THF (7.4 mL) at 0 °C. The mixture was warmed to room

temperature and stirred for 2 h. The reaction was carefully quenched with sat. aq. NH_4Cl (10 mL) and extracted with *tert*-butyl methyl ether (3 x 100 mL). The combined organic phases were washed with sat. aq. $NaHCO_3$ (30 mL) and brine (30 mL) before they were dried over Na_2SO_4, filtered and concentrated. The residue was purified by flash chromatography (SiO_2, hexane/EtOAc, 9/1) to give a mixture of the target compound **25** and di-*tert*-butylbenzene.

This mixture was dissolved in THF (90 mL) and cooled to 0 °C before $LiAlH_4$ (684 mg, 18.0 mmol) was added portionwise over a period of 5 min. The cooling bath was removed and the mixture stirred at 90 °C for 30 min. The reaction was carefully quenched at 0°C with sat. aq. NH_4Cl (ca. 10 mL). A saturated aqueous solution of Rochelle's salt (5 mL) was added and the mixture was stirred for 45 min. Insoluble material was filtered off through a pad of Celite, the filtrate was checked for a pH > 8 and extracted with *tert*-butyl methyl ether (3 x 100 mL). The combined organic phases were dried over Na_2SO_4, filtered and concentrated to give a colorless oil containing small amounts of a white solid. This material was dissolved in hexane and kept at 4 °C to allow for crystallization (scratching of the flask with a glass rod was necessary). After 16 h, compound **25** was collected as a white amorphous solid (2.58 g, 77%); after concentration of the mother liquor and repeated crystallization, a second crop of product was obtained (380 mg, 11%). m.p. = 138-142 °C (hexane); $[\alpha]_D^{20} = -13.7$ (c = 1.0, $CHCl_3$); ^1H NMR (400 MHz, $CDCl_3$): δ = 7.55 (d, *J* = 1.8 Hz, 2H), 7.41 (d, *J* = 1.8 Hz, 2H), 7.16 (d, *J* = 2.8, 1.8 Hz, 2H), 4.65 (s, 1H), 3.58 (dd, *J* = 9.5, 4.9 Hz, 1H), 3.08 (dd, *J* = 9.5, 7.4 Hz, 1H), 2.45-2.39 (m, 1H), 1.82-1.77 (m, 1H), 1.73 (s, 3H), 1.71-1.57 (m, 3H), 1.30 (s, 18H), 1.29 (s, 18H); ^{13}C NMR (100 MHz, $CDCl_3$): δ = 149.9, 149.8, 147.7, 146.2, 120.1, 119.8, 119.7, 119.5, 77.9, 73.2, 59.5, 43.0, 35.1, 35.0, 31.74, 31.72, 30.0, 24.0; IR (film) ῦ = 2962, 2904, 2868, 2787, 1598, 1362, 1214, 742, 668; MS (EI): *m/z*: 476 (0.3), 407 (0.7), 392 (2), 302 (0.6), 217 (4), 161 (2), 133 (1), 84 (100), 57 (5); HRMS (ESI): *m/z*: calc. for $C_{34}H_{54}NO$: 492.4200 $[M+H]^+$, found: 492.4200.

(*S*)-Oct-6-yn-2-yl (*S*)-4-{(1*R*,2*R*,4*S*)-4-[(*tert*-butyldimethylsilyl)oxy]-2-(prop-1-yn-1-yl)cyclopentyl}-4-hydroxybut-2-ynoate (17).[9] Compound **25** (101 mg, 0.21 mmol, 27.5 mol-%) was dissolved in dry toluene (600 µL). Ester **16** (207 mg, 1.16 mmol) was added, followed by careful addition of Me_2Zn (1.2 M in toluene, 940 µL, 1.13 mmol) and a solution of aldehyde **13** (200 mg, 0.75 mmol) in toluene (840 µL). The mixture was stirred for 16 h at ambient temperature before the reaction was carefully quenched with sat. aq. NH_4Cl. The mixture was extracted with EtOAc (3 x 25 mL), the combined organic phases were dried over Na_2SO_4, filtered and concentrated under reduced pressure. The remaining yellow oil was purified by flash chromatography (SiO_2, toluene/EtOAc, 50/1) to give the title compound as colorless oil (247 mg, 74%).

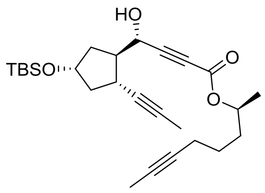

The reaction was also performed on larger scale, using aldehyde **13** (1.67 g, 6.28 mmol), ester **16** (1.73 g, 9.73 mmol), dimethylzinc (1.2 M in toluene, 7.85 mL, 9.42 mmol), **25** (772 mg, 1.57 mmol, 25 mol-%) and toluene (12 mL) to give the title compound (1.84 g, 66%), which analyzed as follows: $[\alpha]_D^{20} = -12.3$ (c = 1.0, CH_2Cl_2); ^1H NMR (400 MHz, $CDCl_3$): δ = 5.02 (ddq, *J* = 7.6, 6.2, 5.1 Hz, 1H), 4.68 (dd, *J* = 7.0, 3.3 Hz, 1H), 4.23 (p, *J* = 5.8 Hz, 1H), 2.63-2.46 (m, 2H), 2.39 (d, *J* = 7.0 Hz, 1H), 2.31 (ddd, *J*

= 13.4, 7.5, 6.1 Hz, 1H), 2.17-2.12 (m, 2H), 1.78-1.77 (m, 6H), 1.75-1.40 (m, 7H), 1.28 (d, *J* = 6.2 Hz, 3H), 0.88 (s, 9H), 0.04 (s, 6H); ^{13}C NMR (100 MHz, $CDCl_3$): δ = 153.1, 86.3, 81.1, 78.6, 77.7, 77.4, 76.2, 73.3, 71.8, 63.6, 49.8, 43.8, 36.6, 35.0, 30.0, 26.0, 24.9, 20.0, 18.7, 18.2, 3.7, 3.6, −4.61, −4.63; IR (film) ῦ = 3461br, 2929, 2856, 2233, 1709, 1462, 1378, 1360, 1252, 1112, 1051, 836, 775; MS (EI): *m/z*: 401 (5), 387 (30), 279 (39), 209 (12), 187 (14), 169 (11), 161 (11), 159 (13), 141 (12), 131 (17), 115 (11), 109 (93), 105 (19), 91 (20), 75 (100), 67 (42); HRMS (ESI): *m/z*: calc. for $C_{26}H_{40}O_4SiNa$: 467.2597 $[M+Na]^+$, found: 467.2588.

(*S*)-Oct-6-yn-2-yl (*R*,*E*)-4-{(1*R*,2*R*,4*S*)-4-[(*tert*-butyldimethylsilyl)oxy]-2-(prop-1-yn-1-yl)cyclopentyl}-4-hydroxybut-2-enoate (S3).[10] Red-Al (65 wt-% in toluene, 3.03 mL, 10.1 mmol) was added dropwise at −78 °C to a solution of compound **17** (2.25 g, 5.05 mmol) in THF (76 mL) and the resulting mixture was stirred at this temperature for 20 min. Sat. aq. NH_4Cl was then used to quench the reaction. The resulting mixture was allowed to warm to room temperature before it was extracted with EtOAc [4 x 50 mL, in order to facilitate extraction, saturated aq. Rochelle's salt solution (15 mL) was added]. The combined organic phases were dried over Na_2SO_4, filtered and concentrated under reduced pressure. The residue was purified by flash chromatography (SiO_2, hexane/EtOAc , 12/1) to give the title compound as pale yellow oil (2.10 g, 93%). $[\alpha]_D^{20} = -9.5$ (c = 1.0, CH_2Cl_2); ^{1}H NMR (400 MHz, $CDCl_3$): δ = 6.98 (dd, *J* = 4.5, 15.6 Hz, 1H), 6.06 (dd, *J* = 1.8, 15.6 Hz, 1H), 4.99 (sext., *J* = 6.3 Hz, 1H), 4.52-4.48 (m, 1H), 4.21-4.15 (m, 1H), 2.51-2.43 (m, 1H), 2.38-2.24 (m, 2H), 2.14 (tq, *J* = 7.0, 2.5 Hz, 2H), 1.79-1.77 (m, 6H), 1.73-1.46 (m, 7H), 1.26 (d, *J* = 6.2 Hz, 3H), 0.87 (s, 9H), 0.03 (s, 3H), 0.02 (s, 3H); ^{13}C NMR (100 MHz, $CDCl_3$): δ = 166.2, 149.1, 121.2, 81.3, 78.8, 76.9, 76.1, 72.0, 71.1, 70.9, 49.8, 43.7, 35.3, 35.2, 30.6, 26.0, 25.1, 20.2, 18.8, 18.2, 3.7, 3.6, −4.60, −4.61; IR (film) ῦ = 3472br, 2952, 2929, 2857, 1716, 1699, 1462, 1360, 1254, 1172, 1114, 1084, 1051, 903, 835, 774; MS (EI): *m/z*: 403 (8), 389 (50), 347 (15), 281 (18), 189 (18), 169 (15), 129 (17), 109 (75), 75 (100), 67 (46); HRMS (ESI): *m/z*: calc. for $C_{26}H_{42}O_4SiNa$: 469.2745 $[M+Na]^+$, found: 469.2745.

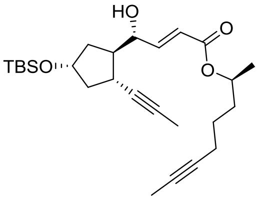

(*S*)-Oct-6-yn-2-yl (*R*,*E*)-4-[(*tert*-butyldimethylsilyl)oxy]-4-{(1*R*,2*R*,4*S*)-4-[(*tert*-butyldimethylsilyl)oxy]-2-(prop-1-yn-1-yl)cyclopentyl}but-2-enoate (18). Pyridine (614 µL, 600 mg, 7.59 mmol) and TBSOTf (871 µL, 1.00 g, 3.79 mmol) were added at 0 °C to a solution of compound **S3** (1.13 g, 2.53 mmol) in CH_2Cl_2, and the resulting mixture was stirred at this temperature for 30 min. The reaction was quenched with sat. aq. $NaHCO_3$ and the mixture extracted with EtOAc (3 x 20 mL). The combined organic phases were washed with aq. $CuSO_4$ (1 M, 5 x 10 mL) and brine (10 mL), dried over Na_2SO_4, filtered and concentrated under reduced pressure. The crude product was purified by flash chromatography (neutral Al_2O_3, hexane/EtOAc, 20/1) to give the title compound as colorless oil (1.32 g, 93%). $[\alpha]_D^{20} = -16.4$ (c = 1.0, $CHCl_3$); ^{1}H NMR (400 MHz, $CDCl_3$): δ = 6.89 (dd, *J* = 4.8, 15.6 Hz, 1H), 5.93 (dd, *J* = 1.7, 15.6 Hz, 1H), 4.98 (dq, *J* = 12.4, 6.2 Hz, 1H), 4.48-4.46 (m, 1H), 4.16-4.10 (m, 1H), 2.43-2.34 (m, 1H), 2.27-2.11 (m, 4H), 1.79-1.77 (m, 6H), 1.75-1.58 (m, 4H), 1.54-1.39 (m, 3H), 1.26 (d, *J* = 6.2 Hz, 3H), 0.92 (s, 9H), 0.86 (s, 9H), 0.08 (s, 3H), 0.02 (s, 3H), 0.02 (s, 3H), 0.01 (s, 3H); ^{13}C

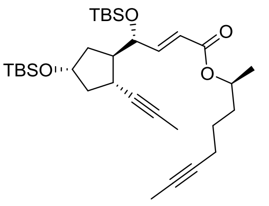

NMR (100 MHz, $CDCl_3$): δ = 166.3, 150.6, 120.6, 81.4, 78.8, 76.4, 76.0, 72.2, 70.74, 70.72, 50.5, 43.7, 35.3, 34.3, 30.7, 26.04, 26.02, 25.1, 20.2, 18.8, 18.31, 18.27, 3.7, 3.6, –4.1, –4.6, –5.0; IR (film) ῦ = 2954, 2930, 2898, 2857, 1719, 1658, 1472, 1463, 1361, 1256, 1170, 1129, 712, 776; MS (EI): *m/z*: 503 (83), 395 (18), 371 (23), 295 (9), 263 (33), 245 (13), 197 (10), 171 (19), 109 (64), 75 (75), 73 (100), 67 (33); HRMS (ESI): *m/z*: calc. for $C_{32}H_{56}O_4Si_2Na$: 583.3611 $[M+Na]^+$, found: 583.3609.

Cycloalkyne 19. A flame-dried round bottom flask, equipped with a reflux condenser, was charged

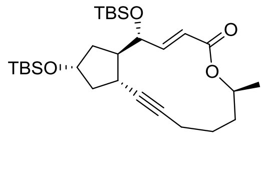

with molecular sieves [5Å, 3.75 g, powder (dried prior to use at 150 °C under high vacuum)]. A solution of diyne **18** (1.25 g, 2.23 mmol) in dry toluene (560 mL) was added and the slurry was stirred for 30 min before it was heated to 80 °C (oil bath temperature). The reaction was initiated by the addition of a solution of complex **26** (123 mg, 118 µmol, 5 mol-%) in toluene (5 mL). After stirring for 45 min the mixture was diluted with EtOAc (60 mL) and filtered through a pad of neutral Al_2O_3 which was rinsed with EtOAc (70 mL). The combined filtrates were concentrated under reduced pressure and the residue purified by flash chromatography (neutral Al_2O_3, hexane/EtOAc, 80/1) to give the title compound as colorless oil (752 mg, 67%). $[\alpha]_D^{20} = -28.6$ (c = 1.1, $CHCl_3$); ^1H NMR (400 MHz, $CDCl_3$): δ = 7.59 (dd, *J* = 2.9, 15.4 Hz, 1H), 5.95 (dd, *J* = 1.9, 15.4 Hz, 1H), 5.02-4.93 (m, 1H), 4.21-4.14 (m, 1H), 3.96 (ddd, *J* = 1.9, 3.0, 9.4 Hz, 1H), 2.35-2.14 (m, 4H), 2.01-1.88 (m, 2H), 1.79-1.60 (m, 4H), 1.56-1.38 (m, 2H), 1.28 (d, *J* = 6.4 Hz, 3H), 0.93 (s, 9H), 0.87 (s, 9H), 0.05 (s, 3H), 0.03 (s, 9H); ^{13}C NMR (100 MHz, $CDCl_3$): δ = 166.6, 152.4, 118.5, 82.9, 81.9, 76.1, 71.8, 71.0, 53.9, 44.3, 41.9, 36.0, 31.5, 29.9, 26.0, 24.8, 21.1, 19.4, 18.3, 18.2, –4.0, –4.6, –4.7; IR (film) ῦ = 2954, 2929, 2856, 1717, 1463, 1362, 1255, 837, 775; MS (EI): *m/z*: 449 (84), 373 (6), 317 (16), 289 (7), 251 (100), 197 (8), 73 (64); HRMS (ESI): *m/z*: calc. for $C_{28}H_{50}O_4Si_2Na$: 529.3142 $[M+Na]^+$, found: 529.3140.

Compound 20. HCl (2 M, 1 mL) was added to a solution of compound **19** (75 mg, 0.15 mmol) in THF

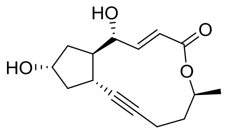

(5.5 mL) and water (5.5 mL). The mixture was stirred for 39 h before the reaction was quenched with sat. aq. $NaHCO_3$ (ca. 4 mL) and extracted with *tert*-butyl methyl ether (3 x 25 mL). The combined organic phases were dried over Na_2SO_4, filtered and concentrated and the residue was purified by flash chromatography (SiO_2, hexane/EtOAc, 1/2) to give the title compound as a white solid (38 mg, 92%). m.p. = 189-191 °C ($CDCl_3$); $[\alpha]_D^{20} = -13.7$ (c = 0.66, acetone); ^1H NMR (400 MHz, CD_3OD): δ = 7.76 (dd, *J* = 15.5, 2.8 Hz, 1H), 5.90 (dd, *J* = 15.5, 2.0 Hz, 1H), 4.94-4.83 (m, 1H), 4.23-4.18 (m, 1H), 4.01 (dt, *J* = 9.4, 2.3 Hz, 1H), 2.47 (dddt, *J* = 11.2, 9.0, 5.0, 3.1 Hz, 1H), 2.38 (dd, *J* = 13.1, 8.6 Hz, 1H), 2.30-2.23 (m, 1H), 2.10-2.00 (m, 2H), 1.97-1.89 (m, 1H), 1.83-1.70 (m, 3H), 1.69-1.59 (m, 2H), 1.26 (d, *J* = 6.3 Hz, 3H), 1.18-1.10 (m, 1H); ^{13}C NMR (100 MHz, CD_3OD): δ = 168.4, 155.0, 118.0, 83.8, 82.8, 76.1, 72.7, 72.0, 54.5, 44.4, 41.6, 36.9, 32.7, 25.9, 21.1, 19.9; IR (film) ῦ = 3364 (br), 3278 (br), 2970, 2948, 2926, 2865, 1711, 1438, 1258, 1111, 1064, 987; MS (ESI): *m/z*: 301 $[M+Na]^+$; HRMS (ESI): *m/z*: calc. for $C_{16}H_{22}O_4Na$: 301.1410 $[M+Na]^+$, found: 301.1411. Crystals suitable for X-ray diffraction were obtained by slow evaporation of the solvent from a solution of the compound in MeOH/acetone.

Compound 21. In a flame dried Schlenk tube [Cp*Ru(MeCN)$_3$]PF$_6$ (57 mg, 0.11 mmol, 5 mol-%) was dissolved in CH_2Cl_2 (15 mL). A solution of cycloalkyne **19** (1.15 g, 2.27 mmol) in CH_2Cl_2 (5 mL) was added with stirring before this mixture was transferred under Ar via cannula into a pre-dried autoclave. The Schlenk tube was rinsed with additional CH_2Cl_2 (8 mL), which was also added. The autoclave was pressurized with H_2 (30 bar). After stirring for 4 h, the autoclave was vented and the remaining yellow solution filtered through a pad of neutral Al_2O_3 which was carefully rinsed with EtOAc (50 mL). The combined filtrates were concentrated and the remaining pale brown oil was subjected to flash chromatography (neutral Al_2O_3, hexane/EtOAc, 90/1) to give a mixture of **21** and isomers (981 mg, 85%) (as well as a small amount of overreduced product). This material was purified by preparative HPLC (Nucleodur C18 HTec, 10 µm, 250 x 40, eluent MeOH/H_2O 95/5, 75 mL/min) to give pure **21** as a colorless oil (642 mg, 56%). $[\alpha]_D^{20} =$ +20.1 [c = 0.9, $CHCl_3$, lit.:[11] $[\alpha]_D^{23} =$ +22 (c 0.72, $CHCl_3$)]; ^{1}H NMR (300 MHz, $CDCl_3$): δ = 7.28 (dd, *J* = 3.2, 15.5 Hz, 1H), 5.87 (dd, *J* = 1.8, 15.5 Hz, 1H), 5.62 (ddd, *J* = 4.5, 10.1, 14.9 Hz, 1H), 5.26 (dd, *J* = 9.5, 15.2 Hz, 1H), 4.89 (ddq, *J* = 1.8, 6.3, 10.9 Hz, 1H), 4.22-4.16 (m, 1H), 4.01 (ddd, *J* = 1.7, 3.0, 9.2 Hz, 1H), 2.30-2.17 (m, 1H), 2.09-1.93 (m, 4H), 1.87-1.64 (m, 3H), 1.59-1.42 (m, 3H), 1.25 (d, *J* = 6.2 Hz, 3H), 1.00-0.93 (m, 1H), 0.93 (s, 9H), 0.88 (s, 9H), 0.06 (s, 3H), 0.04 (s, 3H), 0.03 (s, 3H), 0.02 (s, 3H); ^{13}C NMR (75 MHz, $CDCl_3$): δ = 166.6, 152.6, 137.5, 129.5, 118.3, 76.6, 73.0, 71.5, 53.0, 44.0, 43.9, 42.3, 34.3, 32.0, 26.9, 26.0, 21.1, 18.3, 18.2, –3.9, –4.57, –4.60, –4.7; IR (film) ῦ = 2954, 2930, 2857, 1716, 1472, 1462, 1361, 1254, 1122, 1078, 837, 775; MS (EI): *m/z*: 508 (3), 493 (2), 451 (100), 433 (18), 361 (24), 343 (13), 319 (33), 301 (18), 291 (12), 227 (13), 199 (26), 185 (11), 147 (12), 129 (11), 73 (62); HRMS (ESI): *m/z*: calc. for $C_{28}H_{52}O_4Si_2Na$: 531.3297 $[M+Na]^+$, found: 531.3296.

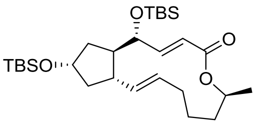

(+)-Brefeldin A (1).[11] HCl (2 M in water, 8.2 mL) was added to a solution of compound **21** (632 mg, 1.24 mmol) in THF (46 mL) and water (46 mL) and the resulting mixture was stirred for 39 h at ambient temperature. The reaction was quenched by the addition of aq. sat. $NaHCO_3$ and the aqueous layer extracted with *tert*-butyl methyl ether (3 x 50 mL). The combined extracts were dried over Na_2SO_4, filtered and concentrated and the residue was purified by flash chromatography (SiO_2, hexane/EtOAc, 1/2) to give (+)-brefeldin A as a white solid (327 mg, 94%). m.p. = 202-204 °C (MeOH, lit.:[12] 202-203 °C) ; ^{1}H NMR (400 MHz, CD_3OD): δ = 7.46 (dd, *J* = 15.6, 3.0 Hz, 1H), 5.82 (dd, *J* = 15.7, 2.0 Hz, 1H), 5.75 (ddd, *J* = 4.6, 10.5, 15.0 Hz, 1H), 5.28 (dd, *J* = 9.6, 15.1 Hz, 1H), 4.80 (ddq, *J* = 1.8, 6.3, 11.0 Hz, 1H), 4.24-4.19 (m, 1H), 4.04 (ddd, *J* = 2.0, 3.1, 9.5 Hz, 1H), 2.39 (quint, *J* = 8.5 Hz, 1H), 2.13 (ddd, *J* = 5.3, 8.8, 13.2 Hz, 1H), 2.05-1.98 (m, 2H), 1.89-1.73 (m, 5H), 1.62-1.54 (m, 1H), 1.45 (dddd, *J* = 1.3, 5.4, 8.0, 13.3 Hz, 1H), 1.24 (d, *J* = 6.2 Hz, 3H), 0.94 – 0.85 (m, 1H); ^{13}C NMR (100 MHz, CD_3OD): δ = 168.3, 155.1, 138.1, 131.4, 117.7, 76.6, 73.2, 73.0, 53.2, 45.4, 44.1, 41.8, 35.0, 33.0, 28.0, 21.1; IR (film) ῦ = 3352 (br), 3307 (br), 2923, 2893, 2854, 2495, 2455, 1709, 1448, 1255, 1109, 1070, 975; MS (ESI): *m/z*: 303 $[M+Na]^+$; HRMS (ESI): *m/z*: calc. for $C_{16}H_{24}O_4Na$: 303.1567 $[M+Na]^+$, found: 303.1567. Crystals suitable for X-ray diffraction were obtained by slow evaporation of the solvent from a solution of the compound in MeOH/acetone.

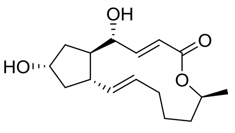

Table S-1. Comparison of ^{1}H NMR data ([D$_4$]-MeOH) of (+)-Brefeldin A

observed		*literature*[12]		
δ, ppm	*J* (Hz)	δ, ppm	*J* (Hz)	Δδ (ppm)
7.46	15.6, 3.0	7.45	15.6, 3.0	0.01
5.82	15.7, 2.0	5.82	15.7, 2.0	0.00
5.75	4.6, 10.5, 15.0	5.75	4.6, 10.2, 15.0	0.00
5.28	9.6, 15.1	5.27	9.6, 15.1	0.01
4.80	1.8, 6.3, 11.0	4.78	-	0.02
4.24-4.19	-	4.21	-	0.00
4.04	2.0, 3.1, 9.5	4.03	-	0.01
2.39	8.5	2.38	8.7	0.01
2.13	5.3, 8.8, 13.2	2.12	5.4, 8.7, 13.6	0.01
2.05-1.98	-	2.05-1.97	-	0.00
1.89-1.73	-	1.90-1.70	-	0.01
1.62-1.54	-	1.55	-	0.03
1.45	1.3, 5.4, 8.0, 13.3	1.42	-	0.03
1.24	6.2	1.23	6.2	0.01
0.94-0.85	-	0.90	-	0.00

Table S-2. Comparison of ^{13}C NMR data ([D$_4$]-MeOH) of (+)-Brefeldin A

observed	*literature*[12]	
δ, ppm	δ, ppm	Δδ (ppm)
168.3	168.7	–0.4
155.1	155.4	–0.3
138.1	138.4	–0.3
131.4	131.7	–0.3
117.7	118.1	–0.4
76.6	76.9	–0.3
73.2	73.5	–0.3
73.0	73.3	–0.3
53.2	53.5	–0.3
45.4	45.8	–0.4
44.1	44.4	–0.3
41.8	42.1	–0.3
35.0	35.3	–0.3
33.0	33.3	–0.3
28.0	28.3	–0.3
21.1	21.3	–0.2

References

[1] (a) J. Heppekausen, R. Stade, R. Goddard, A. Fürstner, *J. Am. Chem. Soc.* **2010**, *132*, 11045-11057; (b) J. Heppekausen, R. Stade, A. Kondoh, G. Seidel, R. Goddard, A. Fürstner, *Chem. Eur. J.* **2012**, *18*, 10281-10299.

[2] M. D. Mbaye, B. Demerseman, J.-L. Renaud, L. Toupet, C. Bruneau, *Adv. Synth. Catal.* **2004**, *346*, 835-841.

[3] J. E. Ladbury, E. E. Turner, *J. Chem. Soc.* **1954**, 3885-3886.

[4] (a) H.-J. Gais, T. Lied, *Angew. Chem.* **1984**, *96*, 143-145; (b) S. Kobayashi, K. Kamiyama, T. Iimori, M. Ohno, *Tetrahedron Lett.* **1984**, *25*, 2557-2560.

[5] Y. N. Ito, X. Ariza, A. K. Beck, A. Boháč, C. Ganter, R. E. Gawley, F. N. M. Kühnle, J. Tuleja, Y. M. Wang, D. Seebach, *Helv. Chim. Acta* **1994**, *77*, 2071-2110.

[6] H.-J. Gais, K. L. Lukas, W. A. Ball, S. Braun, H. J. Lindner, *Liebigs Ann. Chem.* **1986**, 687-716.

[7] Compare: Å. Rosenquist, I. Kvarnström, S. C. T. Svensson, B. Classon, B. Samuelsson, *Acta. Chem. Scandinavia* **1992**, *46*, 1127-1129.

[8] A. M. Gimazetdinov, G. V. Ishmurzina, M. S. Miftakhov, *Russ. J. Org. Chem.* **2012**, *48*, 8-17.

[9] In analogy to: N. Kojima, S. Nishijima, K. Tsuge, T. Tanaka, *Org. Biomol. Chem.* **2011**, *9*, 4425-4428.

[10] Compare: C. T. Meta, K. Koide, *Org. Lett.* **2004**, *6*, 1785-1787.

[11] Y. Wu, X. Shen, Y.-Q. Yang, Q. Hu, J.-H. Huang, *J. Org. Chem.* **2004**, *69*, 3857-3865.

[12] Y. Wu, X. Shen, Y.-Q. Yang, Q. Hu, J.-H. Huang, *Tetrahedron Lett.* **2004**, *45*, 199-202.

[13] Modified from procedure of: V. Hickmann, A. Kondoh, B. Gabor, M. Alcarazo, A. Fürstner, *J. Am. Chem. Soc.* **2011**, *133*, 13471-13480.

[14] P. Persich, J. Llaveria, R. Lhermet, T. de Haro, R. Stade, A. Kondoh, A. Fürstner, *Chem. Eur. J.* **2013**, *19*, 13047-13058.

[15] E. Bartmann, B. Bogdanović, N. Janke, S. Liao, K. Schlichte, B. Spliethoff, J. Treber, U. Westeppe, U. Wilczok, *Chem. Ber.* **1990**, *123*, 1517-1528.

[16] E. J. Corey, S. Shibata, R. K. Bakshi, *J. Org. Chem.* **1988**, *53*, 2861-2863.

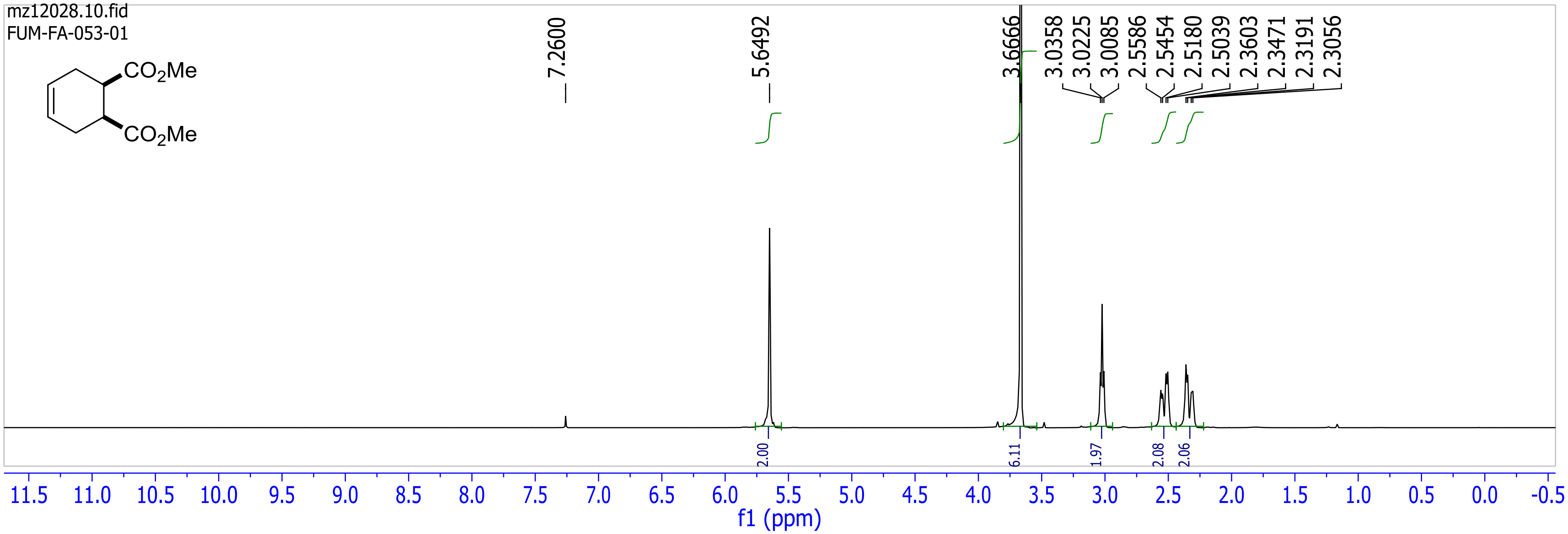
mz12028.10.fid
FUM-FA-053-01
CO2Me
CO2Me
7.2600
5.6492
3.6666
3.0358
3.0225
3.0085
2.5586
2.5454
2.5180
2.5039
2.3603
2.3471
2.3191
2.3056
2.00
6.11
1.97
2.08
2.06
11.5 11.0 10.5 10.0 9.5 9.0 8.5 8.0 7.5 7.0 6.5 6.0 5.5 5.0 4.5 4.0 3.5 3.0 2.5 2.0 1.5 1.0 0.5 0.0 -0.5
f1 (ppm)

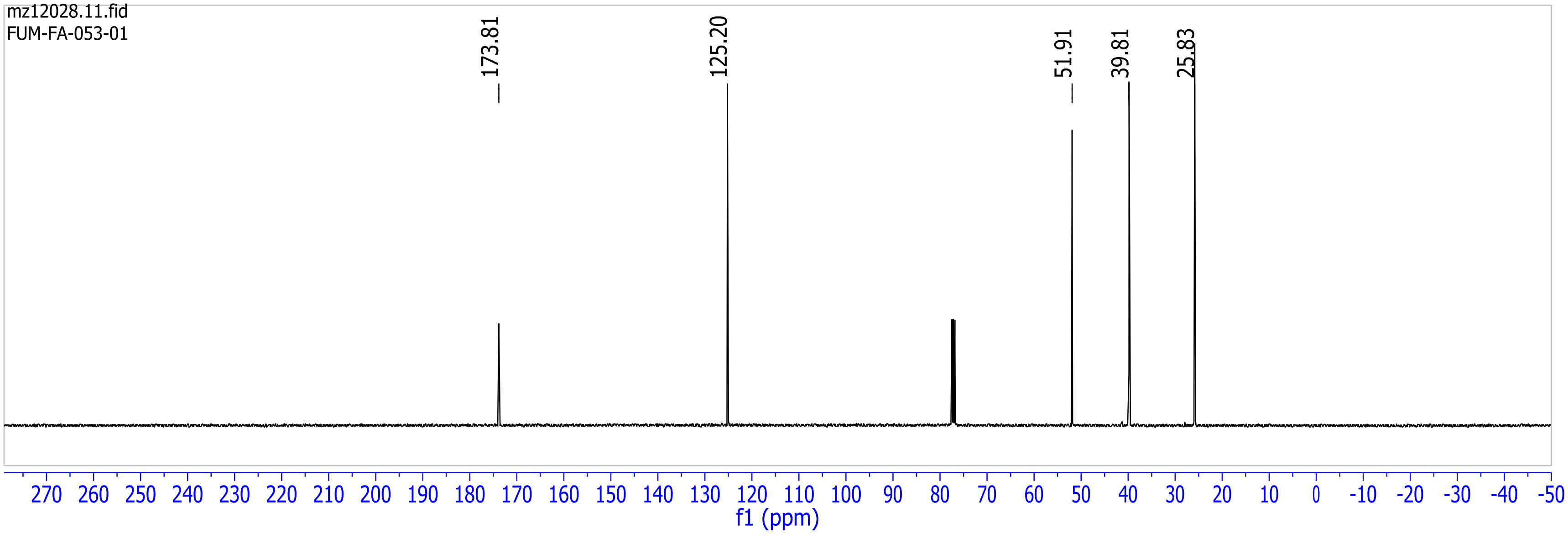
mz12028.11.fid
FUM-FA-053-01
173.81
125.20
51.91
39.81
25.83
270 260 250 240 230 220 210 200 190 180 170 160 150 140 130 120 110 100 90 80 70 60 50 40 30 20 10 0 -10 -20 -30 -40 -50
f1 (ppm)

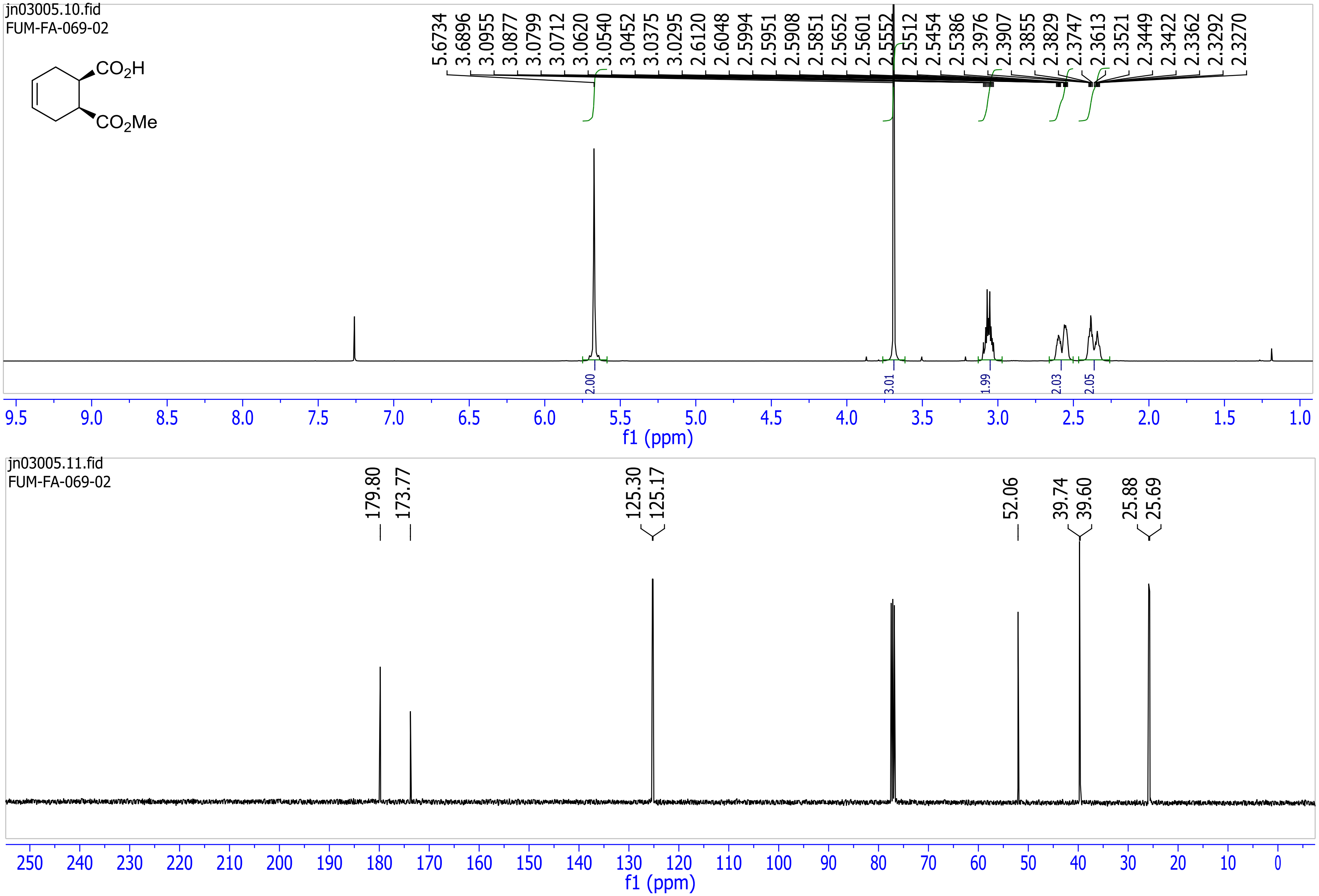
jn03005.10.fid
FUM-FA-069-02
CO_2H
CO_2Me
5.6734
3.6896
3.0955
3.0877
3.0799
3.0712
3.0620
3.0540
3.0452
3.0375
3.0295
2.6120
2.6048
2.5994
2.5951
2.5908
2.5851
2.5652
2.5601
2.5552
2.5512
2.5454
2.5386
2.3976
2.3907
2.3855
2.3829
2.3747
2.3613
2.3521
2.3449
2.3422
2.3362
2.3292
2.3270
2.00
3.01
1.99
2.03
2.05
f1 (ppm)
jn03005.11.fid
FUM-FA-069-02
179.80
173.77
125.30
125.17
52.06
39.74
39.60
25.88
25.69
f1 (ppm)

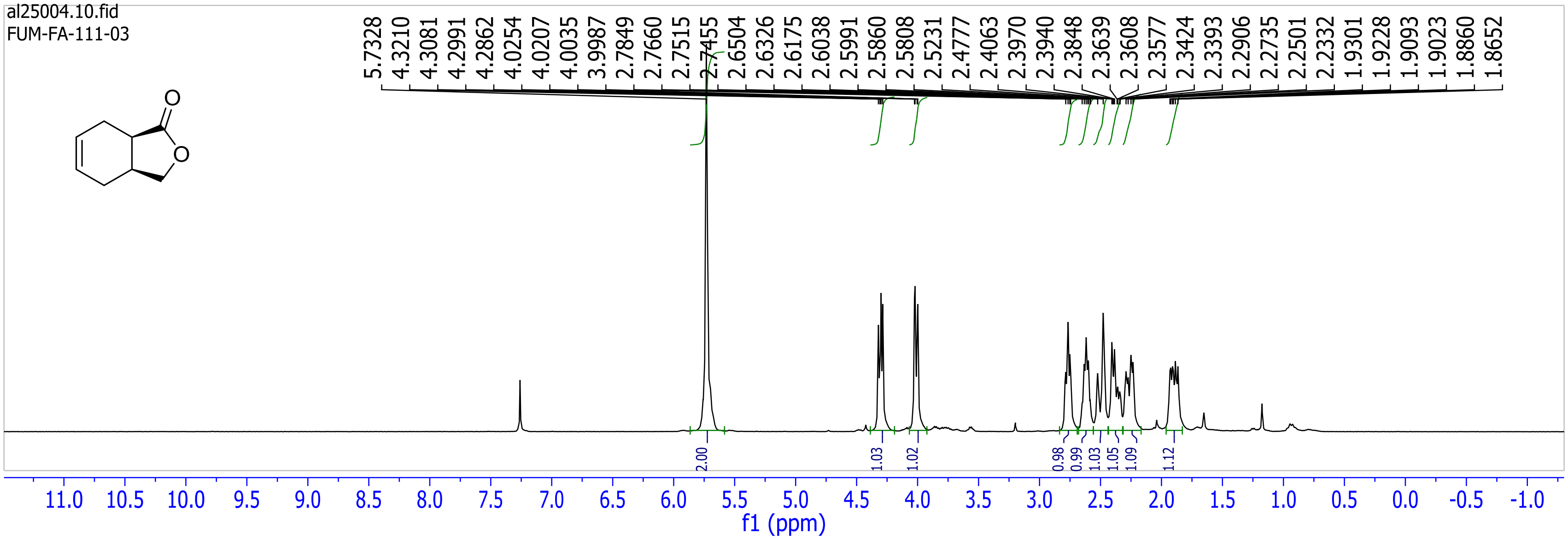
al25004.10.fid
FUM-FA-111-03
O
O
5.7328
4.3210
4.3081
4.2991
4.2862
4.0254
4.0207
4.0035
3.9987
2.7849
2.7660
2.7515
2.7455
2.6504
2.6326
2.6175
2.6038
2.5991
2.5860
2.5808
2.5231
2.4777
2.4063
2.3970
2.3940
2.3848
2.3639
2.3608
2.3577
2.3424
2.3393
2.2906
2.2735
2.2501
2.2332
1.9301
1.9228
1.9093
1.9023
1.8860
1.8652
2.00
1.03
1.02
0.98
0.99
1.03
1.05
1.09
1.12
11.0 10.5 10.0 9.5 9.0 8.5 8.0 7.5 7.0 6.5 6.0 5.5 5.0 4.5 4.0 3.5 3.0 2.5 2.0 1.5 1.0 0.5 0.0 -0.5 -1.0
f1 (ppm)

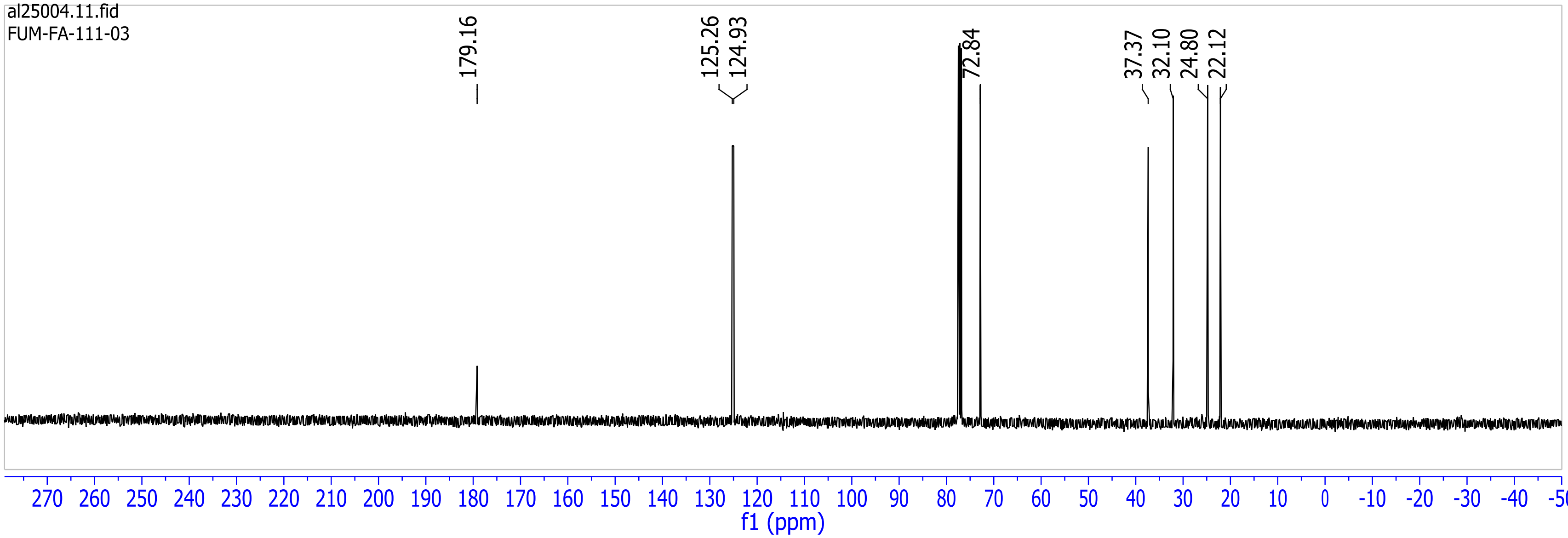
al25004.11.fid
FUM-FA-111-03
179.16
125.26
124.93
72.84
37.37
32.10
24.80
22.12
270 260 250 240 230 220 210 200 190 180 170 160 150 140 130 120 110 100 90 80 70 60 50 40 30 20 10 0 -10 -20 -30 -40 -50
f1 (ppm)

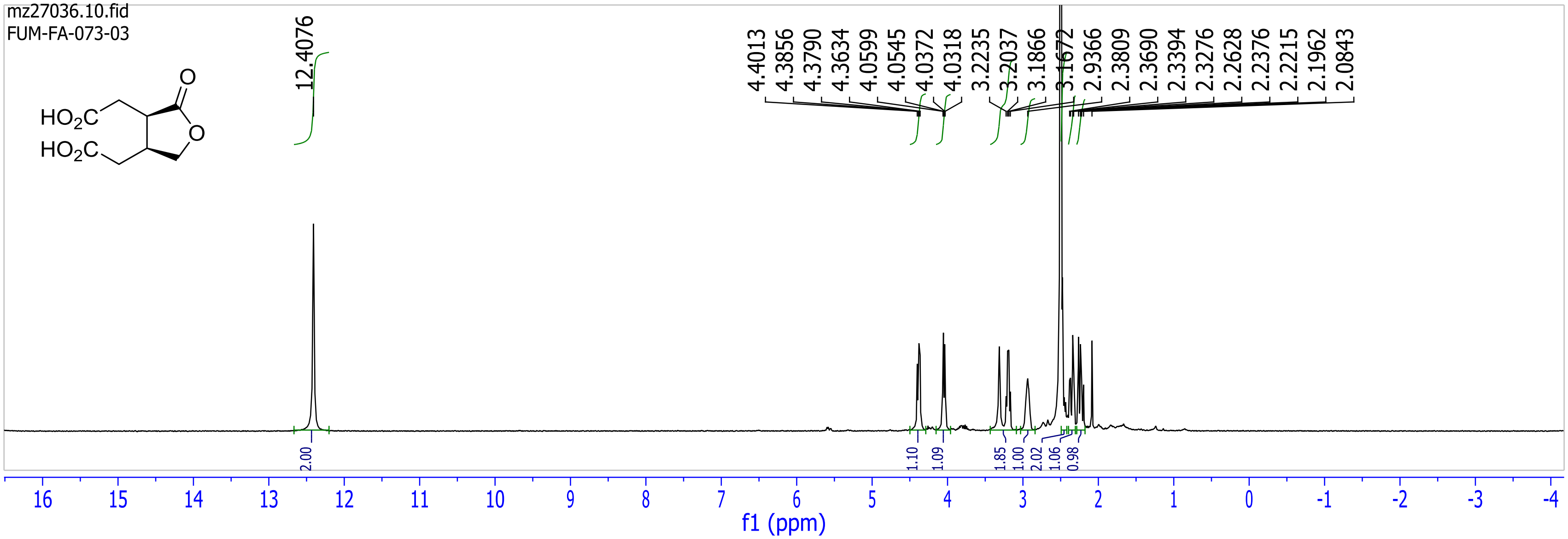
mz27036.10.fid
FUM-FA-073-03
HO2C
HO2C
O
O
12.4076
4.4013
4.3856
4.3790
4.3634
4.0599
4.0545
4.0372
4.0318
3.2235
3.2037
3.1866
3.1672
2.9366
2.3809
2.3690
2.3394
2.3276
2.2628
2.2376
2.2215
2.1962
2.0843
2.00
1.10
1.09
1.85
1.00
2.02
1.06
0.98
16 15 14 13 12 11 10 9 8 7 6 5 4 3 2 1 0 -1 -2 -3 -4
f1 (ppm)

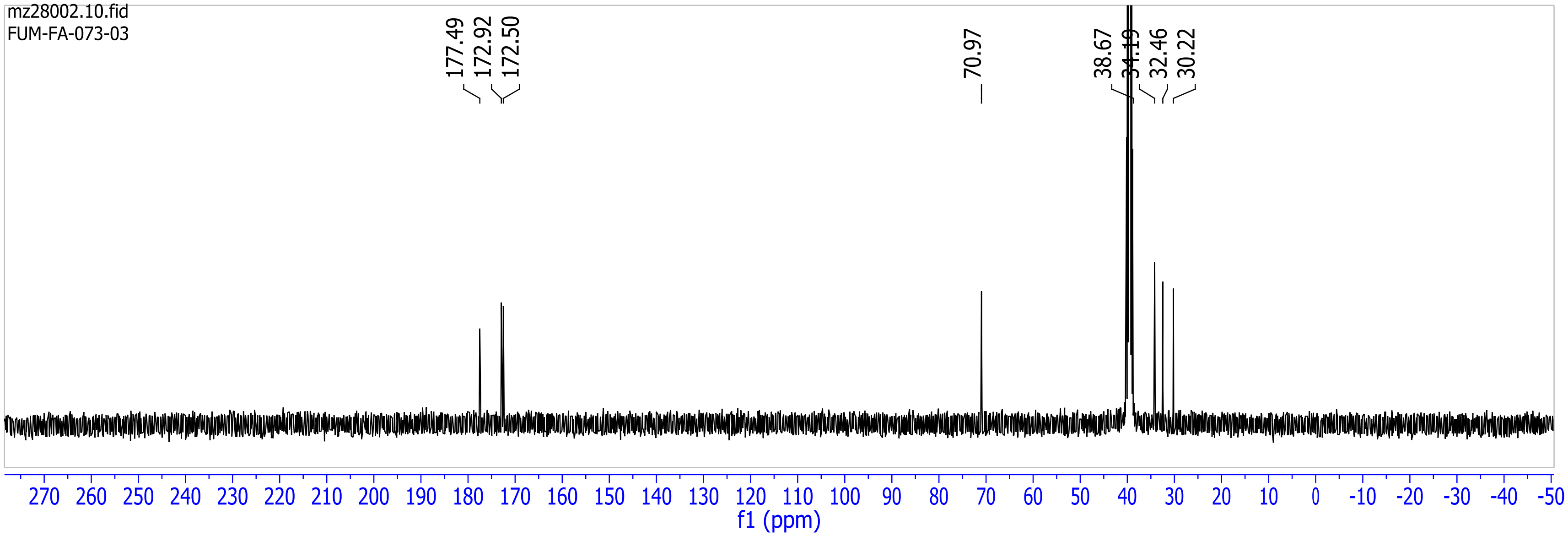
mz28002.10.fid
FUM-FA-073-03
177.49
172.92
172.50
70.97
38.67
34.19
32.46
30.22
270 260 250 240 230 220 210 200 190 180 170 160 150 140 130 120 110 100 90 80 70 60 50 40 30 20 10 0 -10 -20 -30 -40 -50
f1 (ppm)

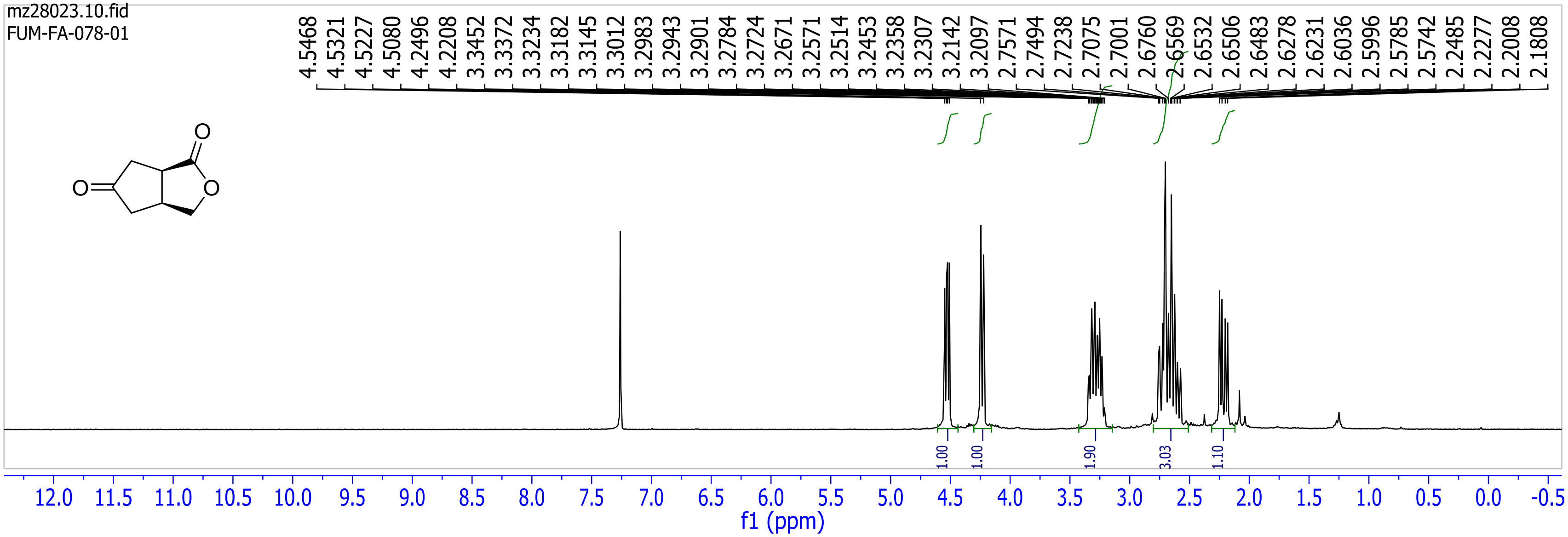
mz28023.10.fid
FUM-FA-078-01
4.5468 4.5321 4.5227 4.5080 4.2496 4.2208 3.3452 3.3372 3.3234 3.3182 3.3145 3.3012 3.2983 3.2943 3.2901 3.2784 3.2724 3.2671 3.2571 3.2514 3.2453 3.2358 3.2307 3.2142 3.2097 2.7571 2.7494 2.7238 2.7075 2.7001 2.6760 2.6569 2.6532 2.6506 2.6483 2.6278 2.6231 2.6036 2.5996 2.5785 2.5742 2.2485 2.2277 2.2008 2.1808
1.00
1.00
1.90
3.03
1.10
f1 (ppm)

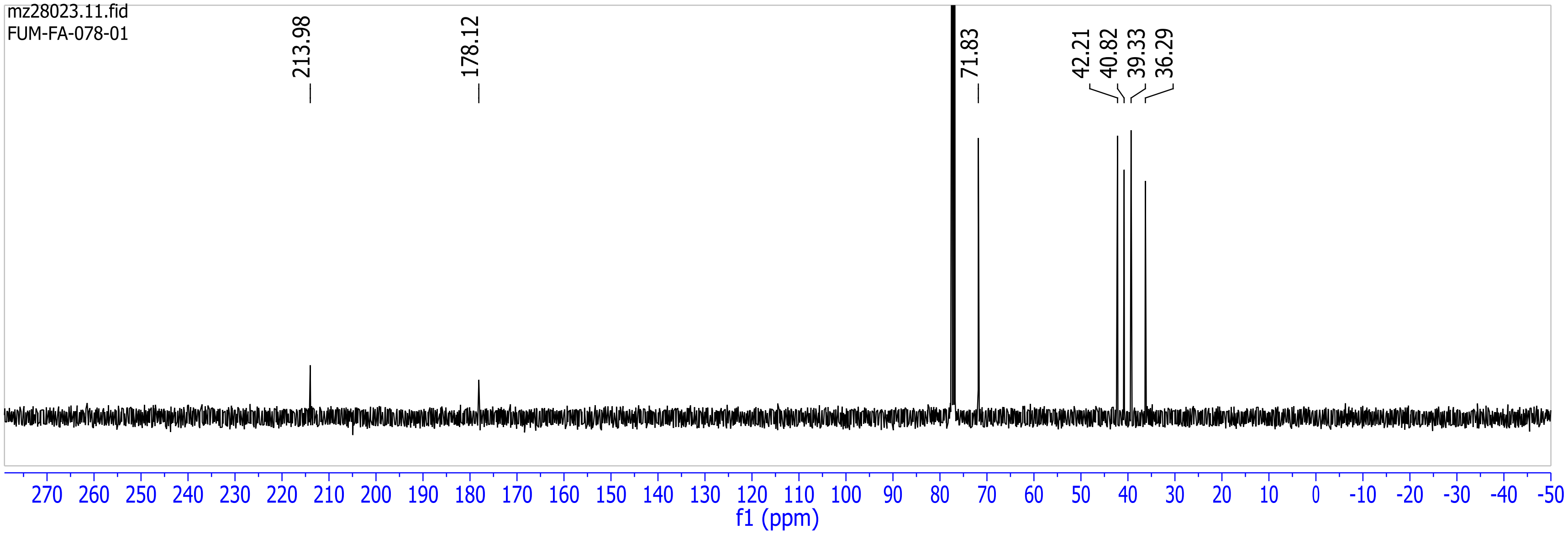
mz28023.11.fid
FUM-FA-078-01
213.98
178.12
71.83
42.21
40.82
39.33
36.29
f1 (ppm)

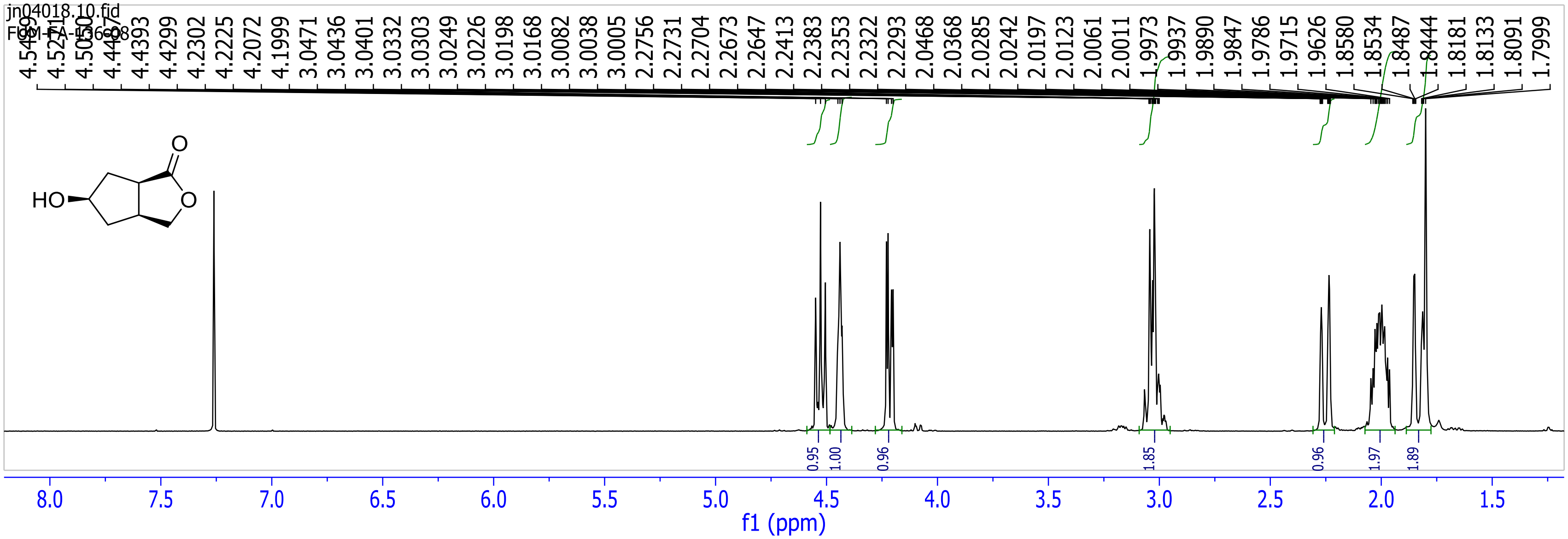
jn04018.10.fid
FUM-FA-136-08
HO
O
O
f1 (ppm)

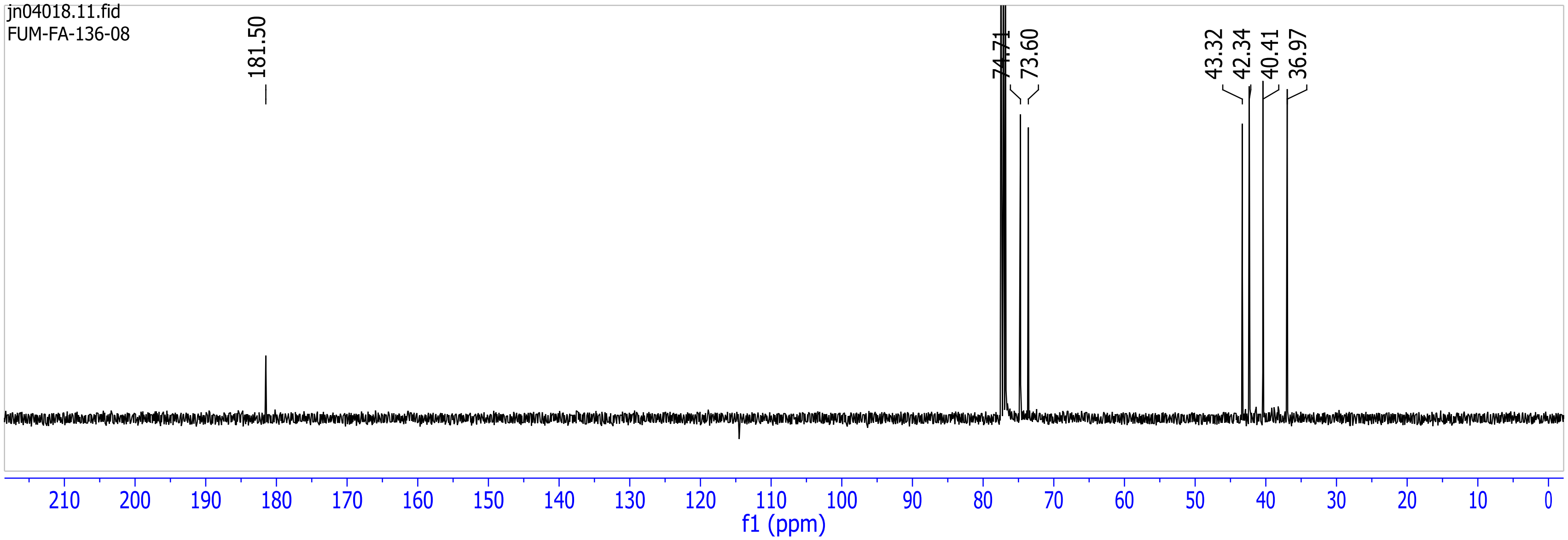
jn04018.11.fid
FUM-FA-136-08
181.50
74.71
73.60
43.32
42.34
40.41
36.97
f1 (ppm)

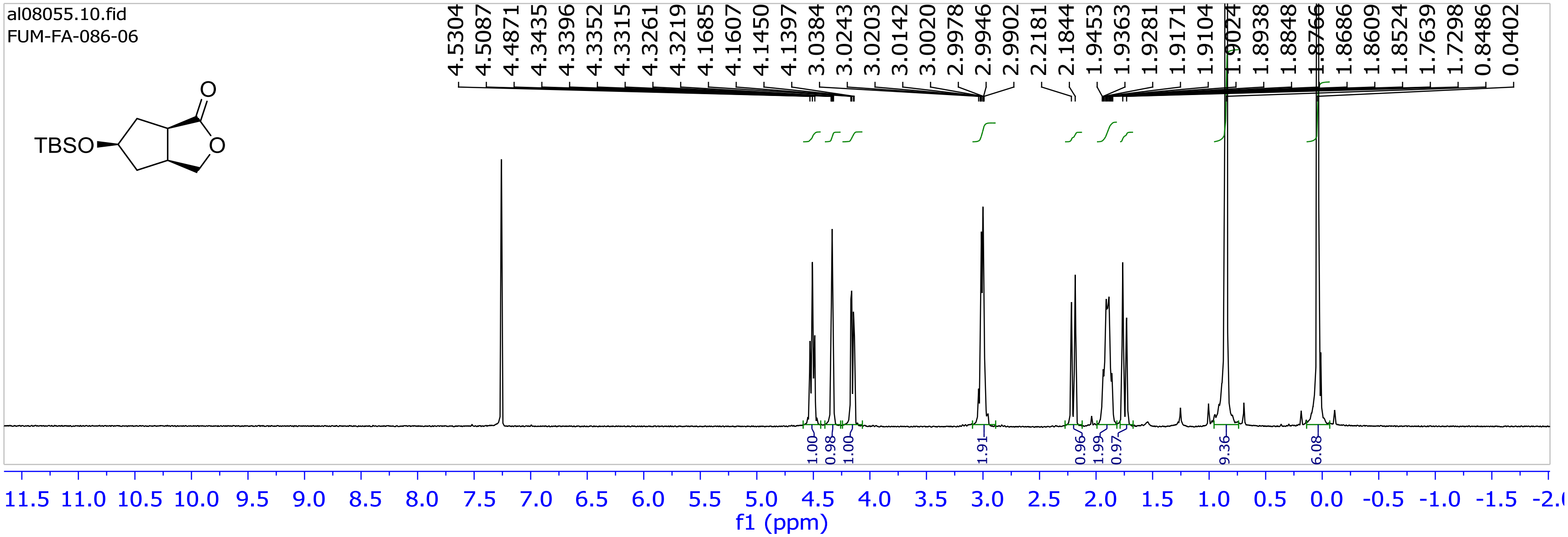
al08055.10.fid
FUM-FA-086-06
TBSO
O
O
4.5304
4.5087
4.4871
4.3435
4.3396
4.3352
4.3315
4.3261
4.3219
4.1685
4.1607
4.1450
4.1397
3.0384
3.0243
3.0203
3.0142
3.0020
2.9978
2.9946
2.9902
2.2181
2.1844
1.9453
1.9363
1.9281
1.9171
1.9104
1.9024
1.8938
1.8848
1.8766
1.8686
1.8609
1.8524
1.7639
1.7298
0.8486
0.0402
1.00
0.98
1.00
1.91
0.96
1.99
0.97
9.36
6.08
f1 (ppm)

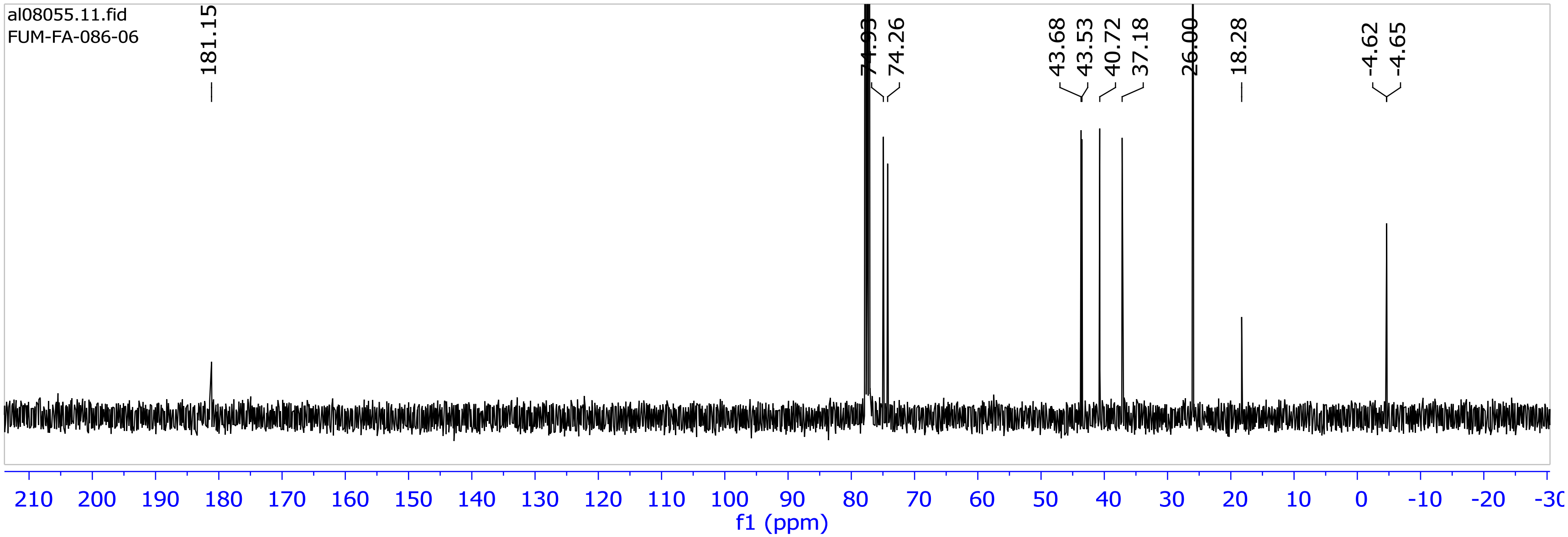
al08055.11.fid
FUM-FA-086-06
181.15
74.26
43.68
43.53
40.72
37.18
26.00
18.28
-4.62
-4.65
f1 (ppm)

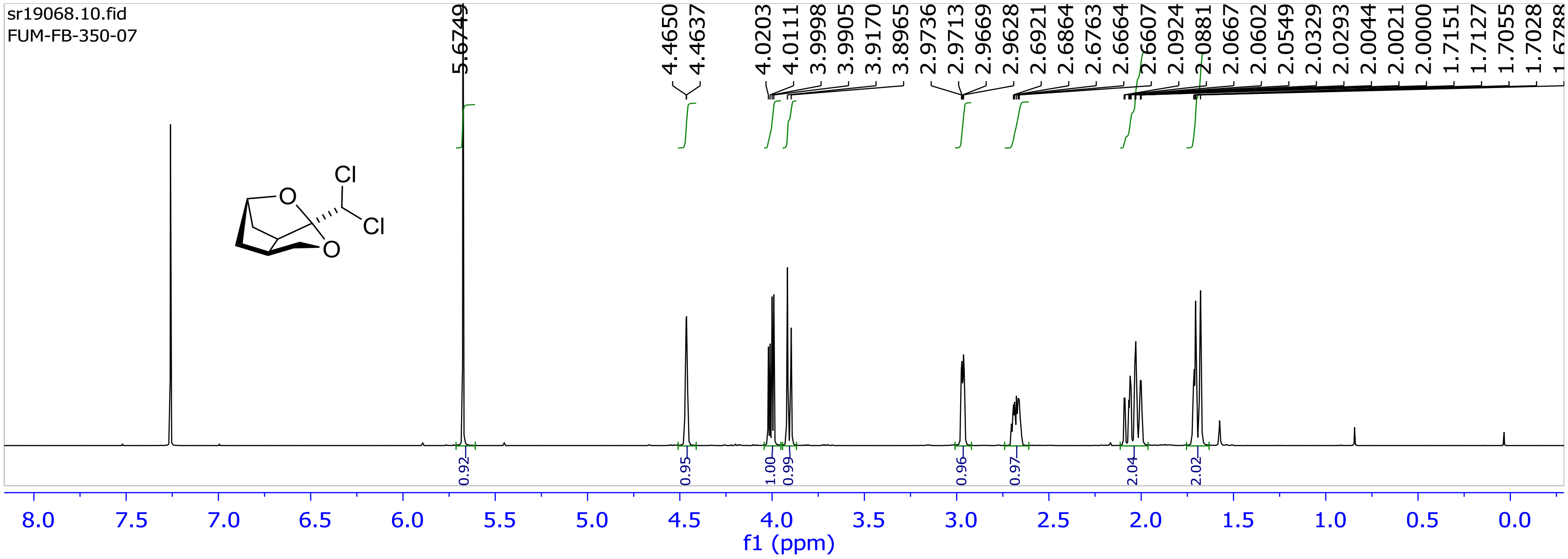

sr19068.10.fid
FUM-FB-350-07
5.6749
4.4650
4.4637
4.0203
4.0111
3.9998
3.9905
3.9170
3.8965
2.9736
2.9713
2.9669
2.9628
2.6921
2.6864
2.6763
2.6664
2.6607
2.0924
2.0881
2.0667
2.0602
2.0549
2.0329
2.0293
2.0044
2.0021
2.0000
1.7151
1.7127
1.7055
1.7028
Cl
Cl
O
O
0.92
0.95
1.00
0.99
0.96
0.97
2.04
2.02
8.0 7.5 7.0 6.5 6.0 5.5 5.0 4.5 4.0 3.5 3.0 2.5 2.0 1.5 1.0 0.5 0.0
f1 (ppm)

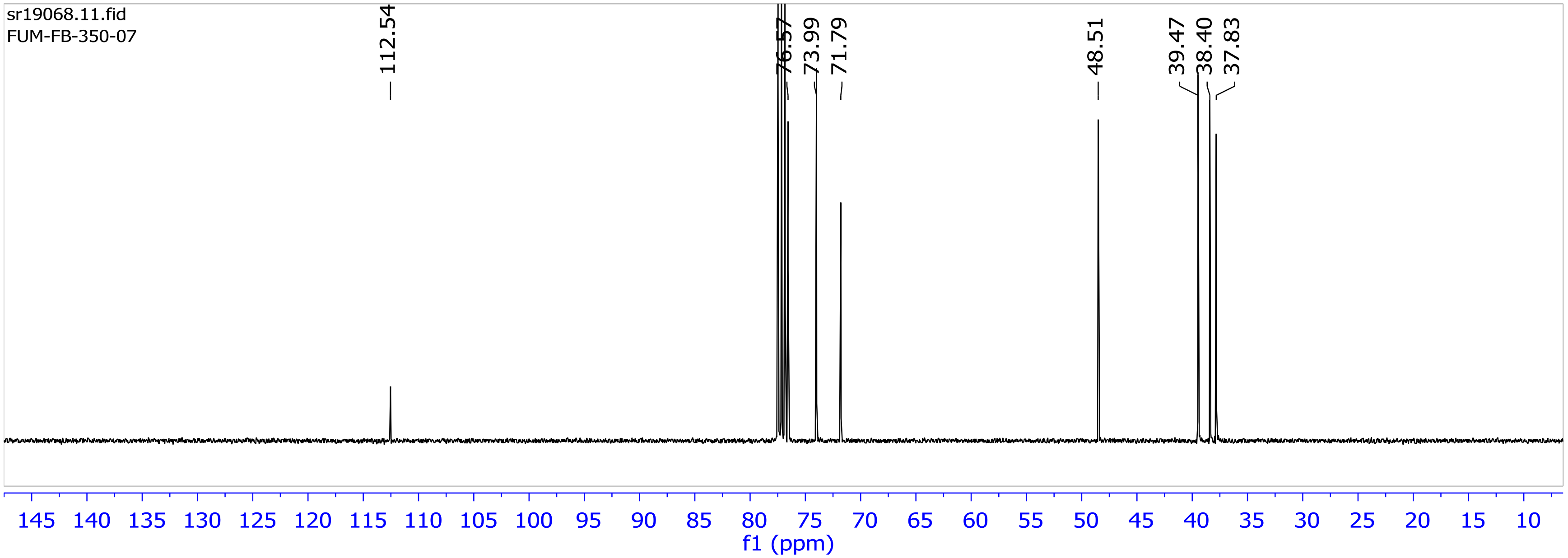

sr19068.11.fid
FUM-FB-350-07
112.54
76.57
73.99
71.79
48.51
39.47
38.40
37.83
145 140 135 130 125 120 115 110 105 100 95 90 85 80 75 70 65 60 55 50 45 40 35 30 25 20 15 10
f1 (ppm)

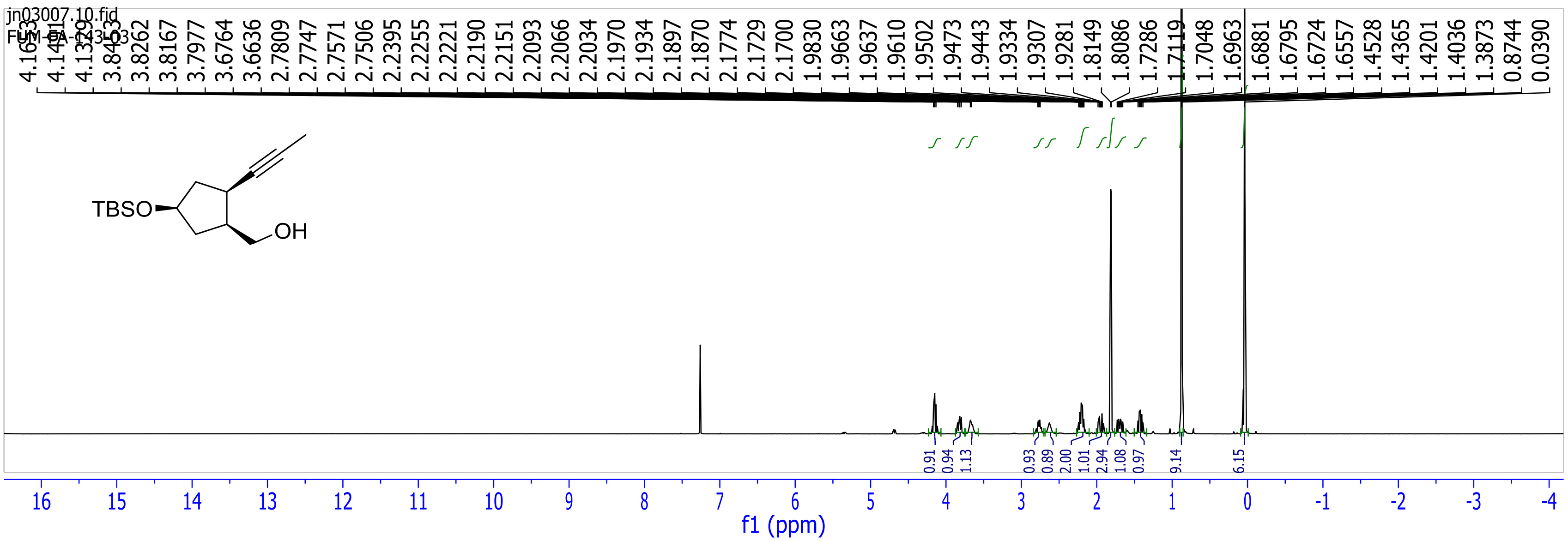
jn03007.10.fid
FUM-FA-143-03
4.1633
4.1491
4.1339
3.8413
3.8262
3.8167
3.7977
3.6764
3.6636
2.7809
2.7747
2.7571
2.7506
2.2395
2.2255
2.2221
2.2190
2.2151
2.2093
2.2066
2.2034
2.1970
2.1934
2.1897
2.1870
2.1774
2.1729
2.1700
1.9830
1.9663
1.9637
1.9610
1.9502
1.9473
1.9443
1.9334
1.9307
1.9281
1.8149
1.8086
1.7286
1.7119
1.7048
1.6963
1.6881
1.6795
1.6724
1.6557
1.4528
1.4365
1.4201
1.4036
1.3873
0.8744
0.0390
TBSO
OH
0.91
0.94
1.13
0.93
0.89
2.00
1.01
2.94
1.08
0.97
9.14
6.15
f1 (ppm)

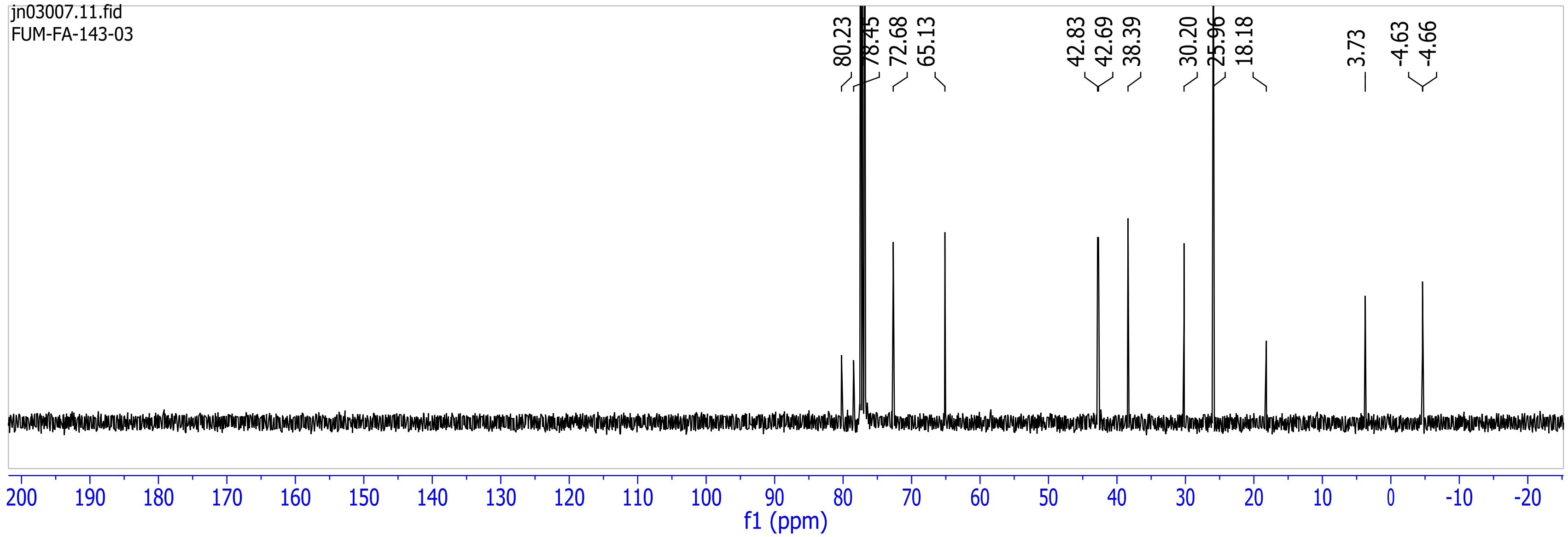
jn03007.11.fid
FUM-FA-143-03
80.23
78.45
72.68
65.13
42.83
42.69
38.39
30.20
25.96
18.18
3.73
-4.63
-4.66
f1 (ppm)

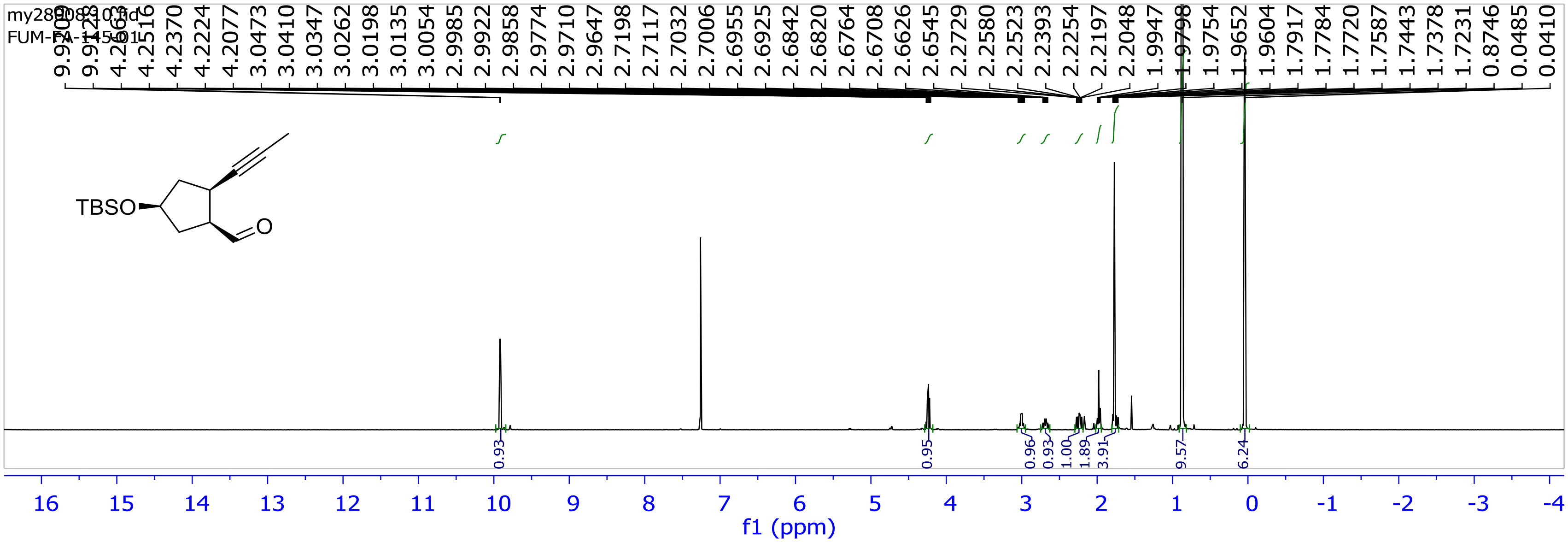
FUM-FA-145-01
TBSO
O
4.2370 4.2224 4.2077 3.0473 3.0410 3.0347 3.0262 3.0198 3.0135 3.0054 2.9985 2.9922 2.9858 2.9774 2.9710 2.9647 2.7198 2.7117 2.7032 2.7006 2.6955 2.6925 2.6842 2.6820 2.6764 2.6708 2.6626 2.6545 2.2729 2.2580 2.2523 2.2393 2.2254 2.2197 2.2048 1.9947 1.9798 1.9754 1.9652 1.9604 1.7917 1.7784 1.7720 1.7587 1.7443 1.7378 1.7231 0.8746 0.0485 0.0410
0.93 0.95 0.96 0.93 1.00 1.89 3.91 9.57 6.24
16 15 14 13 12 11 10 9 8 7 6 5 4 3 2 1 0 -1 -2 -3 -4
f1 (ppm)

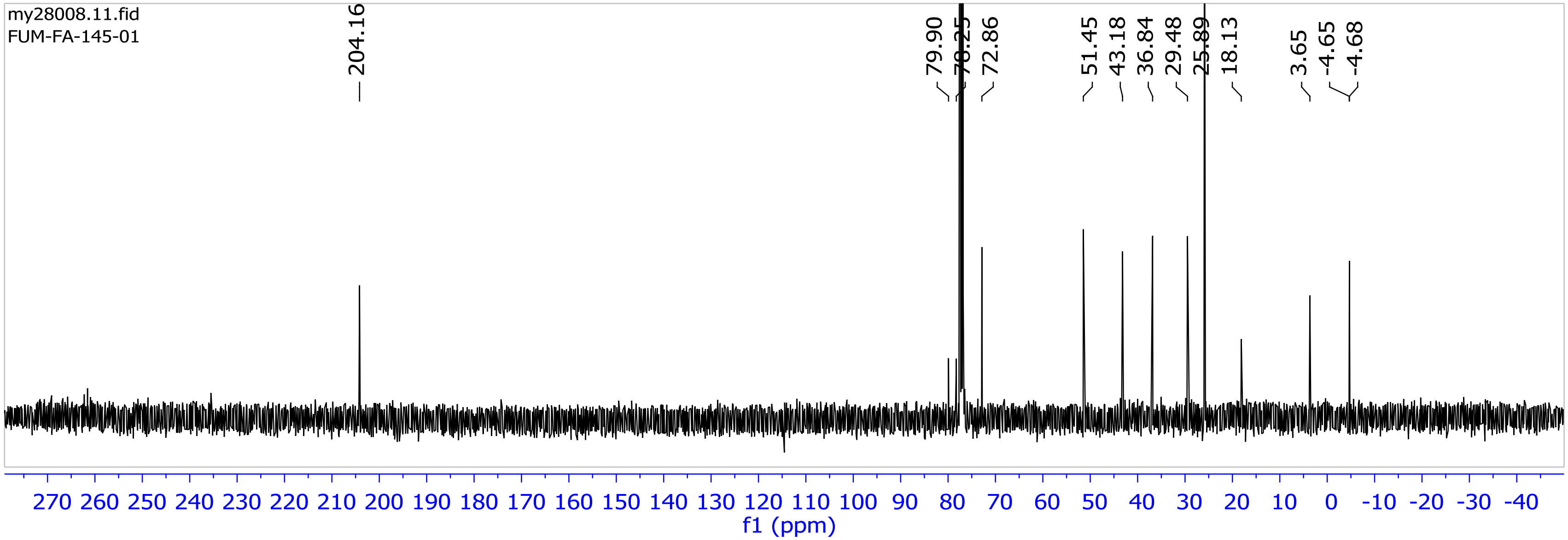
my28008.11.fid
FUM-FA-145-01
204.16
79.90
78.25
72.86
51.45
43.18
36.84
29.48
25.89
18.13
3.65
-4.65
-4.68
270 260 250 240 230 220 210 200 190 180 170 160 150 140 130 120 110 100 90 80 70 60 50 40 30 20 10 0 -10 -20 -30 -40
f1 (ppm)

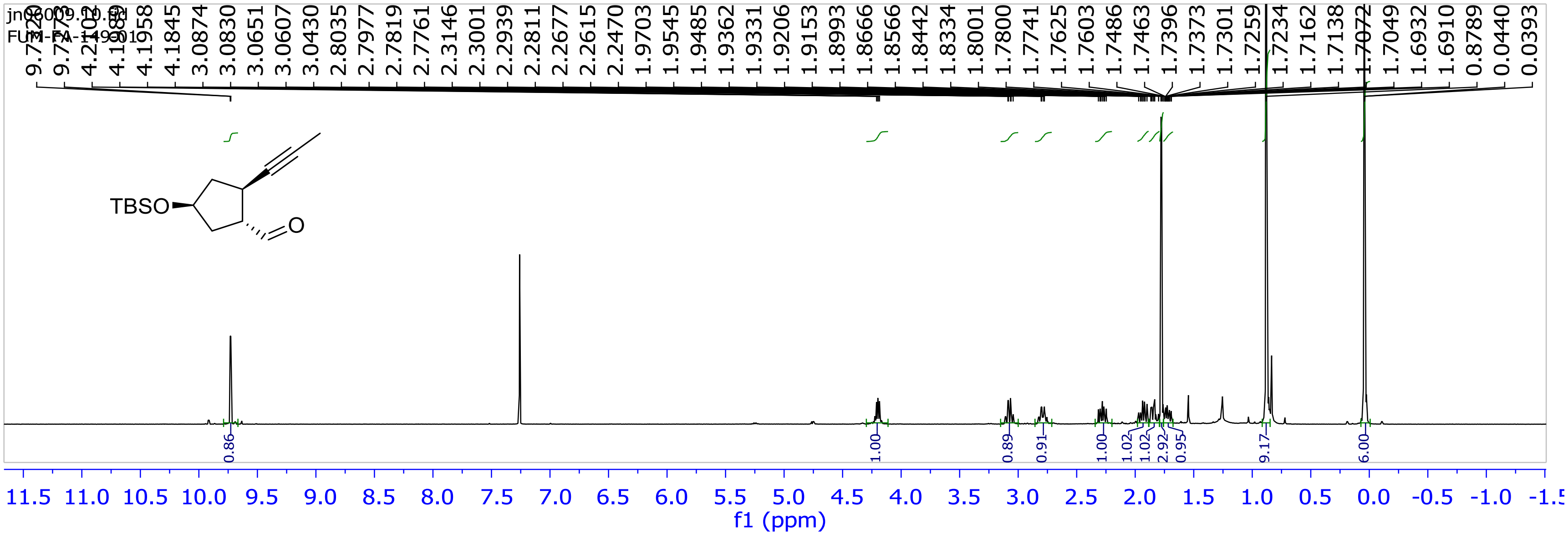
FUM-FA-149-01
4.1958
4.1845
3.0874
3.0830
3.0651
3.0607
3.0430
2.8035
2.7977
2.7819
2.7761
2.3146
2.3001
2.2939
2.2811
2.2677
2.2615
2.2470
1.9703
1.9545
1.9485
1.9362
1.9331
1.9206
1.9153
1.8993
1.8666
1.8566
1.8442
1.8334
1.8001
1.7800
1.7741
1.7625
1.7603
1.7486
1.7463
1.7396
1.7373
1.7301
1.7259
1.7234
1.7162
1.7138
1.7072
1.7049
1.6932
1.6910
0.8789
0.0440
0.0393
TBSO
O
0.86
1.00
0.89
0.91
1.00
1.02
1.02
2.92
0.95
9.17
6.00
f1 (ppm)
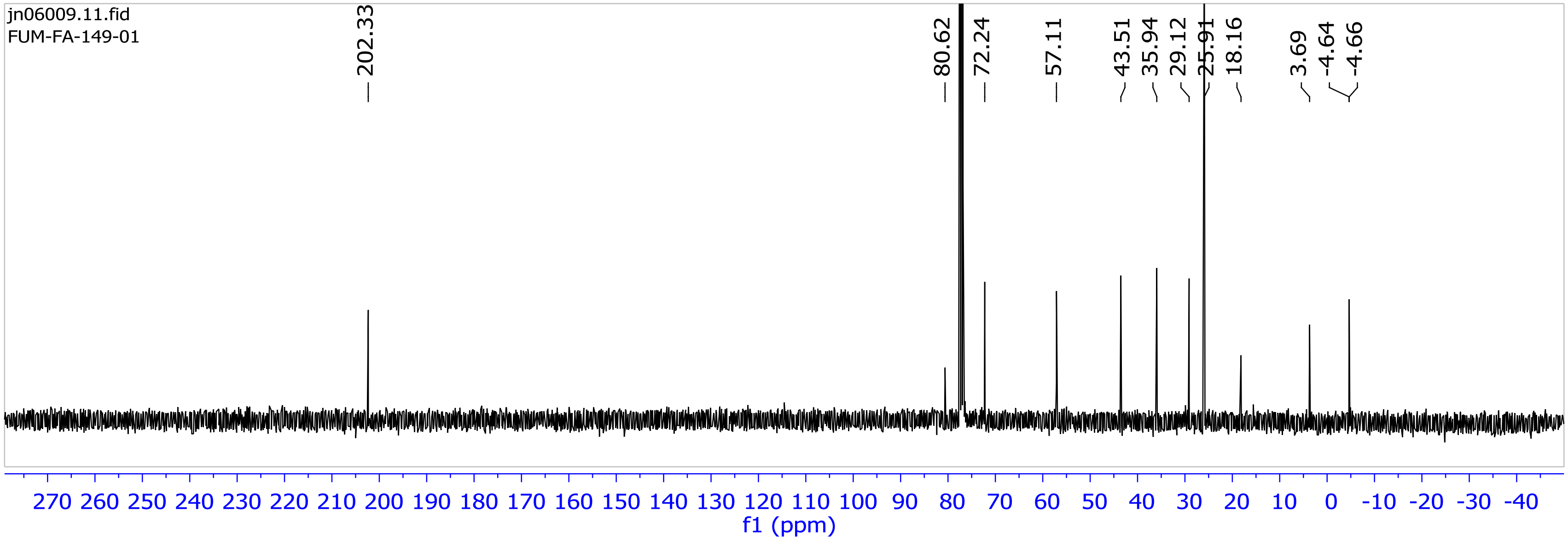
jn06009.11.fid
FUM-FA-149-01
202.33
80.62
72.24
57.11
43.51
35.94
29.12
25.91
18.16
3.69
-4.64
-4.66
f1 (ppm)

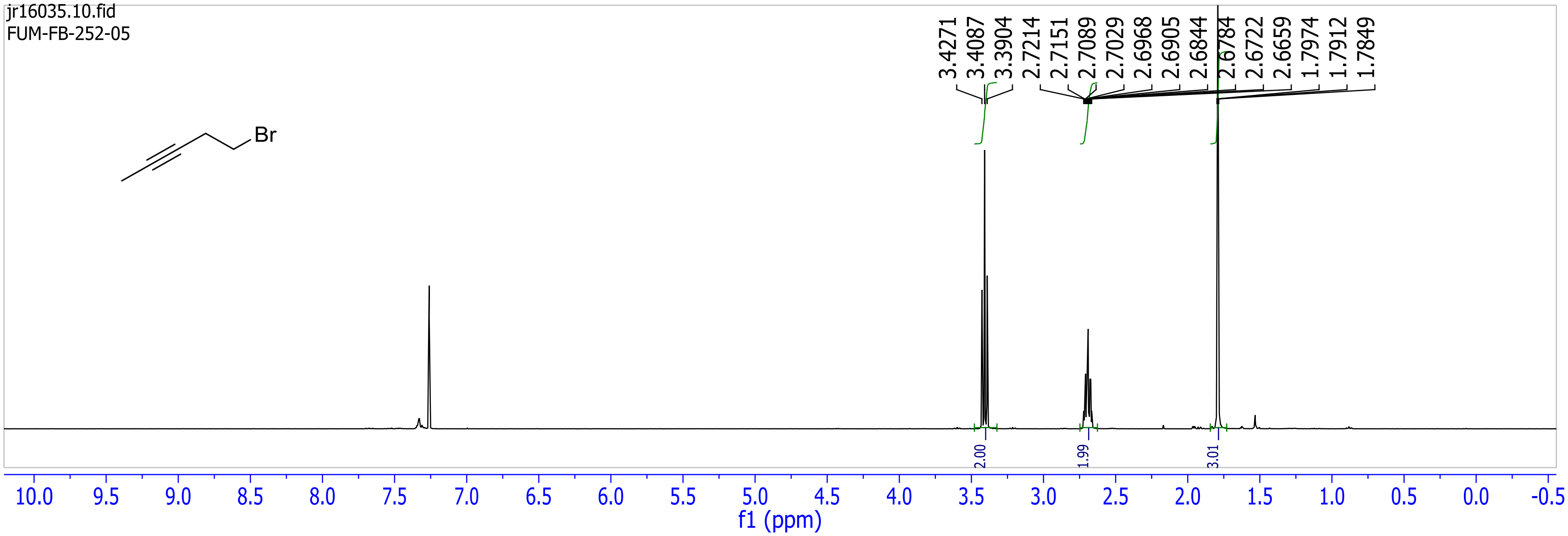
jr16035.10.fid
FUM-FB-252-05
Br
3.4271
3.4087
3.3904
2.7214
2.7151
2.7089
2.7029
2.6968
2.6905
2.6844
2.6784
2.6722
2.6659
1.7974
1.7912
1.7849
2.00
1.99
3.01
f1 (ppm)

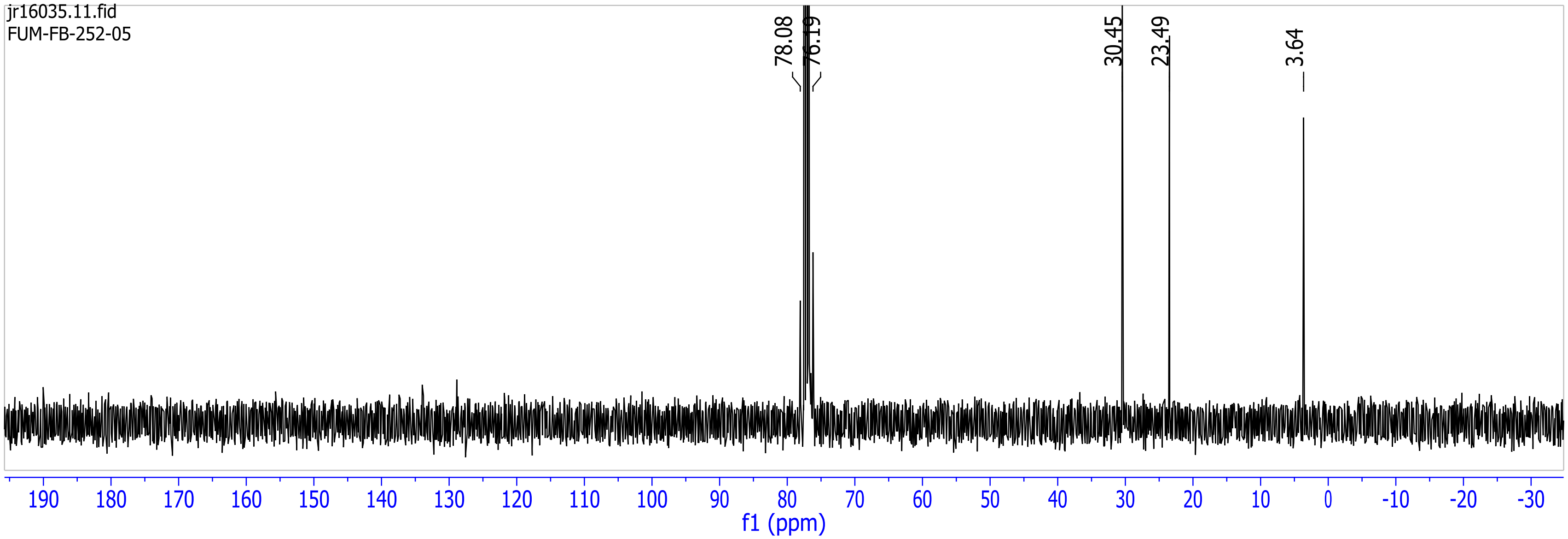
jr16035.11.fid
FUM-FB-252-05
78.08
76.19
30.45
23.49
3.64
f1 (ppm)

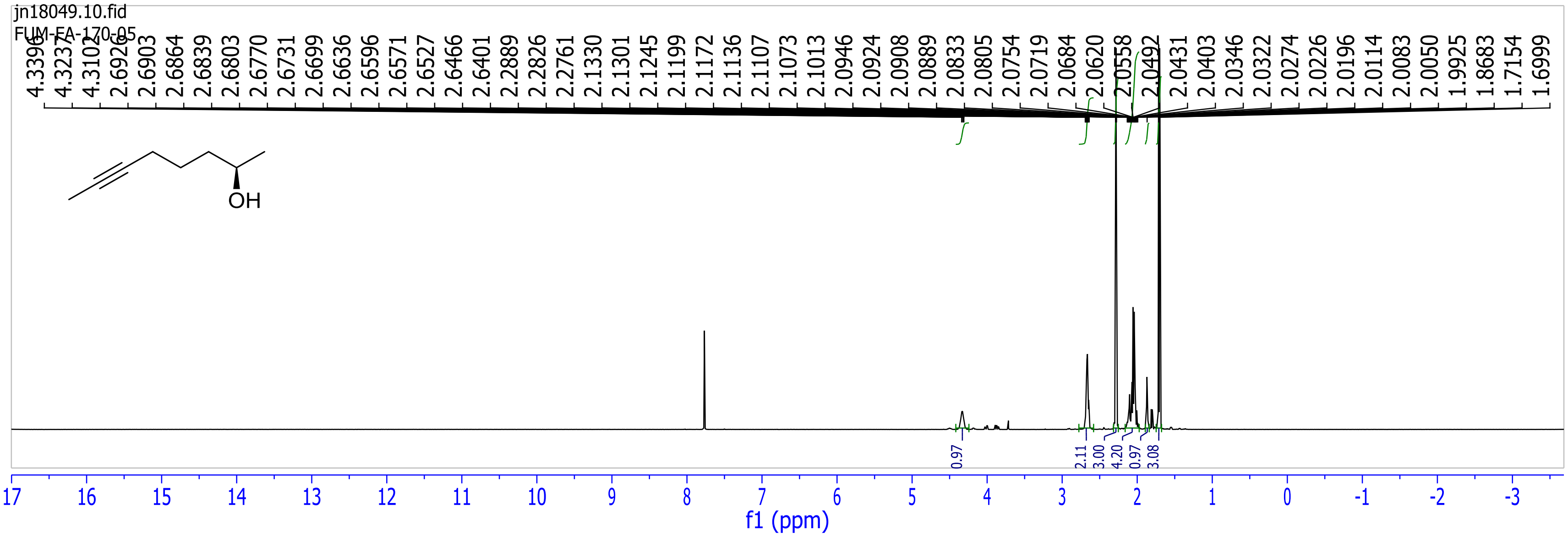
jn18049.10.fid
FUM-FA-170-05
4.3390 4.3237 4.3102 2.6920 2.6903 2.6864 2.6839 2.6803 2.6770 2.6731 2.6699 2.6636 2.6596 2.6571 2.6527 2.6466 2.6401 2.2889 2.2826 2.2761 2.1330 2.1301 2.1245 2.1199 2.1172 2.1136 2.1107 2.1073 2.1013 2.0946 2.0924 2.0908 2.0889 2.0833 2.0805 2.0754 2.0719 2.0684 2.0620 2.0558 2.0492 2.0431 2.0403 2.0346 2.0322 2.0274 2.0226 2.0196 2.0114 2.0083 2.0050 1.9925 1.8683 1.7154 1.6999
OH
0.97
2.11
3.00
4.20
0.97
3.08
17 16 15 14 13 12 11 10 9 8 7 6 5 4 3 2 1 0 -1 -2 -3
f1 (ppm)

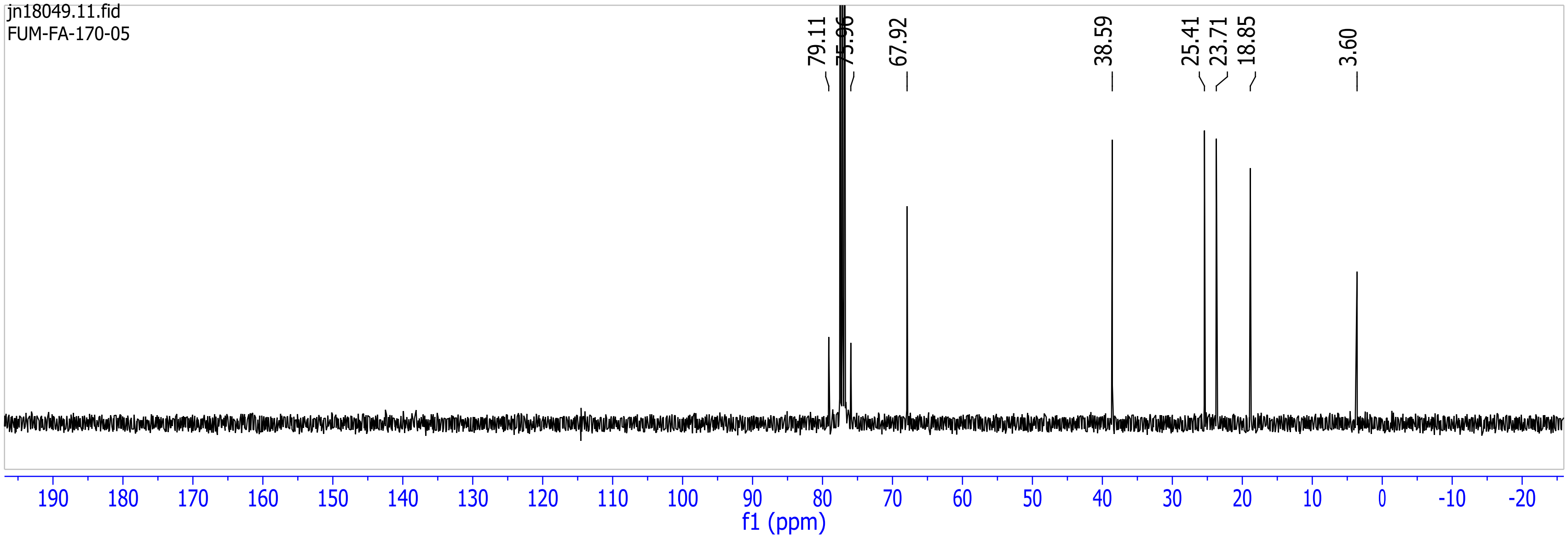
jn18049.11.fid
FUM-FA-170-05
79.11
75.96
67.92
38.59
25.41
23.71
18.85
3.60
190 180 170 160 150 140 130 120 110 100 90 80 70 60 50 40 30 20 10 0 -10 -20
f1 (ppm)

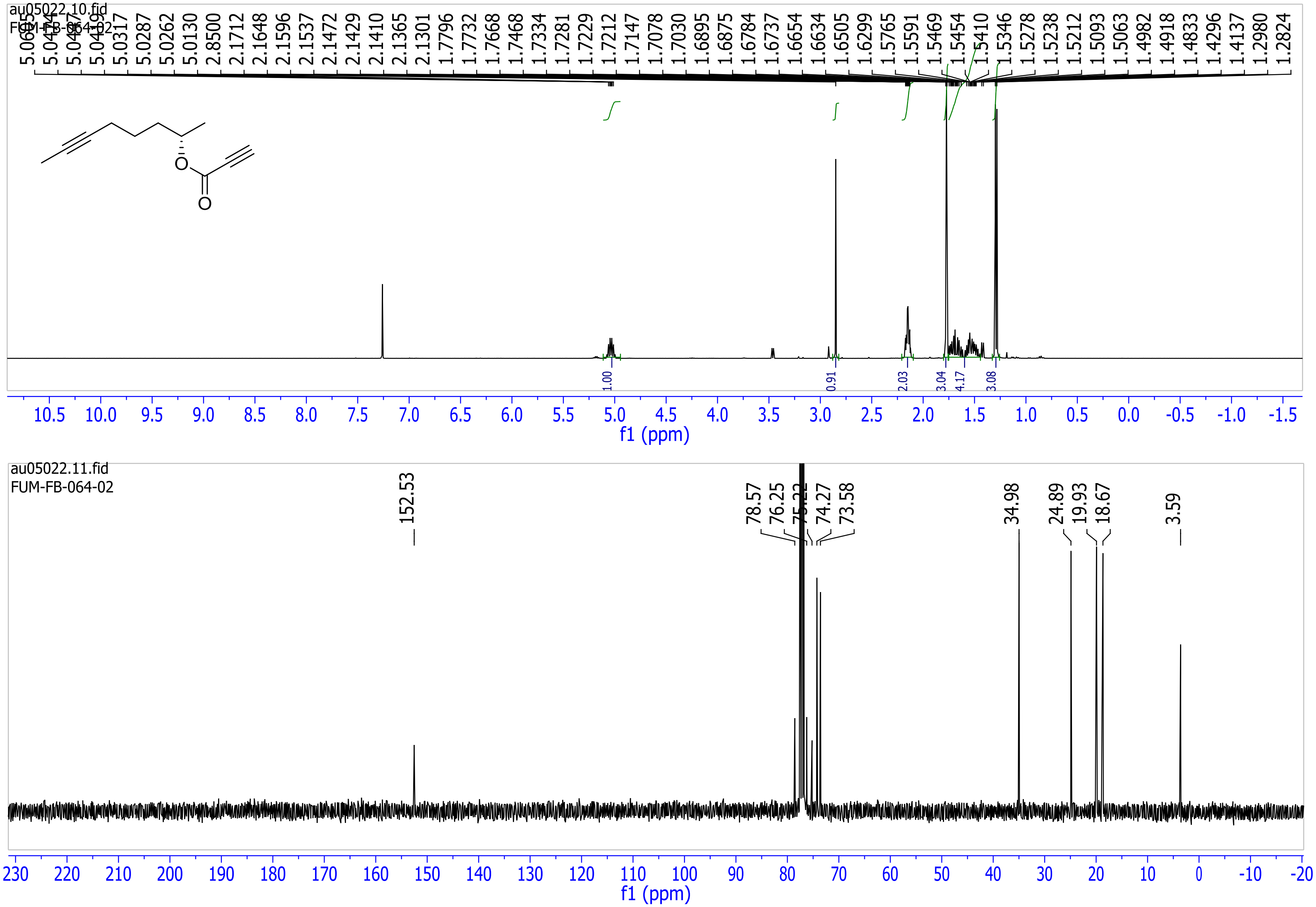
au05022.10.fid
FUM-FB-064-02
5.0317
5.0287
5.0262
5.0130
2.8500
2.1712
2.1648
2.1596
2.1537
2.1472
2.1429
2.1410
2.1365
2.1301
1.7796
1.7732
1.7668
1.7468
1.7334
1.7281
1.7229
1.7212
1.7147
1.7078
1.7030
1.6895
1.6875
1.6784
1.6737
1.6654
1.6634
1.6505
1.6299
1.5765
1.5591
1.5469
1.5454
1.5410
1.5346
1.5278
1.5238
1.5212
1.5093
1.5063
1.4982
1.4918
1.4833
1.4296
1.4137
1.2980
1.2824
O
O
1.00
0.91
2.03
3.04
4.17
3.08
f1 (ppm)
au05022.11.fid
FUM-FB-064-02
152.53
78.57
76.25
74.27
73.58
34.98
24.89
19.93
18.67
3.59
f1 (ppm)

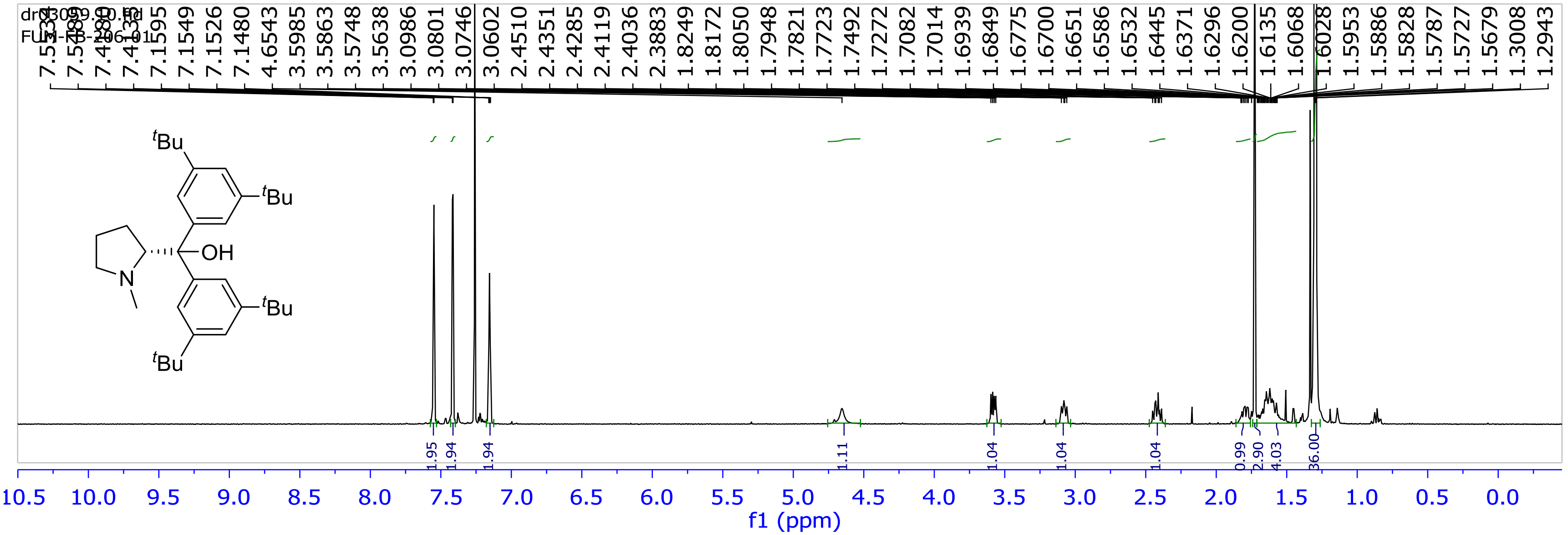
FUM-FB-206-01
7.5[illegible]
7.5[illegible]
7.4[illegible]
7.4[illegible]
7.1595
7.1549
7.1526
7.1480
4.6543
3.5985
3.5863
3.5748
3.5638
3.0986
3.0801
3.0746
3.0602
2.4510
2.4351
2.4285
2.4119
2.4036
2.3883
1.8249
1.8172
1.8050
1.7948
1.7821
1.7723
1.7492
1.7272
1.7082
1.7014
1.6939
1.6849
1.6775
1.6700
1.6651
1.6586
1.6532
1.6445
1.6371
1.6296
1.6200
1.6135
1.6068
1.6028
1.5953
1.5886
1.5828
1.5787
1.5727
1.5679
1.3008
1.2943
^{t}Bu
^{t}Bu
OH
N
^{t}Bu
^{t}Bu
1.95
1.94
1.94
1.11
1.04
1.04
1.04
0.99
2.90
4.03
36.00
10.5 10.0 9.5 9.0 8.5 8.0 7.5 7.0 6.5 6.0 5.5 5.0 4.5 4.0 3.5 3.0 2.5 2.0 1.5 1.0 0.5 0.0
f1 (ppm)

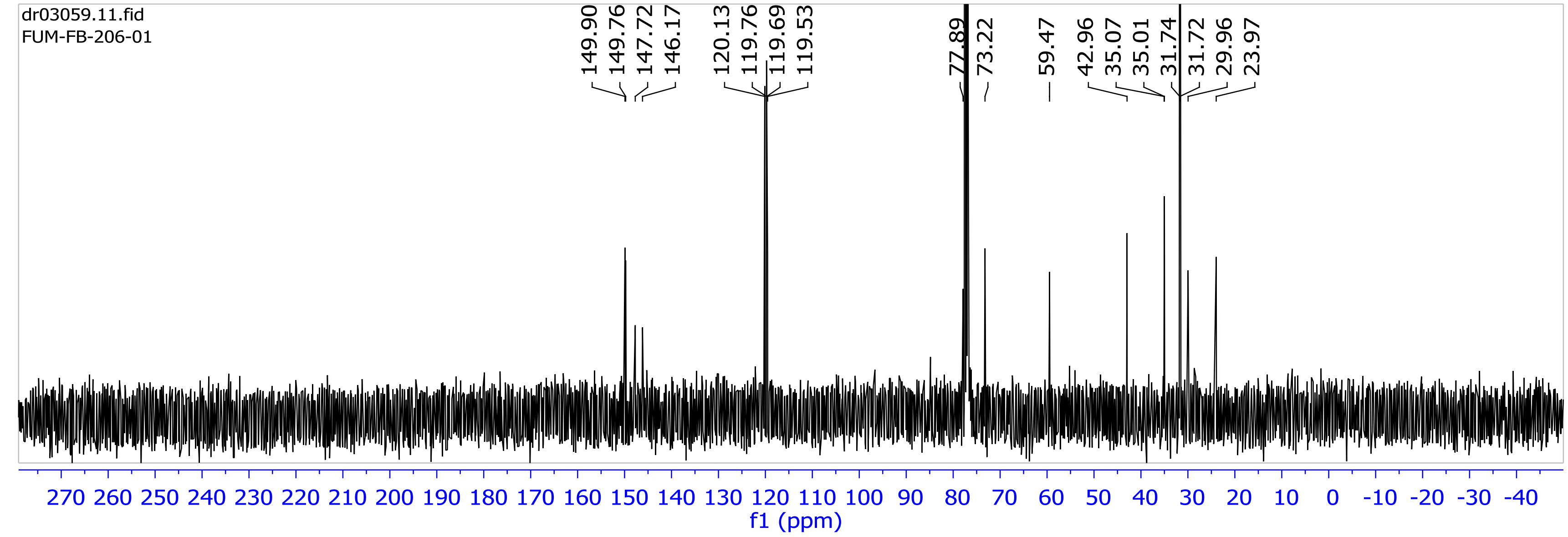
dr03059.11.fid
FUM-FB-206-01
149.90
149.76
147.72
146.17
120.13
119.76
119.69
119.53
77.89
73.22
59.47
42.96
35.07
35.01
31.74
31.72
29.96
23.97
270 260 250 240 230 220 210 200 190 180 170 160 150 140 130 120 110 100 90 80 70 60 50 40 30 20 10 0 -10 -20 -30 -40
f1 (ppm)

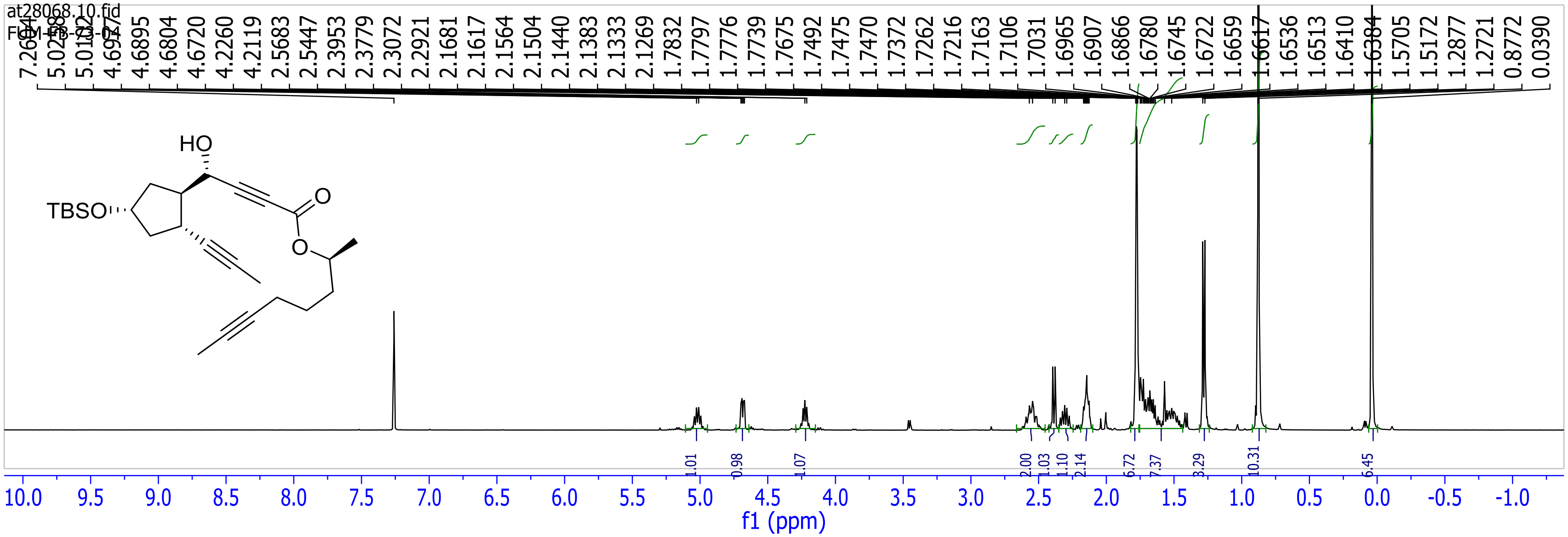

at28068.10.fid
4.6895
4.6804
4.6720
4.2260
4.2119
2.5683
2.5447
2.3953
2.3779
2.3072
2.2921
2.1681
2.1617
2.1564
2.1504
2.1440
2.1383
2.1333
2.1269
1.7832
1.7797
1.7776
1.7739
1.7675
1.7492
1.7475
1.7470
1.7372
1.7262
1.7216
1.7163
1.7106
1.7031
1.6965
1.6907
1.6866
1.6780
1.6745
1.6722
1.6659
1.6617
1.6536
1.6513
1.6410
1.6384
1.5705
1.5172
1.2877
1.2721
0.8772
0.0390
HO
TBSO
O
O
1.01
0.98
1.07
2.00
1.03
1.10
2.14
6.72
7.37
3.29
10.31
6.45
10.0 9.5 9.0 8.5 8.0 7.5 7.0 6.5 6.0 5.5 5.0 4.5 4.0 3.5 3.0 2.5 2.0 1.5 1.0 0.5 0.0 -0.5 -1.0
f1 (ppm)

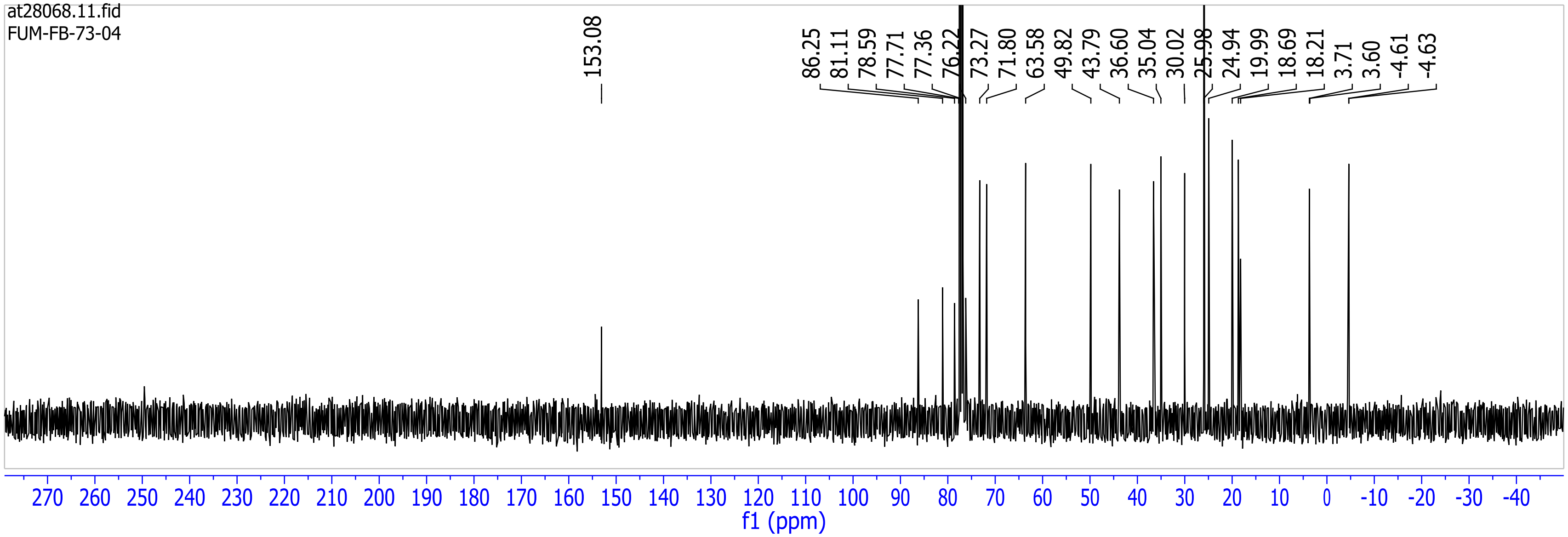

at28068.11.fid
FUM-FB-73-04
153.08
86.25
81.11
78.59
77.71
77.36
76.22
73.27
71.80
63.58
49.82
43.79
36.60
35.04
30.02
25.98
24.94
19.99
18.69
18.21
3.71
3.60
-4.61
-4.63
270 260 250 240 230 220 210 200 190 180 170 160 150 140 130 120 110 100 90 80 70 60 50 40 30 20 10 0 -10 -20 -30 -40
f1 (ppm)

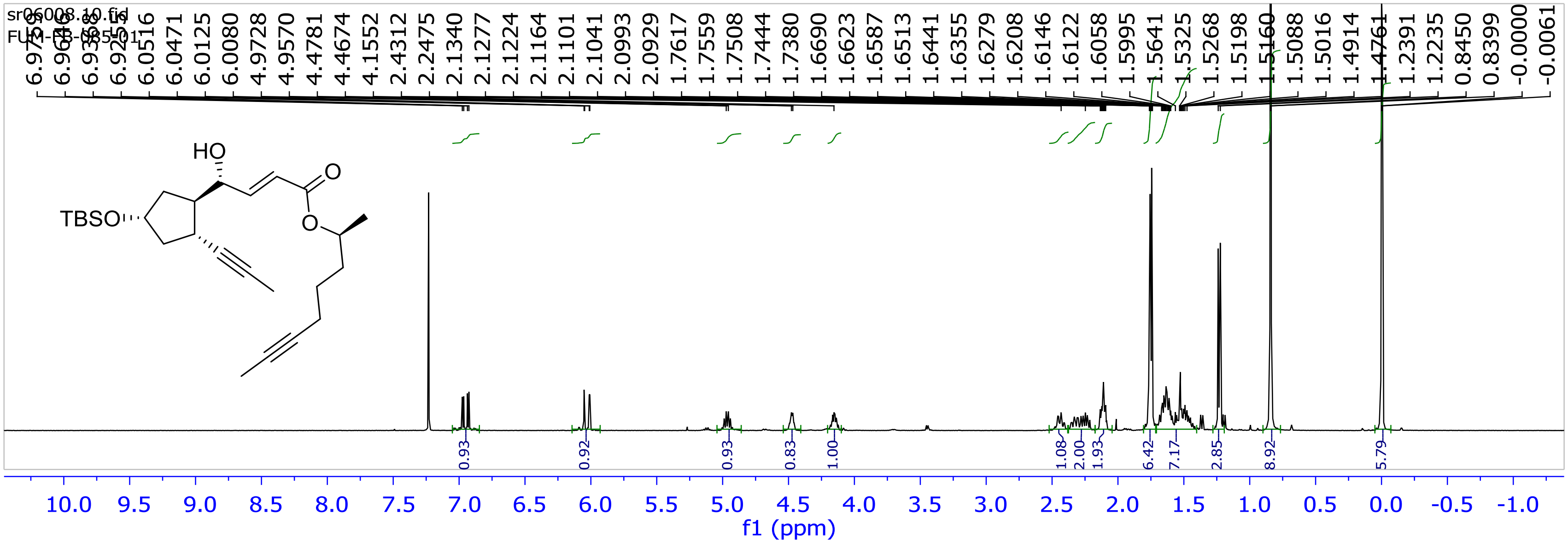
sr06008.10.fid
FUM-FB-085-01
6.0516
6.0471
6.0125
6.0080
4.9728
4.9570
4.4781
4.4674
4.1552
2.4312
2.2475
2.1340
2.1277
2.1224
2.1164
2.1101
2.1041
2.0993
2.0929
1.7617
1.7559
1.7508
1.7444
1.7380
1.6690
1.6623
1.6587
1.6513
1.6441
1.6355
1.6279
1.6208
1.6146
1.6122
1.6058
1.5995
1.5641
1.5325
1.5268
1.5198
1.5160
1.5088
1.5016
1.4914
1.4761
1.2391
1.2235
0.8450
0.8399
-0.0000
-0.0061
HO
O
TBSO
O
0.93
0.92
0.93
0.83
1.00
1.08
2.00
1.93
6.42
7.17
2.85
8.92
5.79
10.0 9.5 9.0 8.5 8.0 7.5 7.0 6.5 6.0 5.5 5.0 4.5 4.0 3.5 3.0 2.5 2.0 1.5 1.0 0.5 0.0 -0.5 -1.0
f1 (ppm)

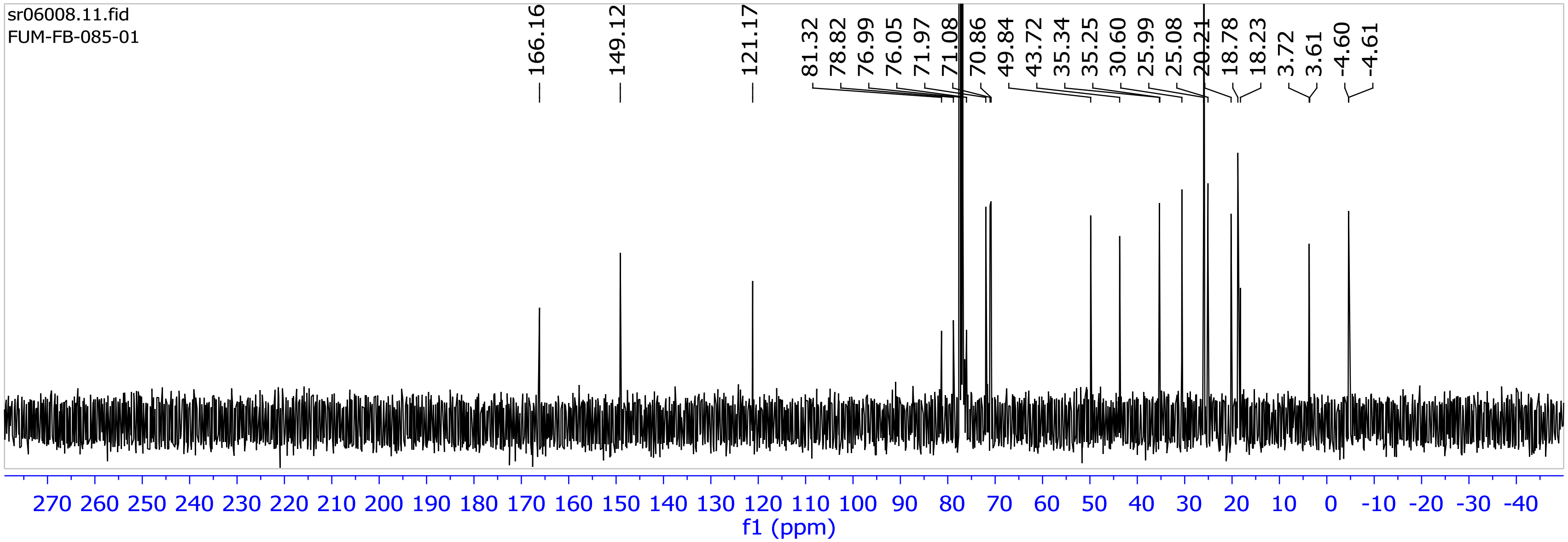
sr06008.11.fid
FUM-FB-085-01
166.16
149.12
121.17
81.32
78.82
76.99
76.05
71.97
71.08
70.86
49.84
43.72
35.34
35.25
30.60
25.99
25.08
20.21
18.78
18.23
3.72
3.61
-4.60
-4.61
270 260 250 240 230 220 210 200 190 180 170 160 150 140 130 120 110 100 90 80 70 60 50 40 30 20 10 0 -10 -20 -30 -40
f1 (ppm)

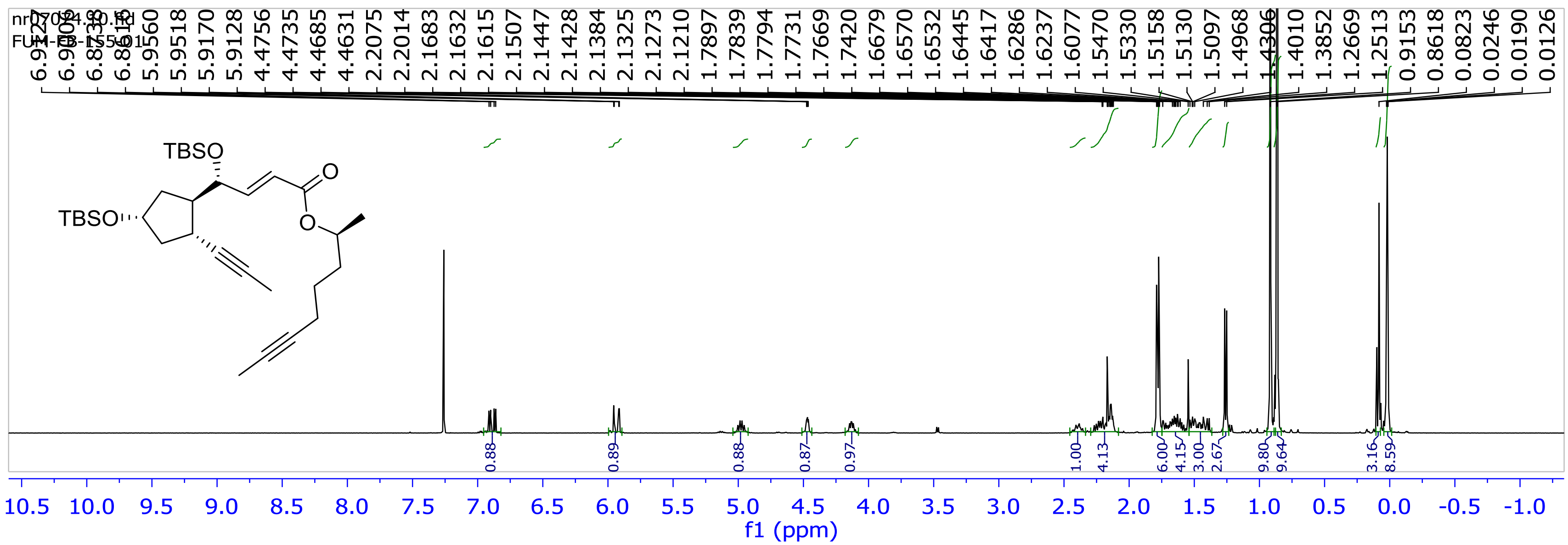
FUM-FB-155-01
5.9560
5.9518
5.9170
5.9128
4.4756
4.4735
4.4685
4.4631
2.2075
2.2014
2.1683
2.1632
2.1615
2.1507
2.1447
2.1428
2.1384
2.1325
2.1273
2.1210
1.7897
1.7839
1.7794
1.7731
1.7669
1.7420
1.6679
1.6570
1.6532
1.6445
1.6417
1.6286
1.6237
1.6077
1.5470
1.5330
1.5158
1.5130
1.5097
1.4968
1.4010
1.3852
1.2669
1.2513
0.9153
0.8618
0.0823
0.0246
0.0190
0.0126
TBSO
TBSO
O
O
0.88
0.89
0.88
0.87
0.97
1.00
4.13
6.00
4.15
3.00
2.67
9.80
9.64
3.16
8.59
f1 (ppm)

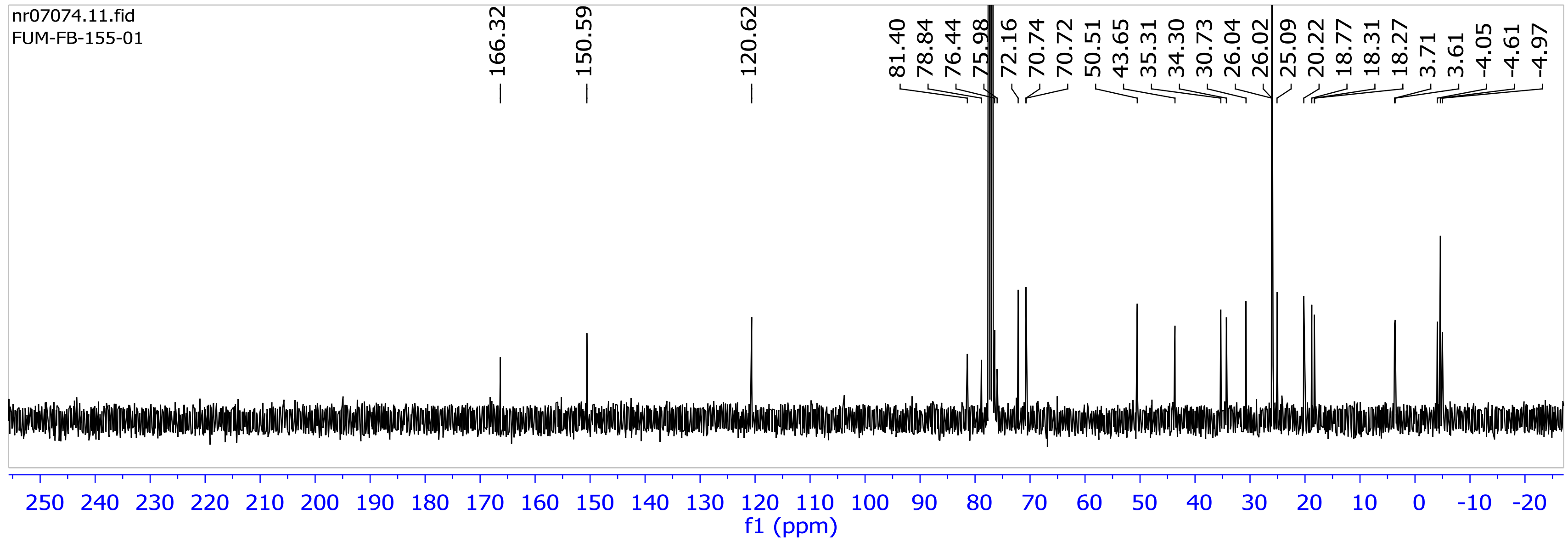
nr07074.11.fid
FUM-FB-155-01
166.32
150.59
120.62
81.40
78.84
76.44
75.98
72.16
70.74
70.72
50.51
43.65
35.31
34.30
30.73
26.04
26.02
25.09
20.22
18.77
18.31
18.27
3.71
3.61
-4.05
-4.61
-4.97
f1 (ppm)

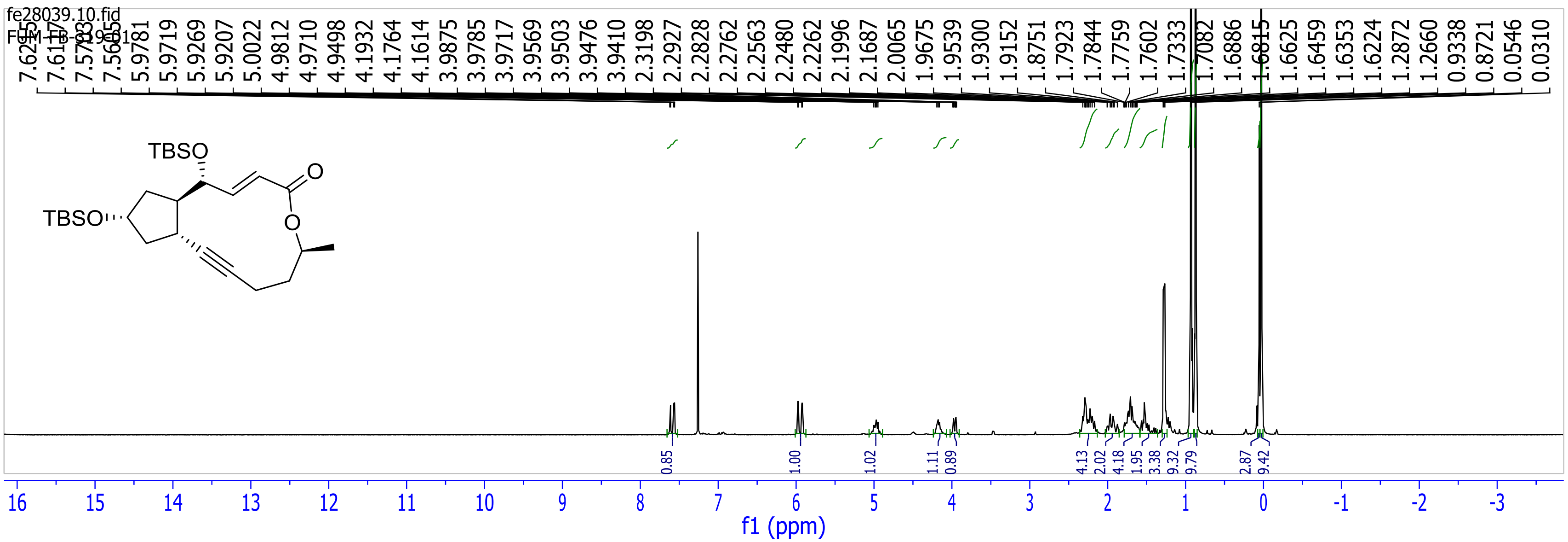
fe28039.10.fid
FUM-TB-319-01
TBSO
TBSO
O
O
7.6257 7.6117 7.5733 7.5605 5.9781 5.9719 5.9269 5.9207 5.0022 4.9812 4.9710 4.9498 4.1932 4.1764 4.1614 3.9875 3.9785 3.9717 3.9569 3.9503 3.9476 3.9410 2.3198 2.2927 2.2828 2.2762 2.2563 2.2480 2.2262 2.1996 2.1687 2.0065 1.9675 1.9539 1.9300 1.9152 1.8751 1.7923 1.7844 1.7759 1.7602 1.7333 1.7082 1.6886 1.6815 1.6625 1.6459 1.6353 1.6224 1.2872 1.2660 0.9338 0.8721 0.0546 0.0310
0.85 1.00 1.02 1.11 0.89 4.13 2.02 4.18 1.95 3.38 9.32 9.79 2.87 9.42
16 15 14 13 12 11 10 9 8 7 6 5 4 3 2 1 0 -1 -2 -3
f1 (ppm)

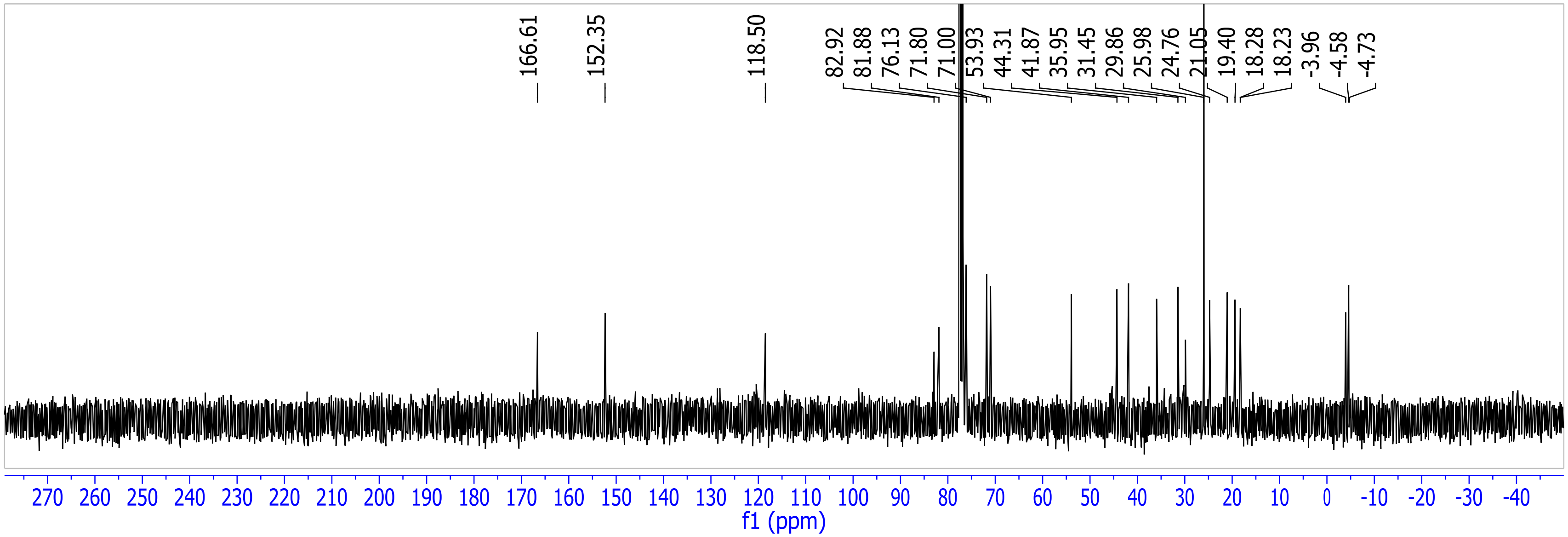
166.61 152.35 118.50 82.92 81.88 76.13 71.80 71.00 53.93 44.31 41.87 35.95 31.45 29.86 25.98 24.76 21.05 19.40 18.28 18.23 -3.96 -4.58 -4.73
270 260 250 240 230 220 210 200 190 180 170 160 150 140 130 120 110 100 90 80 70 60 50 40 30 20 10 0 -10 -20 -30 -40
f1 (ppm)

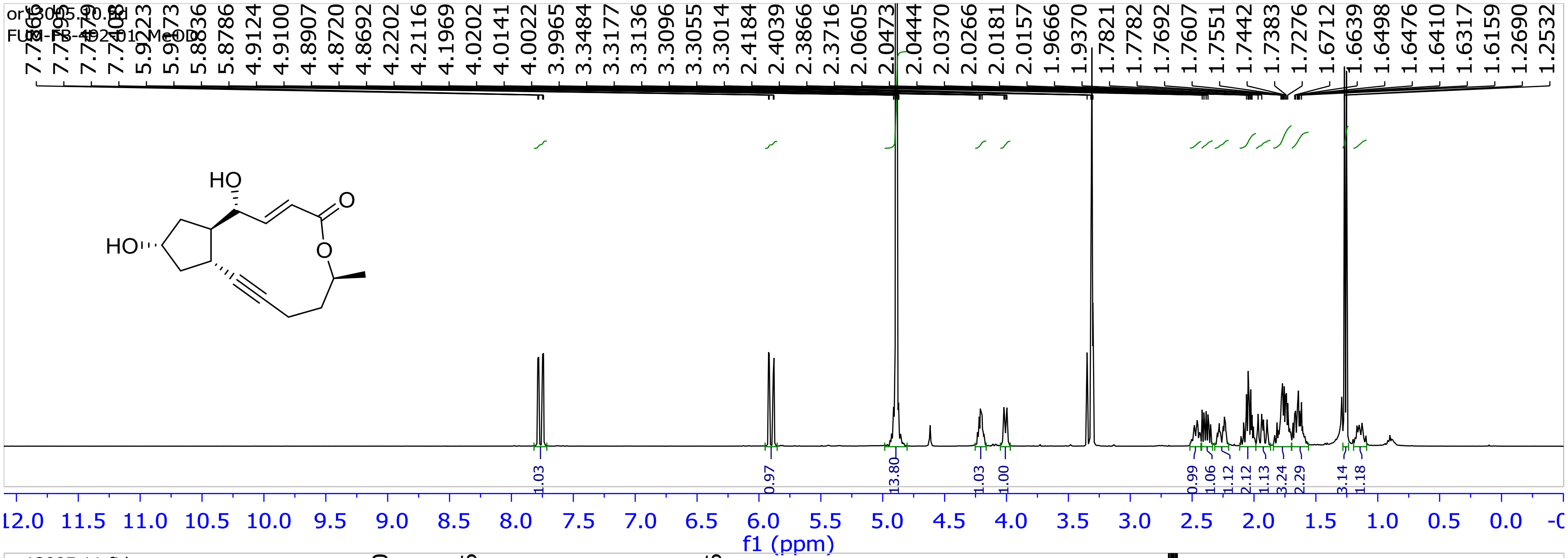

FUM-FB-492-01_MeOD
7.7661
7.7505
7.7770
7.7608
5.9223
5.9073
5.8836
5.8786
4.9124
4.9100
4.8907
4.8720
4.8692
4.2202
4.2116
4.1969
4.0202
4.0141
4.0022
3.9965
3.3484
3.3177
3.3136
3.3096
3.3055
3.3014
2.4184
2.4039
2.3866
2.3716
2.0605
2.0473
2.0444
2.0370
2.0266
2.0181
2.0157
1.9666
1.9370
1.7821
1.7782
1.7692
1.7607
1.7551
1.7442
1.7383
1.7276
1.6712
1.6639
1.6498
1.6476
1.6410
1.6317
1.6159
1.2690
1.2532
HO
HO
O
O
1.03
0.97
13.80
1.03
1.00
0.99
1.06
1.12
2.12
1.13
3.24
2.29
3.14
1.18
f1 (ppm)

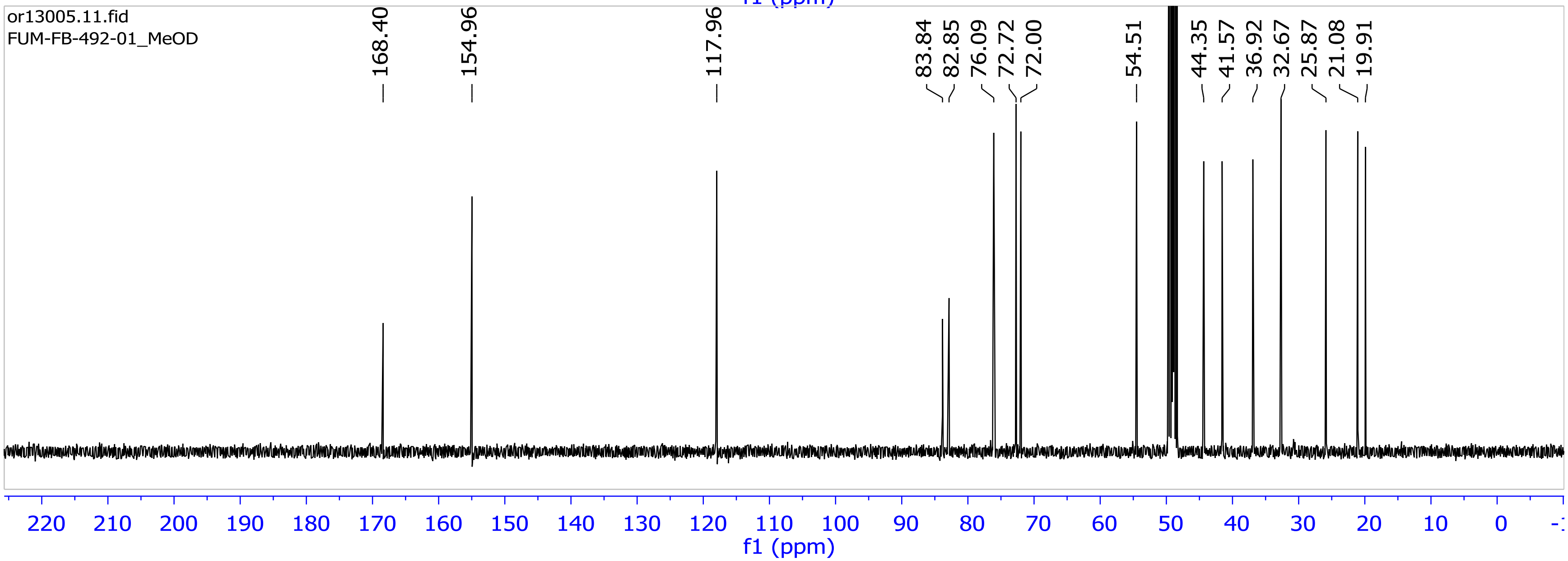

or13005.11.fid
FUM-FB-492-01_MeOD
168.40
154.96
117.96
83.84
82.85
76.09
72.72
72.00
54.51
44.35
41.57
36.92
32.67
25.87
21.08
19.91
f1 (ppm)

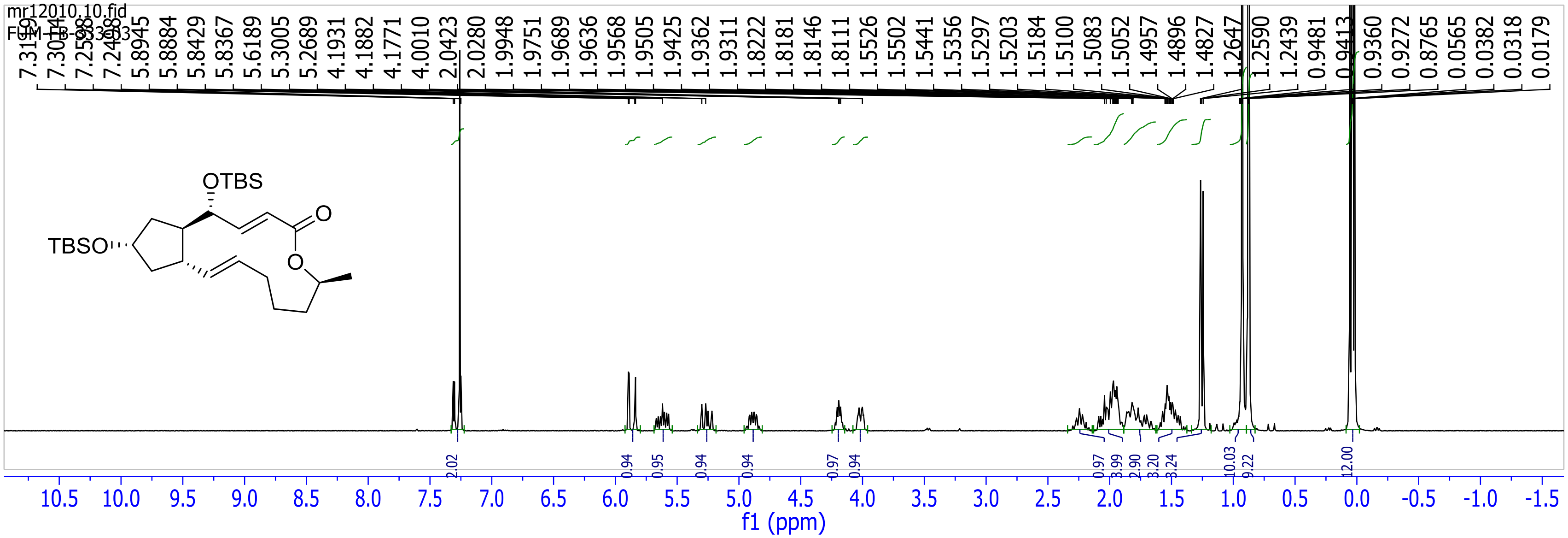
mr12010.10.fid
FUM-FB-333-03
7.3049
7.2538
7.2488
5.8945
5.8884
5.8429
5.8367
5.6189
5.3005
5.2689
4.1931
4.1882
4.1771
4.0010
2.0423
2.0280
1.9948
1.9751
1.9689
1.9636
1.9568
1.9505
1.9425
1.9362
1.9311
1.8222
1.8181
1.8146
1.8111
1.5526
1.5502
1.5441
1.5356
1.5297
1.5203
1.5184
1.5100
1.5083
1.5052
1.4957
1.4896
1.4827
1.2647
1.2590
1.2439
0.9481
0.9413
0.9360
0.9272
0.8765
0.0565
0.0382
0.0318
0.0179
OTBS
TBSO
O
O
2.02
0.94
0.95
0.94
0.94
0.97
0.94
0.97
3.99
2.90
3.20
3.24
10.03
9.22
12.00
f1 (ppm)

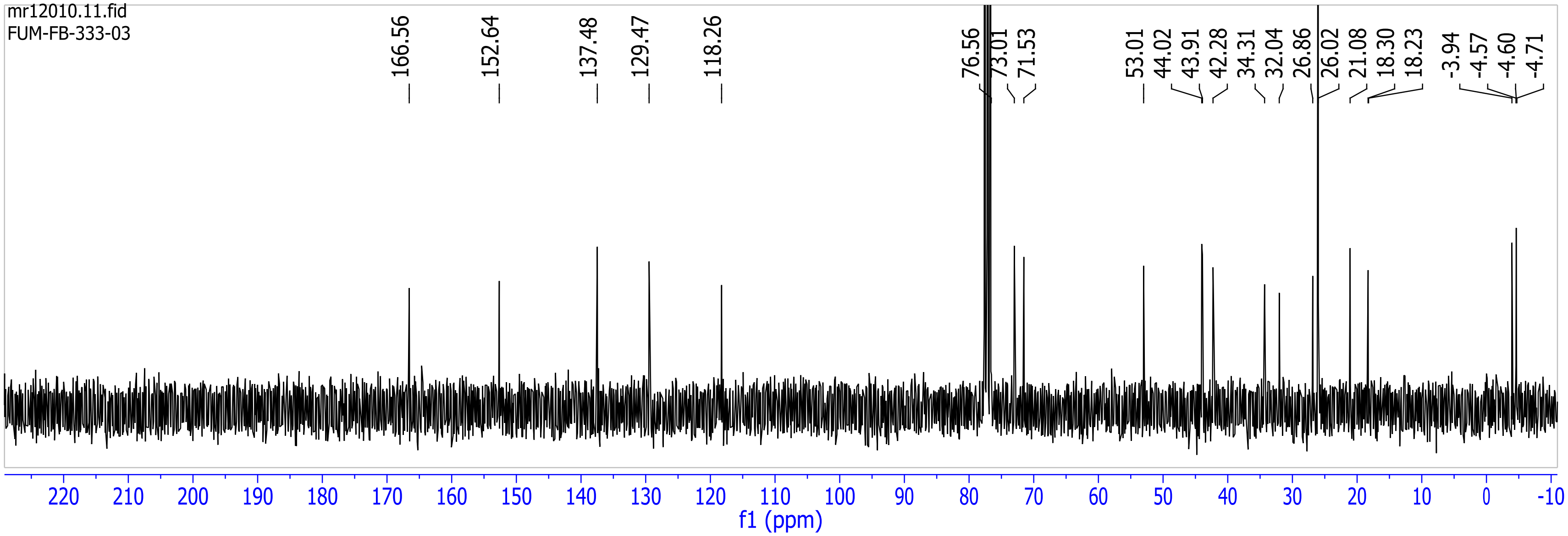
mr12010.11.fid
FUM-FB-333-03
166.56
152.64
137.48
129.47
118.26
76.56
73.01
71.53
53.01
44.02
43.91
42.28
34.31
32.04
26.86
26.02
21.08
18.30
18.23
-3.94
-4.57
-4.60
-4.71
f1 (ppm)

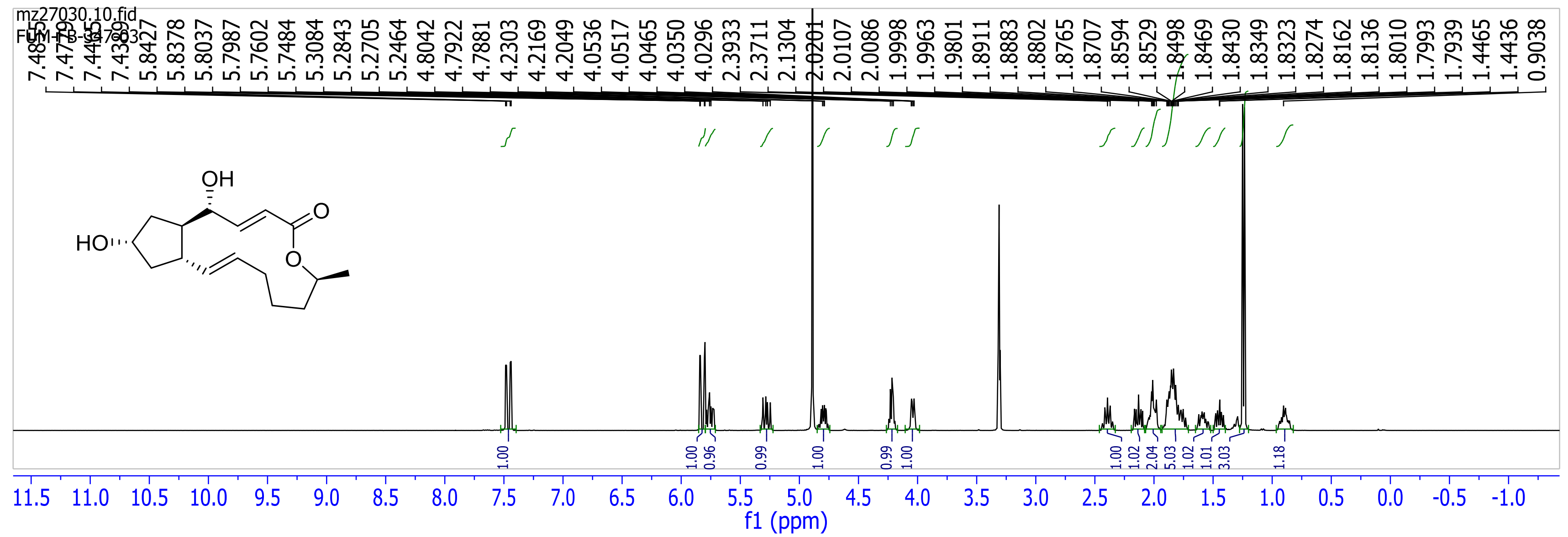
mz27030.10.fid
FUM-FB-347-03
5.8427
5.8378
5.8037
5.7987
5.7602
5.7484
5.3084
5.2843
5.2705
5.2464
4.8042
4.7922
4.7881
4.2303
4.2169
4.2049
4.0536
4.0517
4.0465
4.0350
4.0296
2.3933
2.3711
2.1304
2.0201
2.0107
2.0086
1.9998
1.9963
1.9801
1.8911
1.8883
1.8802
1.8765
1.8707
1.8594
1.8529
1.8498
1.8469
1.8430
1.8349
1.8323
1.8274
1.8162
1.8136
1.8010
1.7993
1.7939
1.4465
1.4436
0.9038
OH
HO
O
O
1.00
1.00
0.96
0.99
1.00
0.99
1.00
1.00
1.02
2.04
5.03
1.02
1.01
3.03
1.18
f1 (ppm)

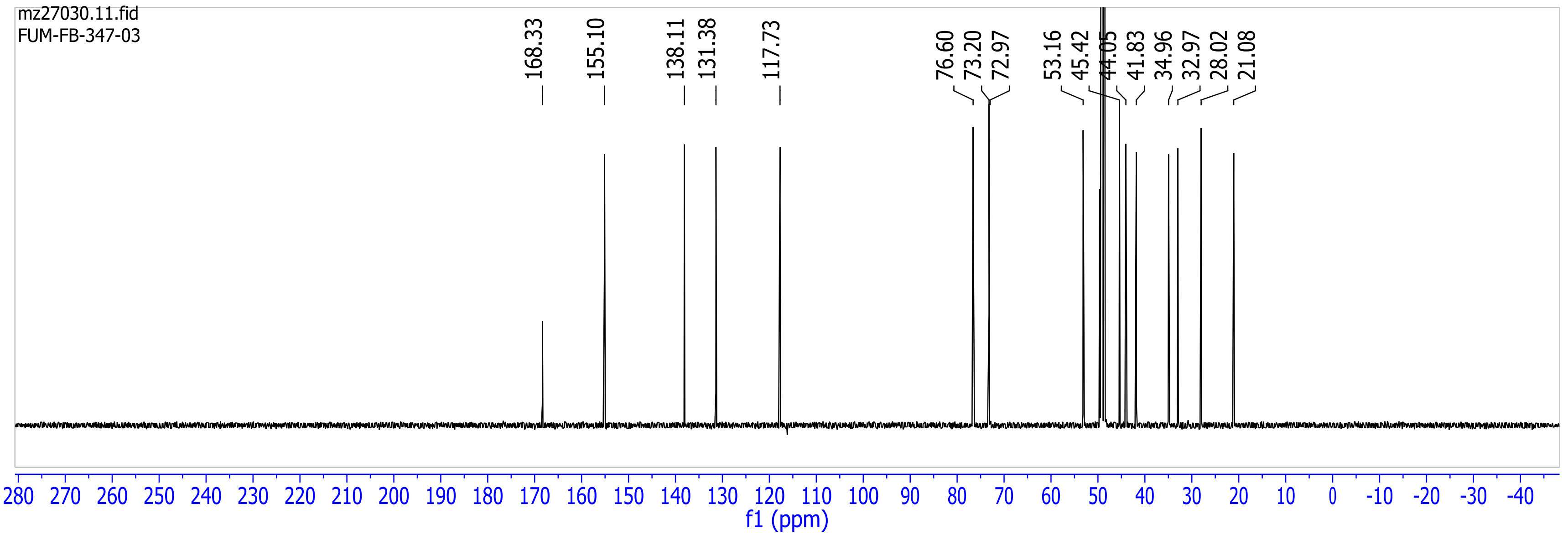
mz27030.11.fid
FUM-FB-347-03
168.33
155.10
138.11
131.38
117.73
76.60
73.20
72.97
53.16
45.42
44.05
41.83
34.96
32.97
28.02
21.08
f1 (ppm)
